# STK4 inhibits the E3 activity of HOIP by phosphorylating its allosteric ubiquitin-binding site

**DOI:** 10.1038/s41421-025-00824-x

**Published:** 2025-09-16

**Authors:** Yaru Wang, Xindi Zhou, Zhiqiao Lin, Yichao Huang, Yuchao Zhang, Haobo Liu, Yuqian Zhou, Jianping Liu, Lifeng Pan

**Affiliations:** 1 School of Chemistry and Materials Science, Hangzhou Institute for Advanced Study, University of Chinese Academy of Sciences, 1 Sub-lane Xiangshan, Hangzhou, Zhejiang China; 2 State Key Laboratory of Chemical Biology, Shanghai Institute of Organic Chemistry, University of Chinese Academy of Sciences, Chinese Academy of Sciences, Shanghai, China

**Keywords:** Ubiquitylation, Nanocrystallography, Phosphorylation

## Abstract

HOIP, an RBR-type E3 ligase and the catalytic subunit of the linear ubiquitin chain assembly complex (LUBAC), plays crucial roles in various cellular processes, including the NF-κB signaling pathway. The E3 activity of HOIP can be inhibited by the kinase STK4-mediated phosphorylation, although the mechanism is poorly understood. In this study, using biochemical, mass spectrometry and structural approaches, we systemically characterize the association of STK4 with HOIP, and unveil that STK4 can directly bind to the RING2-LDD module of HOIP through its kinase domain. The determined crystal structure of STK4 in complex with HOIP RING2-LDD not only elucidates the detailed binding mechanism of STK4 with HOIP, but also uncovers, for the first time, a unique binding mode of STK4 with its substrate. Moreover, we reveal that STK4 can directly phosphorylate the T786 residue of HOIP that is located in the allosteric ubiquitin-binding site of HOIP. Importantly, the phosphorylation of HOIP T786 mediated by STK4 can block the binding of ubiquitin to the allosteric site of HOIP, thereby attenuating the E3 activity of HOIP. In all, our findings provide mechanistic insights into the interaction between STK4 and HOIP as well as the negative regulation of HOIP’s E3 activity by STK4-mediated phosphorylation, which are valuable for further understanding the regulatory modes of RBR-type E3 ligases.

## Introduction

Ubiquitination, a highly important and versatile reversible post-translational modification (PTM) in mammals, participates in almost all vital cellular processes, including protein degradation, DNA repair, gene transcription, autophagy, and innate immunity^1–6^. The ubiquitination of a target substrate is mainly achieved through the cooperative actions of three different enzymes: a ubiquitin (Ub)-activating E1 enzyme, a Ub-conjugating E2 enzyme and a Ub ligase E3 enzyme^3,7,8^. Since Ub can be modified by itself to form poly-Ub chains, there are totally eight different types of homotypic poly-ubiquitination (M1-, K6-, K11-, K27-, K29-, K33-, K48-, and K63-linked type), in addition to mono-ubiquitination^9,10^. M1-linked poly-ubiquitination (also named linear poly-ubiquitination) is a unique type of poly-ubiquitination, in which the poly-Ub chain is formed by Ub units covalently linked by peptide bonds in a head-to-tail manner^11,12^. The linear ubiquitin chain assembly complex (LUBAC) is the only currently known E3 ligase complex that mediates linear Ub chain formation, and is composed of three subunits: the catalytic subunit HOIP, and two regulatory subunits, Sharpin and HOIL-1L^11,13–17^. LUBAC has been demonstrated to play crucial roles in many cellular processes, including NF-κB and Wnt signaling pathways, autophagy, and mitosis^11,16,18–24^. Importantly, dysfunction of LUBAC due to the deficiency of any subunit of LUBAC causes immunodeficiency, inflammation or even death in mice or humans^19,25–28^. Given the critical roles played by LUBAC in various immune and inflammation-related signaling pathways, the activity of LUBAC has been precisely tuned temporally and spatially by other regulatory proteins^29–32^. However, many of the detailed molecular mechanisms underlying LUBAC regulation by these proteins are still not well understood.

**Fig. 1:**
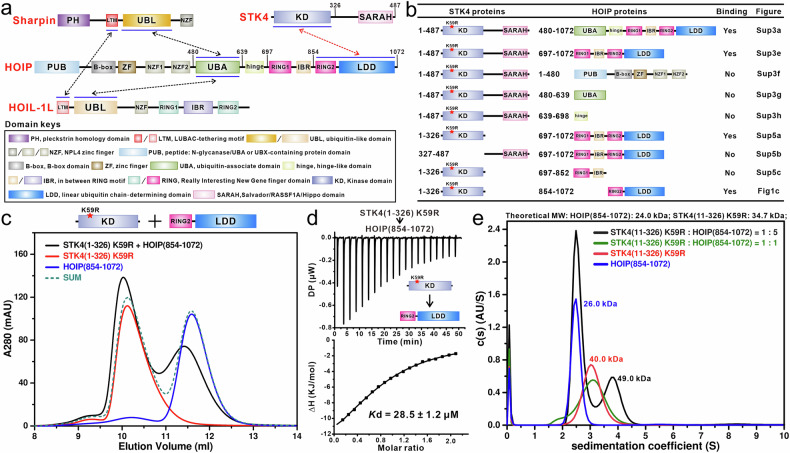
Biochemical characterization of the interaction between HOIP and STK4. **a** Schematic diagram showing the domain organizations of HOIP, HOIL-1L, Sharpin and STK4. In the drawing, the inter-molecular interactions of HOIP, HOIL-1L and Sharpin are indicated by black two-way arrows, while the HOIP/STK4 interaction characterized in this study is highlighted by red two-way arrows. **b** The summarized mapping results of the binding regions between HOIP and STK4 based on our SEC-based analyses. **c** SEC-based analyses of the interaction between 20 μM HOIP RING2-LDD and 30 μM STK4 KD. The “SUM” stands for the theoretical sum of the SEC profiles of STK4 (residues 1–326) K59R and HOIP (residues 854–1072). This assay was performed using a Superdex 75 Increase 10/300 GL column (GE Healthcare). **d** ITC-based measurement of the binding affinity of HOIP RING2-LDD and STK4 KD. The *K*d error is the fitted error obtained from the data analysis software when using the one-site binding model to fit the ITC data; DP, differential power, measured by the ITC machine; ΔH, heat change measured by the ITC machine. **e** Overlay plot of the sedimentation velocity data of HOIP RING2-LDD (blue), STK4 KD (red), and the mixture of HOIP RING2-LDD with STK4 KD in a molar ratio of 1:1 (green) or 1:5 (black); c(s) continuous sedimentation coefficient distribution; MW molecular weight. The relevant experiments depicted in this figure have been replicated three times.

HOIP belongs to the RING-between-RING (RBR) type E3 ligase, characterized by an RBR module that includes RING1, IBR and RING2 domains (Fig. 1a). Previous elegant structural studies have well demonstrated that the RBR and LDD domains of HOIP pack together to form the catalytic core of HOIP for mediating the conjugation of linear Ub chains^33,34^. Specifically, the RING1 domain together with the RING2 and LDD domains of HOIP can recognize the E2 ~ Ub conjugate and catalyze the transfer of Ub from the E2~Ub conjugate to the catalytic Cys residue in the RING2 domain of HOIP^33,34^. Subsequently, Ub continues to translocate and form the linear Ub chain, a process mediated by a unique Ub-binding platform assembled by the RING2-LDD module of HOIP^33,34^. Interestingly, the RING1 and IBR region of HOIP also harbors an allosteric Ub-binding site, where Ub binding can dramatically increase the E3 activity of HOIP^34^. Notably, other RBR-type E3 ligases, such as Parkin and HOIL-1L, also contain similar allosteric Ub-binding sites^35–38^. Given the critical role of this allosteric Ub-binding site for RBR-type E3 ligases, whether the allosteric Ub-binding site of HOIP could be regulated by other types of PTM remains an open question.

Kinase-mediated phosphorylation is one of the most fundamental and pervasive PTMs in biology, playing a critical role in regulating nearly all aspects of cellular processes, from metabolism to signal transduction, gene expression and cell growth^39,40^. Given the pervasive role of phosphorylation in regulating protein activity and interactions, it was expected that phosphorylation could influence the E3 ligase activity of LUBAC. Indeed, the E3 activity of HOIP was recently reported to be regulated by the kinase STK4 (also known as MST1)^32^. Particularly, STK4 can directly phosphorylate HOIP and inhibit its E3 activity, thereby attenuating the LUBAC-mediated activation of the NF-κB signaling pathway^32^. However, the detailed molecular mechanism underpinning the inhibition of HOIP’s E3 activity by STK4-mediated phosphorylation remains elusive.

STK4, a member of the Group II germinal center kinases, plays vital roles in various biological processes in mammals, including cell growth, cell differentiation, stress response, and apoptosis^41,42^. Structurally, STK4 primarily contains an N-terminal conventional kinase domain (KD) and a C-terminal coiled-coil SARAH (Salvador/RASSF1/Hippo) domain (Fig. 1a), which enables STK4 to homodimerize or heterodimerize with other relevant SARAH domain-containing proteins^43,44^. As a serine/threonine kinase, STK4 specifically recognizes and phosphorylates dozens of protein substrates, including Mob1A/Mob1B, Lats1/Lats2, histone H2B, FOXO3 and HOIP, thereby regulating their relevant cellular functions^32,41,45–48^. However, due to the absence of complex structures, the precise mechanism by which STK4 specifically recognizes its substrates remains largely unknown.

Here, we biochemically and structurally characterize the interaction between STK4 and LUBAC, discovering that STK4 directly binds to the RING2-LDD region of HOIP through its KD. We determine the crystal structure of the HOIP RING2-LDD in complex with STK4 KD, which not only elucidates the detailed molecular mechanism governing the specific association of STK4 with LUBAC, but also represents the first atomic structure, to our knowledge, showing how STK4 kinase recognizes its substrate. Additionally, based on our biochemical, mass spectrometry and structural analyses, we demonstrate that STK4 inhibits the E3 activity of HOIP by phosphorylating the T786 residue within HOIP’s allosteric Ub-binding site. In all, our findings uncover the molecular basis underlying the negative regulation of the E3 activity of HOIP by STK4 and unveil a unique regulatory mode for the RBR-type E3 ligase HOIP.

## Results

### STK4 can specifically bind to the RING2-LDD region of HOIP through its KD

To elucidate how STK4 specifically regulates LUBAC activity, we first aimed to biochemically characterize the interaction of STK4 with LUBAC. Given that kinase-active STK4 exists in an inhomogeneous oligomer state^44^, we constructed the K59R mutation of STK4. This mutation, based on previous studies^44,49^, specifically abolishes the kinase activity of STK4, yielding kinase-dead full-length STK4 (residues 1–487) K59R proteins suitable for further biochemical characterization. Using Size Exclusion Chromatography (SEC)-based assays with purified STK4 (residues 1–487) K59R mutant, as well as relevant HOIL-1L and Sharpin proteins, we revealed that the STK4 (residues 1–487) K59R mutant was unable to interact with the full-length HOIL-1L and Sharpin (Supplementary Fig. S1). However, STK4 (residues 1–487) K59R directly bound to the HOIP (residues 480–1072) fragment, the HOIP (residues 480–1072)-containing truncated LUBAC complex, and the HOIP (residues 697–1072) fragment that includes the RING1-IBR-RING2-LDD module (Fig. 1b; Supplementary Figs. S2, S3a–e). Conversely, it did not bind to the HOIP (residues 1–480), HOIP (residues 480–639), or HOIP (residues 639–698) fragments (Fig. 1b; Supplementary Fig. S3f–h). Our isothermal titration calorimetry (ITC)-based analyses revealed that STK4 (residues 1–487) K59R directly binds to HOIP(residues 697–1072) with a dissociation constant (*K*d) value of ∼102 μM (Supplementary Fig. S4a). Importantly, the HOIP (residues 854–1072) fragment, containing only the RING2 and LDD domains, also bound to STK4 (residues 1–487) K59R with a similar *K*d value (Supplementary Fig. S4). These findings collectively indicated that the RING2-LDD region of HOIP is responsible for interacting with STK4.

Subsequently, using SEC-based assays, we also mapped the HOIP-binding region of STK4. Our results revealed that the KD, rather than the SARAH domain, of STK4 directly interacts with HOIP (residues 697–1072) (Fig. 1b; Supplementary Fig. S5a, b). Further truncation analyses confirmed that STK4 (residues 1–326) K59R directly binds to the RING2-LDD module but not the RING1-IBR region of HOIP (Fig. 1c; Supplementary Fig. S5c). Concurrently, our quantitative ITC analyses confirmed that STK4 (residues 1–326) K59R directly binds to HOIP (residues 854–1072) (RING2-LDD) and HOIP (residues 697–1072) (RING1-IBR-RING2-LDD) with similar binding affinities (Fig. 1d; Supplementary Fig. S6a). Notably, the binding affinity of STK4 (residues 1–487) K59R with HOIP (residues 854–1072) is much weaker than that of the STK4 (residues 1–326) K59R/HOIP (residues 854–1072) interaction (Fig. 1d; Supplementary Fig. S4b), suggesting that other regions of STK4 can partially inhibit its KD from binding to HOIP, consistent with previous findings that full-length STK4 exists in an auto-inhibited state^44^. To facilitate our structural studies, we further narrowed down the KD region of STK4 for HOIP binding using ITC-based analyses. Our ITC results showed that STK4 (residues 11–326) K59R (hereafter referred to as STK4 KD), which lacks the N-terminal unconserved 10 residues of STK4 (residues 1–326) K59R (Supplementary Fig. S7), still interacts well with HOIP compared with STK4 (residues 1–326) K59R (Fig. 1d; Supplementary Fig. S6). Meanwhile, analytical ultracentrifugation-based analyses revealed that STK4 KD exists in a concentration-dependent monomer-dimer equilibrium (Supplementary Fig. S8), while HOIP (residues 854–1072) is a stable monomer in solution (Fig. 1e). Remarkably, STK4 KD can directly interact with HOIP(residues 854–1072) to form an unstable binary complex (Fig. 1e), likely due to the weak and dynamic nature of their interaction. In summary, our biochemical results clearly demonstrated that STK4 directly binds to HOIP through a specific interaction between STK4 KD and the HOIP RING2-LDD region.

### Overall structure of HOIP RING2-LDD in complex with STK4 KD

To uncover the mechanistic basis underlying the specific interaction between HOIP RING2-LDD and STK4 KD, we decided to solve the atomic structure of the HOIP (residues 854–1072)/STK4 KD complex using X-ray crystallography. However, conventional purification methods failed to yield a pure HOIP (residues 854–1072)/STK4 KD complex, likely due to the dynamic nature of the interaction between HOIP RING2-LDD and STK4 KD. Therefore, we only mixed HOIP (residues 854–1072) and STK4 KD in a 1:1 molar ratio, and conducted relevant crystal screening using this protein mixture. Fortunately, this approach successfully yielded good crystals that diffracted to a 2.8 Å resolution (Supplementary Table S1).

**Fig. 2:**
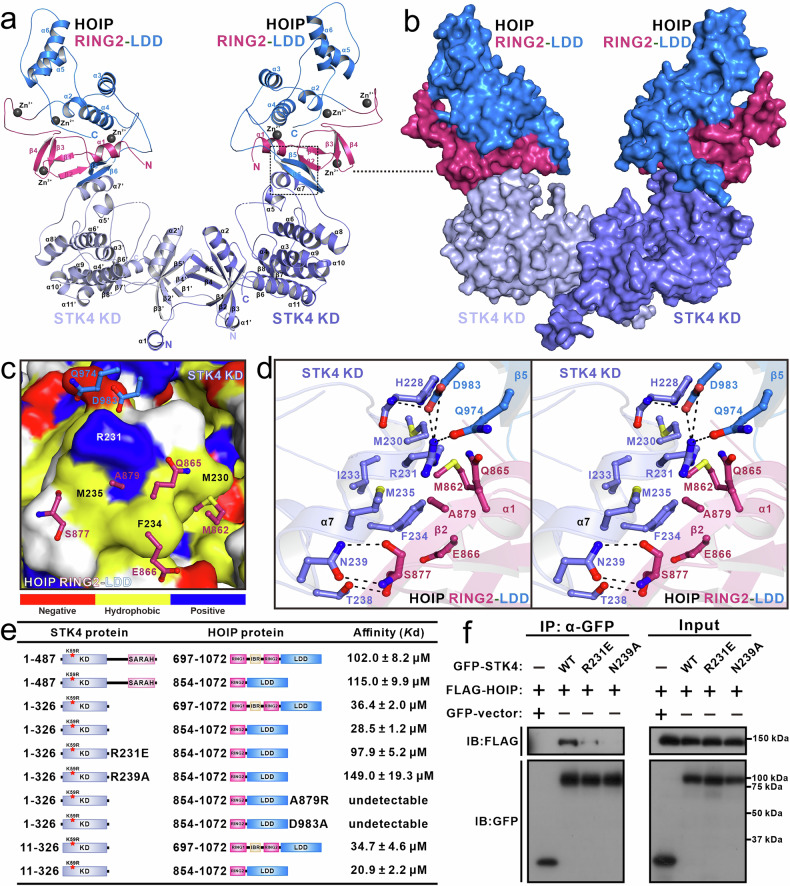
Structural analyses of the HOIP RING2-LDD in complex with STK4 KD. **a** Ribbon diagram showing the overall structure of the HOIP RING2-LDD/STK4 KD complex. **b** Surface representation of the HOIP RING2-LDD/STK4 KD complex. **c** The combined surface representation and the ribbon-stick model showing the hydrophobic interaction interface between HOIP RING2-LDD and STK4 KD. In this presentation, the STK4 KD is shown in the surface model, and the key residues of HOIP RING2-LDD involved in the interaction between them are shown in the stick-ball model. The hydrophobic amino acid residues in the surface model of STK4 KD are drawn in yellow, the positively charged residues in blue, the negatively charged residues in red, and the uncharged polar residues in gray. **d** Stereo view of the ribbon-stick model showing the detailed interactions between HOIP RING2-LDD and STK4 KD. In this drawing, the relevant side chains as well as backbone groups of the key binding interface residues are shown in the stick-ball mode, and the related hydrogen bonds and salt bridges involved in the binding are shown as dotted lines. **e** Summary of the binding affinities between HOIP RING2-LDD and STK4 (residues 1–326) K59R or their mutants measured by ITC-based analyses. **f** Mutagenesis-based Co-IP assay to confirm the interaction between HOIP and STK4 observed in the determined complex structure. IB, immunoblot. The experiments have been replicated three times.

Subsequently, we determined the crystal structure of this HOIP RING2-LDD/STK4 KD complex using the molecular replacement method with modified structures of HOIP RING2-LDD (PDB ID: 4LJO) and STK4 KD (PDB ID: 3COM) serving as search templates (Supplementary Table S1). In the final crystal structure, each asymmetric unit contains one STK4 KD dimer and two HOIP RING2-LDD molecules, forming a symmetrical butterfly-like hetero-tetramer (Fig. 2a, b). Intriguingly, within the HOIP RING2-LDD/STK4 KD complex, the two STK4 KD monomers, each adopting a canonical KD-fold consisting of an N-lobe and C-lobe (but lacking an ATP molecule due to the K59R mutation), directly pack with each other to form a symmetrical dimer through their N-lobes (Fig. 2a, b).

The overall structure of STK4 KD in the HOIP RING2-LDD/STK4 KD complex is highly similar to that of the previously solved active STK4 KD (PDB ID: 3COM) (Supplementary Fig. S9a). However, STK4 KD in our complex possesses additional N- and C-terminal extension structures. Furthermore, several residues but not the key “DFG” motif within the activation loop are missing in STK4 KD of the HOIP RING2-LDD/STK4 KD complex, likely due to the flexible conformation of the activation loop (Supplementary Fig. S9a). In parallel, the RING2 and LDD modules of each HOIP RING2-LDD pack together to form a unique architecture that is mainly composed of six α-helices and six β-strands, and coordinates four zinc ions (Fig. 2a). Specifically, through its RING2 region together with the β5 and β6 strands within the LDD domain, HOIP RING2-LDD specifically binds to the C-lobe of STK4 KD (Fig. 2a, b). Structural comparison analyses showed that the overall structure of HOIP RING2-LDD in the HOIP RING2-LDD/STK4 KD complex is similar to that of HOIP RING2-LDD in the HOIP RING2-LDD/mono-Ub complex (PDB ID: 4LJO)^33^, or the HOIP RBR-LDD/UBE2D2~Ub complex (PDB ID: 5EDV)^34^, except the N-terminal α1-helix region of HOIP RING2, which is directly involved in the binding with STK4 (Supplementary Fig. S9b, c).

### The molecular interface of the HOIP RING2-LDD/STK4 KD complex

Detailed structure analyses uncovered that the binding interface between HOIP RING2-LDD and STK4 KD is primarily formed by residues from the α7-helix of STK4 KD, which accommodates HOIP residues located in the α1-helix and β2-strand of HOIP RING2, as well as the β5- and β6-strands of HOIP LDD through both hydrophobic and polar interactions (Fig. 2a–d; Supplementary Fig. S10). This interface buries a total area of ~553.8 Å (Fig. 2b). Specifically, the side chains of S877, Q974 and D983 of HOIP form hydrogen bonds with the side chains of STK4 T238 and R231, and the backbone amide group of STK4 H228, respectively. Moreover, the side chain of STK4 N239 couples with the backbone carbonyl group and amino group of HOIP S877 to form two highly specific hydrogen bonds (Fig. 2d). In addition, the hydrophobic side chain of HOIP M862 packs against the hydrophobic patch formed by the M230 and F234 residues of STK4, and the aliphatic side chain groups of HOIP Q865 and E866 form hydrophobic contacts with the hydrophobic side chain of the STK4 F234 residue (Fig. 2c, d). Concurrently, the hydrophobic side chain of HOIP A879 inserts into the hydrophobic pocket formed by the hydrophobic side chains of HOIP F234 and M235, as well as the aliphatic side chain groups of HOIP R231 (Fig. 2c, d). Moreover, an Arg-Glu pair (Arg^231^_STK4_-Glu^983^_HOIP_) of salt bridge further strengthens the HOIP RING2-LDD/STK4 KD interaction (Fig. 2d; Supplementary Fig. S10).

In line with their important structural roles, all key interface residues of HOIP and STK4 are highly conserved throughout evolution (Supplementary Figs. S2, S7). Using SEC- and ITC-based analyses, we validated the specific interactions between HOIP RING2-LDD and STK4 KD observed in the complex structure. Consistent with our aforementioned structural data, the results showed that point mutations of key interface residues either from HOIP RING2-LDD or STK4 KD, such as the D983A and A879R mutations of HOIP, or the R231E and N239A mutations of STK4, significantly reduced or essentially abolished the specific interaction between HOIP RING2-LDD and STK4 KD (Fig. 2e; Supplementary Figs. S11, S12). In line with these in vitro biochemical results, our Co-IP experiments confirmed that point mutations of key interface residues, specifically the R231E and N239A mutations of STK4, largely attenuate the specific interaction of STK4 and HOIP in cells (Fig. 2f)

### The dimerization interface of STK4 KD in the HOIP RING2-LDD/STK4 KD complex

Consistent with our aforementioned biochemical result that STK4 K59R can form a dimer in solution (Supplementary Fig. S8), further crystallographic symmetry analysis revealed that STK4 KD exists as a homo-dimer in the crystal structure of the HOIP RING2-LDD/STK4 KD complex, with a total dimerization surface area of ~1103 Å^2^ (Fig. 2a, b). Detailed structural analysis uncovered that the dimerization interface of STK4 KD is primarily formed by residues from the β1, β2, β3, β4, β5 strands, as well as the α2 helix (the conventional αC-helix in the kinase field) located in the N-lobe of STK4 KD through extensive hydrophobic and polar interactions (Supplementary Fig. S13a–c). In particular, a hydrophobic patch formed by the hydrophobic side chain groups of L67, I71, I74, Y88, F93, and L98 residues, together with a hydrophobic patch assembled by the hydrophobic side chain groups of L22, T23, V29, F30, H49, V56, Y89 and the aliphatic side chain groups of K24, E27 residues of STK4, pack against their counterparts in other STK4 to form two large hydrophobic interfaces of STK4 dimer (Supplementary Fig. S13a, b). In parallel, the dimer formation of STK4 is stabilized by several hydrogen bonds formed between the side chains of T23, E27, Q54, S75, and Q78 from one chain of the STK4 dimer and their corresponding counterparts in the other chain (Supplementary Fig. S13a, c).

In line with this structural data, our analytical ultracentrifugation analyses showed that mutating the STK4 F93 residue, located at the dimerization interface of STK4 KD, to a polar Asn residue can attenuate the dimerization ability of STK4 KD in solution, especially under low concentration conditions (Supplementary Fig. S13d, e). Notably, a similar dimerization mode was also observed in the previously determined structure of STK3 KD in complex with a selective inhibitor^50^ (Supplementary Fig. S13f), suggesting a common dimerization mechanism shared by the KDs of STK4 and STK3. Based on a previous study^32^, STK4 can directly phosphorylate HOIP. To test whether the dimerization of STK4 KD domain could affect HOIP phosphorylation mediated by STK4, we performed relevant in vitro phosphorylation assays. Our results showed that the F93N mutant of either full-length STK4 or STK4 KD, which loses the dimerization ability of the KD domain, can still phosphorylate HOIP effectively, similar to wild-type (WT) STK4 (Supplementary Fig. S14). This data suggested that the dimerization of STK4 KD is not essential for the in vitro phosphorylation of HOIP mediated by STK4.

### The phosphorylation of HOIP S1066 does not affect the E3 activity of HOIP in vitro

A recent study reported that STK4 can directly phosphorylate the S1066 residue of HOIP, thereby interfering with Ub recognition by HOIP LDD and inhibiting the E3 activity of LUBAC^32^. Unexpectedly, using in vitro linear ubiquitination assays, we revealed that the phosphomimic S1066E mutant of HOIP (residues 697–1072) exhibits a similar ability to catalyze the formation of linear ubiquitin chains as WT HOIP (residues 697–1072) (Supplementary Fig. S15a). Furthermore, our in vitro linear ubiquitination results using either WT or the S1066A mutant of HOIP (residues 697–1072) showed that STK4 can inhibit the E3 activities of both constructs (Supplementary Fig. S15b, c). This suggests that the phosphorylation of other HOIP sites, rather than S1066, is responsible for the STK4-imposed inhibition of HOIP’s E3 activity. Importantly, using SEC-based assays, we confirmed that the phosphomimic S1066E mutant of HOIP (residues 697–1072) can interact well with linear di-Ub (M1-2Ub) or tetra-Ub (M1-4Ub) (Supplementary Fig. S16), which contrasts with the previously reported result^32^. Based on these findings, we concluded that the phosphorylation of HOIP S1066 is unlikely to affect the E3 activity of HOIP.

### The phosphorylation of HOIP T786 is mainly responsible for the inhibition of the E3 activity of HOIP by STK4

**Fig. 3:**
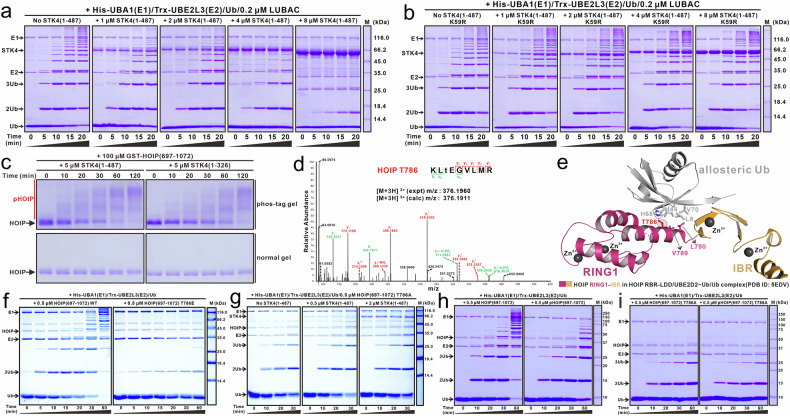
Phosphorylation of HOIP T786 residue mediated by STK4. **a**, **b** In vitro linear ubiquitination assays showing the catalytic activity of the LUBAC complex can be blocked by the WT STK4 (**a**) but not the STK4 K59R mutant (**b**). The LUABC complex used in this assay consists of HOIP (residues 480–1072), the full-length HOIL-1L C460A mutant and the full-length Sharpin purified from *E coil*. **c** In vitro phosphorylation assays showing that HOIP (residues 697–1072) can be specially phosphorylated by the WT STK4 (residues 1–487) or STK4 (residues 1–326). The gels in the lower panels are normal SDS-PAGE gels, while those in the upper panels are phos-tag gels. In these panels, p-HOIP stands for phosphorylated HOIP (residues 697–1072). **d** This figure shows a higher-energy collisional dissociation (HCD) MS/MS spectrum recorded on the [M + 3H]^3+^ ion at m/z 376.1960 of the HOIP peptide KLtEGVLMR harboring one phosphorylated site (denoted by lowercase t). Predicted b- and y-type ions (not including all) are listed above and below the peptide sequence, respectively. Ions observed are labeled in the spectrum, revealing that HOIP T786 is phosphorylated. Notably, the KD of STK4 was used to phosphorylate HOIP (residues 697–1072) for the MS-based identification of phosphorylation sites on HOIP. **e** Cartoon combined with the stick-ball model of Ub binding to HOIP RING1-IBR (PDB ID: 5EDV) showing that the phosphorylation of HOIP T786 residue should affect the Ub binding to the allosteric site of HOIP RING1-IBR. Meanwhile, the T786, V789, L790 residues of HOIP and the L8, I44, V70 and H68 residues of ubiquitin are shown in the stick-ball mode. **f** In vitro linear ubiquitination assays showing the abilities of the WT HOIP (residues 697–1072) and the phosphomimic HOIP (residues 697–1072) T786E mutant to assemble linear ubiquitin chains. Notably, the T786E mutation of HOIP (residues 697–1072) largely reduces the E3 activity of HOIP. **g** In vitro ubiquitination assays in the presence of STK4 showing that the E3 activity of HOIP T786A mutant is unable to be suppressed by STK4-mediated phosphorylation. **h**, **i** In vitro ubiquitination assays using the WT HOIP (residues 697–1072) (**h**) the HOIP (residues 697–1072) T786A mutant (**i**) or their phosphorylated counterparts, showing that the E3 activity of HOIP is suppressed by STK4-mediated phosphorylation rather than STK4-binding. The relevant experiments depicted in this figure have been replicated three times.

Our study revealed that STK4 KD can directly interact with the RING2-LDD of HOIP (Fig. 2). Interestingly, structural comparison analysis revealed that the binding of STK4 KD to the RING2-LDD of HOIP should hinder the binding of the donor Ub from the E2~Ub conjugate to HOIP due to the potential steric exclusion (Supplementary Fig. S17). However, using in vitro ubiquitination assays, we demonstrated that WT STK4 effectively inhibits the E3 activity of LUBAC, while kinase-dead K59R and T183A mutants of STK4 do not (Fig. 3a, b; Supplementary Fig. S18). Thus, the suppression of HOIP’s E3 activity by STK4 is primarily mediated by STK4’s kinase activity, rather than simply its binding.

To identify the key phosphorylation site on HOIP responsible for STK4-mediated suppression of E3 activity of HOIP, we first performed relevant in vitro phosphorylation assays. The results showed that both full-length STK4 and STK4 (residues 1–326) can effectively phosphorylate the HOIP (residues 697–1072) fragment (Fig. 3c; Supplementary Fig. S19a). Subsequent mass spectrometry-based analysis confidently identified 12 phosphorylation sites on the HOIP (residues 697–1072) fragment, phosphorylated by either full-length STK4 or STK4 (residues 1–326): T715, T720, T731, T756, T764, S772, T786, T823, S840, T891, T955, and S1066 (Fig. 3d; Supplementary Fig. S19b, c). Notably, STK4 can also directly phosphorylate the HOIP (residues 480–1072) subunit within the LUBAC complex (Supplementary Fig. S19d). Mass spectrometry analyses confirmed that all 12 phosphorylation sites identified in isolated HOIP (residues 697–1072) were also present in the STK4-phosphorylated HOIP (residues 480–1072) subunit of the LUBAC complex (Supplementary Fig. S19e). Interestingly, careful structural analyses of the 12 phosphorylation sites of HOIP (residues 697–1072) revealed that the T786 site is located within the allosteric Ub-binding site of RING1 (Fig. 3e; Supplementary Fig. S19f). The binding of a Ub entity to this site is known to dramatically enhance the E3 activity of HOIP^34^. Our sequence alignment analysis revealed that the T731, T764, T786, S840, T891 and T955 residues of HOIP are highly conserved across different eukaryotic species (Supplementary Fig. S2). Therefore, we just focused on these six conserved residues for further characterization. Using in vitro ubiquitination assays with relevant phosphomimic mutants, we demonstrated that the S840E and T955E mutations of HOIP (residues 697–1072) had little effect on the E3 activity of HOIP, while the T731E, T764E, T786E and T891E mutations of HOIP (residues 697–1072) significantly reduced HOIP’s E3 activity for catalyzing linear ubiquitin chain formation (Fig. 3f; Supplementary Fig. S20). Thus, we preliminarily identified the HOIP T731, T764, T786 and T891 residues as potential key phosphorylation sites mediated by STK4.

We then individually mutated these four residues to an unphosphorylable alanine residue. Using in vitro ubiquitination assays, we found that only the E3 activity of HOIP (residues 697–1072) T786A could not be effectively inhibited by STK4 (Fig. 3g; Supplementary Figs. S21, S22). It is worthwhile noting that the HOIP T731A and T786A mutants exhibited lower activities than their WT counterpart in our in vitro ubiquitination assays (Supplementary Figs. S21, S22a, b). Relevant structural analysis revealed that HOIP T731 is located within the α1-helix of HOIP RING1, and its hydroxyl side chain group forms a specific hydrogen bond with the backbone group of R727, stabilizing the formation of the RING1 α1-helix, which directly participates in the interaction with E2 in the HOIP RBR-LDD/UBE2D2~Ub/Ub complex structure (PDB ID: 5EDV) (Supplementary Fig. S23a). Thus, we speculated that the T731A mutation of HOIP might affect the interaction of HOIP RING1 with E2, thereby decreasing the enzymatic activity of HOIP for assembling linear ubiquitin chains. Indeed, our SEC-based analyses revealed that the T731A mutation of HOIP RING1 (residues 697–793) caused a detectable reduction of the interaction of HOIP RING1 with UBE2L3 (Supplementary Fig. S23b–d). Meanwhile, based on our structural analysis, the HOIP T786 residue directly participates in the interaction of HOIP RING1 with the allosteric ubiquitin (Fig. 3e). Therefore, the T786A mutation should impair the binding ability of HOIP for the allosteric ubiquitin, consequently affecting the activation of the linear ubiquitination activity of HOIP. In line with our biochemical data, structural modeling analysis showed that the RBR-LDD module of HOIP exhibits considerable conformational flexibility (Supplementary Fig. S24). This flexibility allows relative movement between its N-terminal RING1-IBR module and C-terminal RING2-LDD region, thereby permitting the T786 residue within HOIP RING1 to be accessible for STK4 KD-mediated phosphorylation (Supplementary Fig. S24).

Finally, using purified phosphorylated HOIP (residues 697–1072) and HOIP (residues 697–1072) T786A mutant proteins (Supplementary Fig. S25), we demonstrated that the E3 activity of the phosphorylated HOIP (residues 697–1072), but not the phosphorylated HOIP (residues 697–1072) T786A mutant, was largely reduced compared with that of the un-phosphorylated HOIP counterpart (Fig. 3h, i). This directly revealed that STK4 primarily inhibits HOIP’s E3 activity through STK4-mediated phosphorylation of HOIP T786. In conclusion, our findings indicated that phosphorylation of HOIP T786, rather than S1066, is mainly responsible for the STK4-mediated inhibition of HOIP’s E3 activity.

### The T786 phosphorylation site of HOIP is critical for regulating the NF-κB signaling

**Fig. 4:**
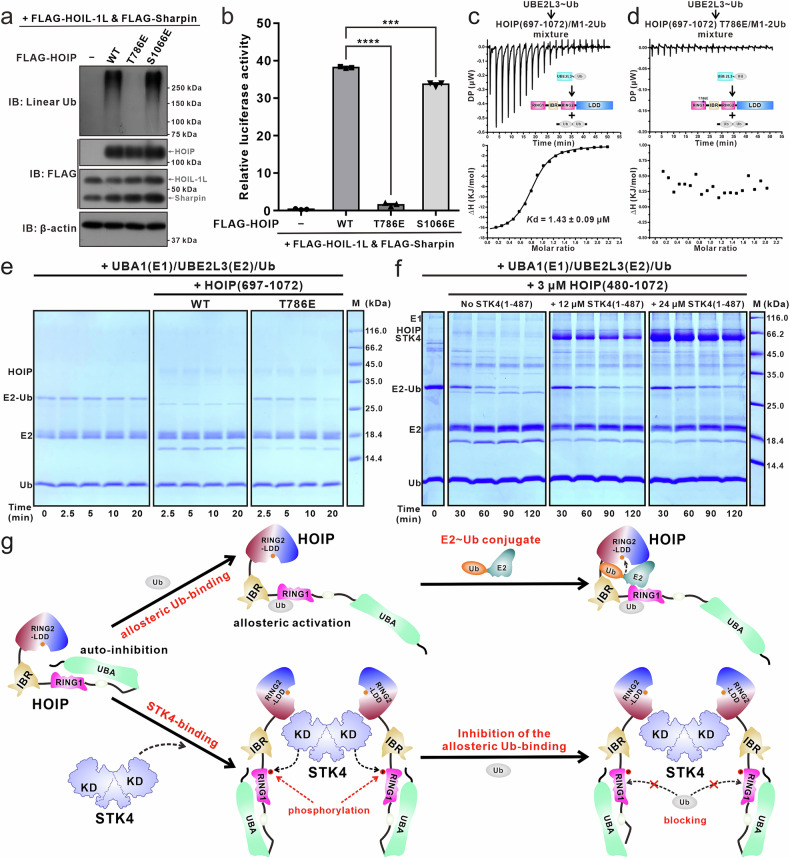
STK4-mediated phosphorylation of HOIP T786 attenuates the intracellular NF-κB signaling and the transfer of ubiquitin from the E2~Ub conjugate to the catalytic cysteine of HOIP. **a** In vivo linear ubiquitination assay showing the abilities of the WT HOIP, the phosphomimetic T786E and S1066E mutants of HOIP to assemble linear Ub chains in HeLa cells when co-expressing with HOIL-1L and Sharpin. **b** NF-κB reporter dual-luciferase assays using overexpressed HOIL-1L and Sharpin without or with the WT HOIP, the HOIP T786E mutant or the HOIP S1066E mutant in HEK293T cells. Error bars denote the SEM between three replicates. An unpaired Student’s *t*-test analysis was used to define a statistically significant difference, and the stars indicate significant differences between the indicated bars (***P < 0.001; ****P < 0.0001). **c**, **d** ITC-based measurement of the binding affinity of the UBE2L3-Ub conjugate with the WT HOIP (residues 697–1072) (**c**) or the HOIP (residues 697–1072) T786E mutant (**d**) in the presence of M1-2Ub with the molar ratio of 1:4. **e** E2~Ub discharge assays confirming that the T786E mutation of HOIP decelerates the transfer of ubiquitin from E2~Ub conjugate to the catalytic cysteine residue of HOIP. **f** E2~Ub discharge assays combined with in vitro phosphorylation assays showing the transfer of ubiquitin from E2~Ub conjugate to the catalytic cysteine residue of HOIP can be attenuated by STK4-mediated phosphorylation of HOIP. **g** A schematic cartoon diagram summarizing the regulatory mechanism of HOIP by STK4-mediated phosphorylation. The experiments depicted in this figure have been replicated three times.

To test whether the identified T786 phosphorylation site of HOIP is relevant in cells, we conducted several cellular assays to investigate its role in the NF-κB signaling. Consistent with previous studies^14,15^, co-expression of WT HOIP with HOIL-1L and Sharpin generated large amounts of M1-linked Ub chains in HeLa cells (Fig. 4a). Strikingly, co-expression of the phosphomimic HOIP T786E mutant, but not the HOIP S1066E mutant, with HOIL-1L and Sharpin, was essentially unable to promote the formation of the M1-linked Ub chains in HeLa cells (Fig. 4a).

Consistently, luciferase-based NF-κB reporter assays in HEK293T cells revealed that the phosphomimic T786E mutation, but not the S1066E mutation, largely reduced HOIP’s ability to promote NF-κB activation when co-expressed with HOIL-1L and Sharpin (Fig. 4b). These results revealed that the phosphomimetic T786E mutation of HOIP can suppress the E3 activity of HOIP and significantly reduce NF-κB signaling. Notably, although the phosphomimetic HOIP S1066E mutant exhibited a similar ability to assemble linear Ub chains as WT HOIP, it displayed a slightly reduced ability to promote the activation of NF-κB signaling (Fig. 4b), in line with a previous report^32^. Taken together, these data clearly demonstrated that the T786 phosphorylation site of HOIP plays a critical role in regulating NF-κB signaling.

### Phosphorylation of HOIP T786 suppresses the E3 activity of HOIP by disrupting allosteric ubiquitin binding

Then, we sought to reveal why HOIP T786 phosphorylation suppresses HOIP’s E3 activity. Given that the T786 residue is located in the allosteric Ub-binding site of HOIP (Fig. 3d), we inferred that the phosphorylation of HOIP T786 might affect the interaction between HOIP and allosteric Ub. As it is challenging to obtain solely T786 phosphorylated HOIP for biochemical characterization, we used the phosphomimic HOIP T786E mutant as an alternative. Using SAXS assays combined with structural modeling analyses based on AlphaFold3^51^, we first confirmed that the phosphomimic T786E mutation did not significantly alter the structural folding of HOIP RBR-LDD (Supplementary Fig. S26). Subsequently, SEC-based assays with the WT or the phosphomimic T786E mutant of HOIP (residues 697–852), which only includes the RING1 and IBR modules of HOIP, revealed that the T786E mutation of HOIP (residues 697–852) reduces the interaction between HOIP (residues 697–852) and M1-8Ub, a polyubiquitin chain known to bind to HOIP’s allosteric Ub-binding site (Supplementary Fig. S27).

Notably, the binding of a Ub entity to the allosteric Ub-binding site of HOIP can stabilize HOIP’s active conformation, thereby facilitating the binding of the E2~Ub conjugate to HOIP^34^. Consistent with this, our ITC-based analyses showed that the phosphomimic T786E mutation of HOIP essentially abolishes the interaction between HOIP (residues 697–1072) and UBE2L3~Ub in the presence of M1-2Ub (Fig. 4c, d). Moreover, using the E2~Ub discharge assays, we demonstrated that the phosphomimic T786E mutation of HOIP can effectively decrease the transfer of Ub from the E2~Ub conjugate to the catalytic cystine residue of HOIP (Fig. 4e). Importantly, in vitro phosphorylation combined with E2~Ub discharge assays revealed that STK4-mediated phosphorylation of HOIP (residues 480–1072) inhibits the transfer of ubiquitin from the E2~Ub conjugate to the E3 HOIP (Fig. 4f). Collectively, these results clearly demonstrated that STK4-mediated phosphorylation of HOIP at the T786 residue decreases the transfer of Ub from the E2~Ub conjugate to HOIP, thereby inhibiting the E3 activity of HOIP.

## Discussion

A previous cell-based functional study reported that the STK4 kinase can directly phosphorylate the S1066 residue of HOIP to negatively regulate the E3 activity of HOIP for forming linear Ub chains^32^. However, due to the lack of detailed biochemical and structural characterizations, the precise mechanism by which STK4 specifically recognizes HOIP and inhibits the E3 activity of HOIP via S1066 phosphorylation remained unclear. Based on our structural analyses, the S1066 residue of HOIP is located in the extreme C-terminal loop of HOIP LDD, and is far away from the donor Ub and acceptor Ub-binding sites of HOIP RING2-LDD, as well as the allosteric Ub and the E2~Ub conjugate-binding sites of HOIP RBR-LDD (Supplementary Fig. S28). Therefore, in contrast to the notion proposed in the previous study^32^, phosphorylation of HOIP S1066 is unlikely to interfere with the recognition of the acceptor Ub by HOIP LDD. In this study, through systematically biochemical and structural characterizations, we demonstrated that the phosphorylation of HOIP T786, rather than S1066, is primarily responsible for STK4-mediated inhibition of HOIP’s E3 activity. Our mechanistic studies further uncovered that the T786 residue is located in the allosteric Ub-binding site of HOIP RING1. STK4-mediated phosphorylation of this site directly interferes with allosteric ubiquitin binding of HOIP, thereby decreasing the efficiency of Ub transfer from the E2~Ub conjugate to HOIP, which ultimately inhibits the E3 activity of HOIP. Recently, increasing evidences showed that RBR-type E3 ligases typically exist in auto-inhibited states, and allosteric activation by Ub or Ub-like molecules is likely a common feature for many RBR-type E3 ligases^34,36,37,52^. To our knowledge, there is no reported case showing that the allosteric Ub-binding site of an RBR-type E3 ligase can be regulated by PTMs. Therefore, this study also uncovered an unprecedented regulatory mode for tuning the E3 activity of an RBR-type E3 ligase.

Our careful structural analyses of the identified STK4-mediated phosphorylation sites of HOIP within the structure of the HOIP RING2-LDD/Ub complex (PDB ID: 4LJP) revealed that only the HOIP T891 site, located in the HOIP RING2 module, is adjacent to the donor Ub, while the other identified HOIP phosphorylation sites (such as HOIP T955 and S1066) are far from either the donor or acceptor Ub (Supplementary Fig. S29a). This suggests that phosphorylation at these HOIP sites is unlikely to directly interfere with HOIP’s interaction with donor or acceptor Ub. Furthermore, even though the HOIP T891 site is adjacent to the donor Ub, phosphorylation of HOIP T891 is unlikely to significantly affect the interaction of HOIP with donor Ub, as the neighboring residues of donor Ub cannot effectively interact with the phosphorylated HOIP T891 residue (Supplementary Fig. S29b). Thus, it appears that none of the STK4-mediated HOIP phosphorylation sites we identified are likely to substantially influence HOIP’s interaction with donor or acceptor Ub. Notably, both our study and a previous report^32^ showed that the S1066 residue of HOIP can be phosphorylated by STK4, although it doesn’t seem to be responsible for the inhibition of the E3 activity of HOIP. In the future, it would be interesting to know whether the phosphorylation of S1066 might regulate the potential interaction of HOIP with other unknown substrate.

In this study, we discovered that STK4 can directly bind to the RING2-LDD module of HOIP via its KD. We determined, for the first time, the crystal structure of HOIP RING2-LDD/STK4 KD complex, and elucidated the detailed binding mechanism between STK4 and HOIP. Notably, although STK4 can phosphorylate many substrates, no complex structures are currently available, likely due to the weak and dynamic interactions between STK4 and its substrates. Thus, our work also presents the first atomic picture showing how STK4 kinase specifically interacts with its substrate. Based on our structural comparison analysis, STK4 can directly compete with the E2~Ub conjugate for binding to HOIP RING2-LDD (Supplementary Fig. S17). However, our biochemical results demonstrated that the kinase-dead STK4 K59R mutant is almost unable to inhibit the E3 activity of HOIP (Fig. 3b), likely due to the much stronger binding affinity of the E2~Ub conjugate for interacting with HOIP than that of STK4 (Fig. 4c; Supplementary Fig. S4a). Therefore, the mere binding of STK4 KD to the HOIP RING2-LDD is unlikely to inhibit the E3 activity of HOIP.

Notably, in line with a previous study^44^, our biochemical results showed that STK4 (residues 1–487) binds to HOIP with a much weaker affinity than STK4 (residues 1–326) (Fig. 1d; Supplementary Fig. S4b), indicating the existence of an autoinhibitory state for full-length STK4. Then, it is puzzling how full-length STK4 could be released from this autoinhibitory state to effectively recognize HOIP. It is known that tumor necrosis factor receptor (TNFR)-associated factor 2 (TRAF2) is a subunit of the complex I, which forms upon TNFα stimulation^53^. Previous studies have demonstrated that TRAF2 can promote the homo-dimerization as well as the activation of STK4 during TNFα-induced NF-κB signaling^32,54^. Thus, TRAF2 is likely responsible for releasing STK4 from its autoinhibitory state during TNFα-induced NF-κB signaling. Nevertheless, additional studies are required to elucidate the precise mechanism underlying the regulation of the autoinhibitory state of STK4 during TNFα-induced NF-κB signaling in the future.

Finally, based on this work and previous studies^32,55^, we proposed a model to depict the inhibition of the E3 activity of HOIP by STK4-mediated phosphorylation (Fig. 4g). In this model, isolated HOIP exists in a partially auto-inhibited state, likely mediated by a potential direct interaction between its UBA and RING1 domains (Fig. 4g), which hampers the interaction of HOIP RING1 with the E2~Ub conjugate. When a Ub molecule binds to the allosteric Ub-binding site located in the RING1-IBR region of HOIP, it can somehow induce conformational changes that relieve HOIP’s auto-inhibition, thereby facilitating the binding of E2~Ub to HOIP as well as the subsequent transthiolation reaction. However, in the presence of the activated STK4, STK4 can directly associate with HOIP through the specific interaction between its KD and the RING2-LDD region of HOIP as uncovered in this study (Fig. 4g). Subsequently, the activated STK4 phosphorylates the T786 residue within the allosteric Ub-binding site of HOIP, thereby blocking the binding of Ub to the allosteric site of HOIP and eventually leading to the inhibition of the E3 activity of HOIP (Fig. 4g).

## Materials and methods

### Reagents and materials

For recombinant protein expressions in. *E. coli*, the DNA fragments encoding human STK4 and other related mutants were cloned into the pET-M3C or pET-32M3C vector (a modified version of pET-32a vector containing an N-terminal 6× His or Trx-6× His tag followed by an HRV 3C protease cutting sequence before the multiple cloning sites). We also constructed the DNA fragments encoding relevant HOIP proteins as well as the full-length HOIL-1L and Sharpin, which were cloned into pET-GST3C vector, with an N-terminal GST tag followed by an HRV 3C protease cutting sequence. Similarly, other DNA fragments encoding relevant proteins were cloned into the pET-32M3C or pET-MBP3C vector. For overexpression in HEK293T cells, the coding sequences for the full-length HOIP, HOIL-1L, Sharpin and STK4 were inserted into pFLAG-CMV-1 (Sigma) and pEGFP-C1 vectors, respectively. All the point mutations of STK4 used in this study were created using a standard PCR-based mutagenesis method, further checked by PCR screen using 2× Taq Master Mix (Vazyme Biotech Co., Ltd.) enzyme and confirmed by DNA sequencing.

### Protein expression and purification

All the proteins used in this study were expressed in *E. coli* BL21 (DE3) as N-terminal 6× His, Trx-6× His, MBP-6× His or GST fusion proteins following similar procedures in previous studies. Protein expressions in BL21 (DE3) *E. coli* were induced by 100 μM IPTG when the optical density at 600 nm reached 0.6–0.8, and further cultured at 16 °C for 16 h. For the zinc-containing proteins, 100 μM ZnCl_2_ was added to improve protein folding before IPTG induction. The bacterial cell pellets expressing 6× His-tagged proteins were re-suspended in the binding buffer (50 mM Tris, 500 mM NaCl, 5 mM imidazole at pH 7.9). GST-tagged proteins were re-suspended in the column buffer (20 mM Tris-HCl, 100 mM NaCl, and 1 mM DTT, pH 7.5). They were lysed by the ultra-high pressure homogenizer FB-110XNANO homogenizer machine (Shanghai Litu Machinery Equipment Engineering Co., Ltd.). Then, the lysate was spun down by centrifugation at 16,500 rpm (35,000× *g*) for 30 min to remove the pellet fractions. 6× His-tagged or GST-tagged proteins were affinity purified by Ni^2+^-NTA agarose or glutathione resin (GE Healthcare), respectively, and subsequently further purified by SEC (Superdex 75 or 200 26/60 column, GE Healthcare) in a suitable column buffer. If desired, the tag was cut by homemade HRV 3C protease, which specifically recognizes a cutting site in the fusion protein following the tag.

### SEC

SEC assays were carried out on an AKTA FPLC system (GE Healthcare). For each experimental group, 600 µL of STK4 protein solution, 600 µL of HOIP protein solution, and 600 µL of a mixed solution containing STK4 and HOIP proteins were prepared, which matched their concentrations in their individual protein samples. Subsequently, protein samples were loaded on to a Superdex 200 Increase 10/300 GL or Superdex 75 Increase 10/300 GL column (GE Healthcare) equilibrated with the same column buffer. Each experimental group yielded three distinct SEC profiles: one for the STK4 protein, one for the HOIP protein, and one for the mixture of the two. A forward shift or increase in the peak for the mixture sample qualitatively suggests a direct interaction between STK4 and HOIP. Relavent fractions were mixed with 2× sample loading buffer (with 100 mM DTT) and boiled at 100 °C for 10 min. Finally, the protein samples obtained from SEC assays were analyzed by SDS-PAGE. The fitting results were output to the Origin9 software and aligned with each other.

### ITC assay

ITC measurements were carried out on a MicroCal PEAQ-ITC calorimeter (Malvern) at 25 °C. All protein samples were in the same buffer containing 20 mM Tris-HCl (pH 7.5), 100 mM NaCl, and 1 mM DTT. For each ITC experiment in this study, the concentrated (~50 μM) proteins were loaded into the cell, and the other titrated proteins (~500 μM) were loaded into the syringe. The titration processes were all performed by injecting proteins from a syringe into the cell sample at time intervals of 2 min to ensure that the titration curve returned to the baseline. Meanwhile, the relevant reference control experiments, in which the protein samples in the syringe are titrated into the control buffers, were also conducted. For each ITC dataset, the reference data would be subtracted from the raw data to obtain the net result for the final fitting analysis. The titration data were analyzed using the Malvern MicroCal PEAQ-ITC analysis program and fitted using the one-site binding model.

### Analytical ultracentrifugation

Sedimentation velocity experiments were performed on a Beckman XL-I analytical ultracentrifuge equipped with an eight-cell rotor under 42,000 rpm at 20 °C. Protein samples of STK4, HOIP, and their complex or mixture were prepared, with A280 absorbance values ranging from 0.2 to 0.8 at 280 nm in a 1.0 cm path length and a volume of 400 µL for each sample. An equivalent volume (400 µL) of the corresponding protein buffer was also prepared. The partial specific volumes of different protein samples and the relevant buffer densities were calculated using the software SEDNTERP (http://www.rasmb.org/). The final sedimentation velocity data were analyzed and fitted to a continuous sedimentation coefficient distribution model using the program SEDFIT^56^. The fitting results were output to the Origin9 software and aligned with each other.

### Protein crystallization and structural elucidation

The freshly purified HOIP (residues 854–1072) protein and STK4 (residues 11–326) K59R protein were mixed at molar ratio of 1:1 and concentrated to 10 or 20 mg/mL in a buffer containing 20 mM Tris-HCl (pH 7.5), 100 mM NaCl, and 1 mM DTT. Then, 1 μL of the protein mixture was mixed with equal volumes of reservoir solution for a crystallization trial in a sitting drop manner. Crystals of the HOIP (residues 854–1072)/STK4 (residues 11–326) K59R complex were obtained in a buffer containing 0.2 M Ammonium citrate tribasic (pH 7.0), 20% w/v Polyethylene glycol 3350. Before diffraction experiments, single crystals were equilibrated with mother liquor containing 20% (v/v) glycerol as the cryo-protectant and flash-frozen in liquid nitrogen. X-ray data sets were collected at the beamline BL17U1 of the Shanghai Synchrotron Radiation Facility^57^. The diffraction data were processed and scaled using autoPROC software^58,59^. The phase problem was solved by the molecular replacement method using the modified structure of HOIP RING2-LDD (PDB ID: 4LJO) and STK4 KD (PDB ID: 3COM) as the search model with PHASER^60^. The initial structural models were rebuilt manually using COOT^61^, and then refined using PHENIX^60^. Further manual model building and adjustments were completed using COOT^61^. The qualities of the final structural models were validated by MolProbity^62^. The final refinement statistics of the determined structure in this study are listed in Supplementary Table S1. All the structural diagrams were prepared using the PyMOL program (http://www.pymol.org/).

### In vitro phosphorylation assay and dephosphorylation assays

GST-tagged HOIP and His-tagged STK4 purified from *E. coli* cells were stored in the phosphorylation buffer containing 20 mM Tris-HCl (pH 7.5), 10 mM MgCl_2_ and 2 mM DTT. Subsequently, the in vitro phosphorylation assay was conducted with 5 μM His-tagged STK4 and 100 μM relevant HOIP proteins in 100 μL of the kinase buffer containing 20 mM Tris-HCl (pH 7.5), 10 mM MgCl_2_, 10 mM ATP and 2 mM DTT for 30 min at 37 °C or indicated time at 30 °C, and after the reaction, 20 μL of reaction sample was quenched by the addition of 2× sample loading buffer (with 100 mM DTT) and boiling at 100 °C for 10 min. For Lambda Protein Phosphatase (λPP)-based dephosphorylation assay, the phosphorylation sample was mixed with a manufacturer-provided buffer (10× NEBuffer for Protein MetalloPhosphatase (PMP) and 10 mM MnCl_2_) and lambda protein phosphatase (800 U) in 50 μL of total volume for 30 min at 30 °C, and then the reaction was quenched by the addition of 2× sample loading buffer (containing 100 mM DTT) and boiled at 100 °C for 10 min. Finally, the protein samples obtained from phosphorylation and dephosphorylation assays were analyzed by SDS-PAGE or Phos-tag SDS-PAGE that uses an SDS-PAGE gel containing 50 μM Phos-tag acrylamide with 100 μM MnCl_2_ for detecting the band shift that represents phosphorylated proteins.

### Mass spectrometry and data analysis

Proteins for mass spectrometry were precipitated with acetone. The protein pellet was dried by using a Speedvac for 1–2 min. The pellet was subsequently dissolved in 8 M urea, 100 mM Tris-HCl, pH 8.5. TCEP (final concentration is 5 mM) (ThermoFisher Scientific) and iodoacetamide (final concentration: 10 mM) (Sigma) for reduction and alkylation were added to the solution and incubated at room temperature for 30 min, respectively. The protein mixture was diluted four times and digested overnight with Trypsin at 1:50 (w/w) (Promega, http://www.promega.com/). The tryptic-digested peptide solution was desalted using a MonoSpinTM C18 column (GL Science, Tokyo, Japan) and dried with a SpeedVac.

HPLC-MS/MS: Peptide samples were injected into an Easy-nLC1200 HPLC system equipped with a home-made reverse phase C18 column (75 µm*300 mm, 1.9 µm). The peptides were separated with a 120 min gradient from 4 to 100% of buffer B (buffer A: 0.1% formic acid in water; buffer B: 0.1% formic acid in 80% Acetonitrile) at 300 nL/min. The eluted peptides were ionized and directly introduced into a Q-Exactive mass spectrometer (ThermoFisher Scientific) using a nano-spray source with the application of a distal 2.5-kV spray voltage. A full mass spectrum at 70,000 resolutions (AGC target 3e6, 50 ms maximum injection time) was followed by MS/MS events with 17,500 resolutions (AGC target 5e5, 100 ms maximum injection time).

Data interpretation: The acquired MS/MS data were analyzed against an *E. coli* database (including all target proteins) using Protein Discoverer software (ThermoFisher Scientific). Mass tolerances were set at 20 ppm for precursor ions and 0.02 Da for MS/MS. Trypsin was defined as a cleavage enzyme. Cysteine alkylation by iodoacetamide was specified as a fixed modification with mass shift 57.021. The phosphorylation with a mass shift of 79.966 was set as a dynamic modification. A decoy database containing the reversed sequences of all the proteins was appended to the target database to accurately estimate peptide probabilities and false discovery rate (FDR), and FDR was set at 0.01.

### Small-angle X-ray scattering (SAXS) experiments and data analysis

SAXS experiments were performed at beamline BL19U2 of the National Facility for Protein Science Shanghai (NFPS) at Shanghai Synchrotron Radiation Facility (SSRF). Proteins for SAXS experiments were purified as above. Samples were dialyzed at 4 °C in the same container against a buffer containing 50 mM HEPES (pH 7.5), 100 mM NaCl and 2 mM DTT (SAXS buffer). The resulting dialysis buffer was reserved for buffer subtraction. For static SAXS experiments, protein concentrations were adjusted to 10 mg/mL, and serial dilutions were performed to achieve 5 mg/mL and 2 mg/mL solutions. The wavelength, λ, of X-ray radiation was set as 1.033 Å. Scattered X-ray intensities were collected using a Pilatus 2 M detector (DECTRIS Ltd). To reduce the radiation damage, a flow cell made of a cylindrical quartz capillary with a diameter of 1.5 mm and a wall of 10 µm was used. SAXS data were collected as 20 × 1 s exposures and scattering profiles for the 20 passes were compared at 10 °C using 60 μL sample in SAXS buffer. No concentration-dependent effects or radiation damage were observed in the static samples.

The 2-D scattering images were converted to 1-D SAXS curves through azimuthal averaging after solid angle correction and then normalizing with the intensity of the transmitted X-ray beam, using the software package BioXTAS RAW^63^. The ab initio shapes were determined using DAMMIF^64^ with 10 DAMMIF runs for each experimental group (5 mg/mL), and DAMAVER^65^ was used to analyze the normalized spatial discrepancy (NSD) between the 10 models.

### In vitro ubiquitination assay

Recombinant murine UBA1 (E1), human UBE2L3 (E2) and Ub expressed in *E. coli* cells were used for the assay. The reaction systems used for the in vitro ubiquitination assays of HOIP containing 100 μM Ub, 1 μM UBA1, 2 μM UBE2L3 and 0.5 μM HOIP or its mutants in 20 mM Tris-HCl (pH 7.5), 100 mM NaCl, 1 mM DTT, 10 mM ATP and 10 mM MgCl_2_. Reaction mixtures were incubated at 37 °C for the desired time, and aliquots of the sample were immediately denatured by mixing with 2× sample loading buffer (with 100 mM DTT). For the in vitro ubiquitination assay that uses phosphorylated HOIP, 4 μM 6× His-tagged full-length STK4 protein was used to phosphorate 80 μM WT HOIP or 80 μM HOIP T786E, and was further removed by Ni^2+^-NTA agarose. For the in vitro ubiquitination assay that STK4 inhibits the E3 activity of LUBAC, LUBAC pre-mixed with STK4 or its mutants in the phosphorylation buffer with extra 10 mM ATP at 30 °C for 30 min, and then 100 μM Ub, 1 μM UBA1, and 2 μM UBE2L3 were added into the phosphorylation system. Reaction mixtures were incubated at 37 °C, and samples were taken at time points as indicated and quenched by adding the 2× sample loading buffer (without DTT).

### E2~Ub discharge assay

The E2 UBE2L3 was charged with Ub by incubating 20 μM UBE2L3 with 200 nM UBE1, 50 μM Ub, 10 mM ATP, and 10 mM MgCl_2_ in a PBS buffer (pH 7.5) at 37 °C for 30 min. The reaction was depleted of ATP by incubating with 2 U of Apyrase (Sigma, A6410) per 100-μL reaction at room temperature for 5 min. For the time course UBE2L3-Ub discharge assays, the charged UBE2L3-Ub conjugate was mixed with HOIP (residues 697–1072) or its mutants for a final concentration of 0.5 μM as well as HOIP (residues 480–1072) or its mutants for a final concentration of 3 μM. In the meantime, the sample proteins obtained from the phosphorylation assay were mixed with equal volumes of the charged UBE2L3-Ub conjugate, and then reaction samples were taken at time points as indicated and quenched by adding the 2× sample loading buffer (without DTT).

### Cell culture, transfection and co-immunoprecipitation assay

HEK293T cells were cultured in Dulbecco’s modified Eagle’s medium (DMEM, Invitrogen) supplemented with 10% fetal bovine serum (FBS, Invitrogen). Co-transfections of FLAG-HOIP and GFP-STK4 or related mutant plasmids were performed with Lipofectamine 2000 (ThermoFisher Scientific) according to the manufacturer’s instructions. After 24 h, HEK293T cells transiently expressing proteins were harvested, washed with PBS buffer, and lysed for 40 min at 4 °C in a lysis buffer containing 50 mM Tris-HCl (pH 7.5), 150 mM NaCl, 0.5% NP-40, 1 mM PMSF and 1% protease inhibitor cocktail (AMRESCO). Lysates were centrifuged, and then supernatants were incubated with anti-GFP monoclonal antibody-agarose (Medical & Biological Laboratories) for 1 h at 4 °C. Precipitated proteins were washed with lysis buffer 6 times, and collected by brief centrifugation. Then, the beads were resuspended with the 2 × sample loading buffer (containing 100 mM DTT) and boiled for 10 min at 100 °C. Subsequently, the precipitated proteins were resolved in SDS-PAGE gel, and the GFP-tagged STK4 and FLAG-tagged HOIP were detected by western blot using GFP antibody (1:2000 dilution; Proteintech, 50430-2-AP) and FLAG antibody (1:1000 dilution; Proteintech, 20543-1-AP), respectively.

### Luciferase assay

HEK293T cells were cultured in 12-well plate to a confluency of ~50% in DMEM media supplemented 10% (v/v) fetal bovine serum and 1% penicillin-streptomycin before transfection. Each well was transfected with 200 ng pGL-SV40, 20 ng pRL-NF-κB, 50 ng FLAG-Sharpin, 50 ng FLAG-HOIL-1L, and 500 ng FLAG-HOIP by lipofectamine 2000 (Thermofisher Scientific) according to the manufacturer’s protocol. After 24 h, cells were washed twice with cold PBS and collected for Dual Luciferase Reporter Assay Kit (Vazyme) according to the manufacturer’s protocol.

### In vivo linear ubiquitination assay

HeLa cells were lysed in 1× sample loading buffer (containing 50 mM DTT) for 10 min at 100 °C after the transfection for 24 h using the expression vectors for FLAG-HOIP, FLAG-HOIL-1L and FLAG-Sharpin. An overnight wet transfer was used to transfer linear Ub chain onto methanol-activated PVDF membranes (Millipore). The samples were detected by western blot using specific FLAG antibody (1:2000 dilution; Proteintech, 20543-1-AP), linear Ub antibody (1:1000 dilution; Millipore, MABS199) and β-actin antibody (1:20,000 dilution; Proteintech, 66009-1-Ig), respectively.

## Supplementary information

The related PDB validation report for the STK4/HOIP complex structure

The Supplemental Figures and Table

## Data Availability

The atomic coordinate and structure factor of the crystal structure of the HOIP RING2-LDD/STK4 KD complex mentioned in this study have been deposited in the Protein Data Bank under the accession code 9IIC. All additional experimental data are available from the corresponding author on request.

## References

[1] Weissman, A. M. Themes and variations on ubiquitylation. *Nat. Rev. Mol. Cell Biol.* **2**, 169–178 (2001). 11265246 10.1038/35056563

[2] Pickart, C. M. & Eddins, M. J. Ubiquitin: structures, functions, mechanisms. *Biochim. Biophys. Acta* **1695**, 55–72 (2004). 15571809 10.1016/j.bbamcr.2004.09.019

[3] Kerscher, O., Felberbaum, R. & Hochstrasser, M. Modification of proteins by ubiquitin and ubiquitin-like proteins. *Annu. Rev. Cell Dev. Biol.* **22**, 159–180 (2006). 16753028 10.1146/annurev.cellbio.22.010605.093503

[4] Hicke, L., Schubert, H. L. & Hill, C. P. Ubiquitin-binding domains. *Nat. Rev. Mol. Cell Biol.* **6**, 610–621 (2005). 16064137 10.1038/nrm1701

[5] Popovic, D., Vucic, D. & Dikic, I. Ubiquitination in disease pathogenesis and treatment. *Nat. Med.* **20**, 1242–1253 (2014). 25375928 10.1038/nm.3739

[6] Shaid, S., Brandts, C. H., Serve, H. & Dikic, I. Ubiquitination and selective autophagy. *Cell Death Differ.* **20**, 21–30 (2013). 22722335 10.1038/cdd.2012.72 PMC3524631

[7] Hershko, A. & Ciechanover, A. The ubiquitin system. *Annu. Rev. Biochem.* **67**, 425–479 (1998). 9759494 10.1146/annurev.biochem.67.1.425

[8] Heride, C., Urbe, S. & Clague, M. J. Ubiquitin code assembly and disassembly. *Curr. Biol.* **24**, R215–R220 (2014). 24650902 10.1016/j.cub.2014.02.002

[9] Akutsu, M., Dikic, I. & Bremm, A. Ubiquitin chain diversity at a glance. *J. Cell Sci.* **129**, 875–880 (2016). 26906419 10.1242/jcs.183954

[10] Komander, D. & Rape, M. The ubiquitin code. *Annu. Rev. Biochem.* **81**, 203–229 (2012). 22524316 10.1146/annurev-biochem-060310-170328

[11] Kirisako, T. et al. A ubiquitin ligase complex assembles linear polyubiquitin chains. *EMBO J.* **25**, 4877–4887 (2006). 17006537 10.1038/sj.emboj.7601360 PMC1618115

[12] Rieser, E., Cordier, S. M. & Walczak, H. Linear ubiquitination: a newly discovered regulator of cell signalling. *Trends Biochem. Sci.* **38**, 94–102 (2013). 23333406 10.1016/j.tibs.2012.11.007

[13] Tokunaga, F. et al. SHARPIN is a component of the NF-kappa B-activating linear ubiquitin chain assembly complex. *Nature* **471**, 633–636 (2011). 21455180 10.1038/nature09815

[14] Ikeda, F. et al. SHARPIN forms a linear ubiquitin ligase complex regulating NF-kappa B activity and apoptosis. *Nature* **471**, 637–641 (2011). 21455181 10.1038/nature09814 PMC3085511

[15] Gerlach, B. et al. Linear ubiquitination prevents inflammation and regulates immune signalling. *Nature* **471**, 591–596 (2011). 21455173 10.1038/nature09816

[16] Iwai, K., Fujita, H. & Sasaki, Y. Linear ubiquitin chains: NF-kappa B signalling, cell death and beyond. *Nat. Rev. Mol. Cell Biol.* **15**, 503–508 (2014). 25027653 10.1038/nrm3836

[17] Liu, J. & Pan, L. Structural bases of the assembly, recognition and disassembly of linear ubiquitin chain. *Biochim. Biophy. Acta Mol. Cell Res.* **1865**, 1410–1422 (2018). 10.1016/j.bbamcr.2018.07.003 29981772

[18] Haas, T. L. et al. Recruitment of the linear ubiquitin chain assembly complex stabilizes the TNF-R1 signaling complex and is required for TNF-mediated gene induction. *Mol. Cell* **36**, 831–844 (2009). 20005846 10.1016/j.molcel.2009.10.013

[19] Tokunaga, F. et al. Involvement of linear polyubiquitylation of NEMO in NF-kappa B activation. *Nat. Cell Biol.* **11**, 123–132 (2009). 19136968 10.1038/ncb1821

[20] Inn, K. S. et al. Linear ubiquitin assembly complex negatively regulates RIG-I- and TRIM25-mediated type I interferon induction. *Mol. Cell* **41**, 354–365 (2011). 10.1016/j.molcel.2010.12.029 PMC3070481 21292167

[21] Damgaard, R. B. et al. The ubiquitin ligase XIAP recruits LUBAC for NOD2 signaling in inflammation and innate immunity. *Mol. Cell* **46**, 746–758 (2012). 22607974 10.1016/j.molcel.2012.04.014

[22] Rivkin, E. et al. The linear ubiquitin-specific deubiquitinase gumby regulates angiogenesis. *Nature* **498**, 318–324 (2013). 23708998 10.1038/nature12296 PMC4931916

[23] Li, F. X. et al. Structural insights into the ubiquitin recognition by OPTN (optineurin) and its regulation by TBK1-mediated phosphorylation. *Autophagy* **14**, 66–79 (2018). 29394115 10.1080/15548627.2017.1391970 PMC5846504

[24] van Wijk, S. J. L. et al. Linear ubiquitination of cytosolic *Salmonella Typhimurium* activates NF-kappaB and restricts bacterial proliferation. *Nat. Microbiol.* **2**, 17066 (2017). 28481361 10.1038/nmicrobiol.2017.66

[25] Gijbels, M. J. J. et al. Pathogenesis of skin lesions in mice with chronic proliferative dermatitis (cpdm/cpdm). *Am. J. Pathol.* **148**, 941–950 (1996). 8774148 PMC1861706

[26] Peltzer, N. et al. HOIP deficiency causes embryonic lethality by aberrant TNFR1-mediated endothelial cell death. *Cell Rep.* **9**, 153–165 (2014). 25284787 10.1016/j.celrep.2014.08.066

[27] Sasaki, Y. et al. Defective immune responses in mice lacking LUBAC-mediated linear ubiquitination in B cells. *EMBO J.* **32**, 2463–2476 (2013). 23942237 10.1038/emboj.2013.184 PMC3770953

[28] Boisson, B. et al. Immunodeficiency, autoinflammation and amylopectinosis in humans with inherited HOIL-1 and LUBAC deficiency. *Nat. Immunol.* **13**, 1178–1186 (2012). 23104095 10.1038/ni.2457 PMC3514453

[29] Verhelst, K. et al. A20 inhibits LUBAC-mediated NF-kappaB activation by binding linear polyubiquitin chains via its zinc finger 7. *EMBO J.* **31**, 3845–3855 (2012). 23032186 10.1038/emboj.2012.240 PMC3463847

[30] Keusekotten, K. et al. OTULIN antagonizes LUBAC signaling by specifically hydrolyzing Met1-linked polyubiquitin. *Cell* **153**, 1312–1326 (2013). 23746843 10.1016/j.cell.2013.05.014 PMC3690481

[31] Takiuchi, T. et al. Suppression of LUBAC-mediated linear ubiquitination by a specific interaction between LUBAC and the deubiquitinases CYLD and OTULIN. *Genes Cells* **19**, 254–272 (2014). 24461064 10.1111/gtc.12128

[32] Lee, I. Y. et al. MST1 negatively regulates TNFα-induced NF-κB signaling through modulating LUBAC activity. *Mol. Cell* **73**, 1138–1349.e6 (2019). 30901564 10.1016/j.molcel.2019.01.022

[33] Stieglitz, B. et al. Structural basis for ligase-specific conjugation of linear ubiquitin chains by HOIP. *Nature* **503**, 422–426 (2013). 24141947 10.1038/nature12638 PMC3838313

[34] Lechtenberg, B. C. et al. Structure of a HOIP/E2~ubiquitin complex reveals RBR E3 ligase mechanism and regulation. *Nature* **529**, 546–550 (2016). 26789245 10.1038/nature16511 PMC4856479

[35] Kumar, A. et al. Parkin-phosphoubiquitin complex reveals cryptic ubiquitin-binding site required for RBR ligase activity. *Nat. Struct. Mol. Biol.* **24**, 475–483 (2017). 28414322 10.1038/nsmb.3400 PMC5420311

[36] Xu, X. L. et al. Mechanistic insights into the enzymatic activity of E3 ligase HOIL-1L and its regulation by the linear ubiquitin chain binding. *Sci. Adv.* **9**, eadi4599 (2023). 37831767 10.1126/sciadv.adi4599 PMC10575588

[37] Wauer, T., Simicek, M., Schubert, A. & Komander, D. Mechanism of phospho-ubiquitin-induced PARKIN activation. *Nature* **524**, 370–374 (2015). 26161729 10.1038/nature14879 PMC4984986

[38] Kelsall, I. R. et al. HOIL-1 ubiquitin ligase activity targets unbranched glucosaccharides and is required to prevent polyglucosan accumulation. *EMBO J.* **41**, e109700 (2022). 35274759 10.15252/embj.2021109700 PMC9016349

[39] Manning, G., Whyte, D. B., Martinez, R., Hunter, T. & Sudarsanam, S. The protein kinase complement of the human genome. *Science* **298**, 1912–1934 (2002). 12471243 10.1126/science.1075762

[40] Ubersax, J. A. & Ferrell, J. E. Jr Mechanisms of specificity in protein phosphorylation. *Nat. Rev. Mol. Cell Biol.* **8**, 530–541 (2007). 17585314 10.1038/nrm2203

[41] Galan, J. A. & Avruch, J. MST1/MST2 protein kinases: regulation and physiologic roles. *Biochemistry* **55**, 5507–5519 (2016). 27618557 10.1021/acs.biochem.6b00763 PMC5479320

[42] Ma, S., Meng, Z., Chen, R. & Guan, K. L. The Hippo pathway: biology and pathophysiology. *Annu. Rev. Biochem.* **88**, 577–604 (2019). 30566373 10.1146/annurev-biochem-013118-111829

[43] Khokhlatchev, A. et al. Identification of a novel Ras-regulated proapoptotic pathway. *Curr. Biol.* **12**, 253–265 (2002). 11864565 10.1016/s0960-9822(02)00683-8

[44] Creasy, C. L., Ambrose, D. M. & Chernoff, J. The Ste20-like protein kinase, Mst1, dimerizes and contains an inhibitory domain. *J. Biol. Chem.* **271**, 21049–21053 (1996). 8702870 10.1074/jbc.271.35.21049

[45] Cheung, W. L. et al. Apoptotic phosphorylation of histone H2B is mediated by mammalian sterile twenty kinase. *Cell* **113**, 507–517 (2003). 12757711 10.1016/s0092-8674(03)00355-6

[46] Lehtinen, M. K. et al. A conserved MST-FOXO signaling pathway mediates oxidative-stress responses and extends life span. *Cell* **125**, 987–1001 (2006). 16751106 10.1016/j.cell.2006.03.046

[47] Praskova, M., Xia, F. & Avruch, J. MOBKL1A/MOBKL1B phosphorylation by MST1 and MST2 inhibits cell proliferation. *Curr. Biol.* **18**, 311–321 (2008). 18328708 10.1016/j.cub.2008.02.006 PMC4682548

[48] Chan, E. H. et al. The Ste20-like kinase Mst2 activates the human large tumor suppressor kinase Lats1. *Oncogene* **24**, 2076–2086 (2005). 15688006 10.1038/sj.onc.1208445

[49] Praskova, M., Khoklatchev, A., Ortiz-Vega, S. & Avruch, J. Regulation of the MST1 kinase by autophosphorylation, by the growth inhibitory proteins, RASSF1 and NORE1, and by Ras. *Biochem. J.* **381**, 453–462 (2004). 15109305 10.1042/BJ20040025 PMC1133852

[50] Fan, F. et al. Pharmacological targeting of kinases MST1 and MST2 augments tissue repair and regeneration. *Sci. Transl. Med.* **8**, 352ra108 (2016). 27535619 10.1126/scitranslmed.aaf2304

[51] Abramson, J. et al. Accurate structure prediction of biomolecular interactions with AlphaFold 3. *Nature* **630**, 493–500 (2024). 38718835 10.1038/s41586-024-07487-w PMC11168924

[52] Cotton, T. R. & Lechtenberg, B. C. Chain reactions: molecular mechanisms of RBR ubiquitin ligases. *Biochem. Soc. Trans.* **48**, 1737–1750 (2020). 32677670 10.1042/BST20200237 PMC7458406

[53] Micheau, O. & Tschopp, J. Induction of TNF receptor I-mediated apoptosis via two sequential signaling complexes. *Cell* **114**, 181–190 (2003). 12887920 10.1016/s0092-8674(03)00521-x

[54] Roh, K. H. & Choi, E. J. TRAF2 functions as an activator switch in the reactive oxygen species-induced stimulation of MST1. *Free Radic. Biol. Med.* **91**, 105–113 (2016). 26698664 10.1016/j.freeradbiomed.2015.12.010

[55] Liu, J. P. et al. Structural insights into SHARPIN-mediated activation of HOIP for the linear ubiquitin chain assembly. *Cell Rep.* **21**, 27–36 (2017). 28978479 10.1016/j.celrep.2017.09.031

[56] Schuck, P. Size-distribution analysis of macromolecules by sedimentation velocity ultracentrifugation and lamm equation modeling. *Biophy. J.* **78**, 1606–1619 (2000). 10.1016/S0006-3495(00)76713-0 PMC1300758 10692345

[57] Wang, Z. et al. Automatic crystal centring procedure at the SSRF macromolecular crystallography beamline. *J. Synchrotron Radiat.* **23**, 1323–1332 (2016). 27787238 10.1107/S160057751601451X

[58] Vonrhein, C. et al. Data processing and analysis with the autoPROC toolbox. *Acta Crystallogr. D Biol. Crystallogr.* **67**, 293–302 (2011). 10.1107/S0907444911007773 PMC3069744 21460447

[59] Kabsch, W. X. D. S. *Acta Crystallogr. D Biol. Crystallogr.* **66**, 125–132 (2010). 10.1107/S0907444909047337 PMC2815665 20124692

[60] Adams, P. D. et al. PHENIX: building new software for automated crystallographic structure determination. *Acta Crystallogr. D Biol. Crystallogr.* **58**, 1948–1954 (2002). 10.1107/s0907444902016657 12393927

[61] Emsley, P. & Cowtan, K. Coot: model-building tools for molecular graphics. *Acta Crystallogr. D Biol. Crystallogr.* **60**, 2126–2132 (2004). 10.1107/S0907444904019158 15572765

[62] Davis, I. W. et al. MolProbity: all-atom contacts and structure validation for proteins and nucleic acids. *Nucleic Acids Res.* **35**, W375–W383 (2007). 17452350 10.1093/nar/gkm216 PMC1933162

[63] Nielsen, S. S. et al. BioXTAS RAW, a software program for high-throughput automated small-angle X-ray scattering data reduction and preliminary analysis. *J. Appl. Crystallogr.* **42**, 959–964 (2009).

[64] Franke, D. & Svergun, D. I. DAMMIF, a program for rapid ab-initio shape determination in small-angle scattering. *J. Appl. Crystallogr.* **42**, 342–346 (2009). 27630371 10.1107/S0021889809000338 PMC5023043

[65] Volkov, V. V. & Svergun, D. I. Uniqueness of ab initio shape determination in small-angle scattering. *J. Appl. Crystallogr.* **36**, 860–864 (2003). 10.1107/S0021889809000338 PMC5023043 27630371

