# Supplementary material for: STK4 inhibits the E3 activity of HOIP by phosphorylating its allosteric ubiquitin-binding site
Source: Cell Discov. 2025 Sep 16;11:75. doi: 10.1038/s41421-025-00824-x (PMC12441119; doi:10.1038/s41421-025-00824-x)
Supplement: Supplementary file 1 — The related PDB validation report for the STK4/HOIP complex structure [file 41421_2025_824_MOESM1_ESM.pdf]

Full wwPDB X-ray Structure Validation Report (i)

Jun 24, 2024 – 04:48 PM JST

PDB ID	:	9IIC
Title	:	Crystal structure of HOIP RING2-LDD in complex with STK4 KD domain
Deposited on	:	2024-06-20
Resolution	:	2.78 Å(reported)

This wwPDB validation report is for manuscript review

For Manuscript Review

This is a Full wwPDB X-ray Structure Validation Report.

This report is produced by the wwPDB biocuration pipeline after annotation of the structure.

We welcome your comments at *validation@mail.wwpdb.org*
A user guide is available at
https://www.wwpdb.org/validation/2017/XrayValidationReportHelp
with specific help available everywhere you see the (i) symbol.

The types of validation reports are described at
http://www.wwpdb.org/validation/2017/FAQs#types.

The following versions of software and data (see references (i)) were used in the production of this report:

MolProbity	:	4.02b-467
Mogul	:	1.8.5 (274361), CSD as541be (2020)
Xtriage (Phenix)	:	1.13
EDS	:	2.37.1
buster-report	:	1.1.7 (2018)
Percentile statistics	:	20191225.v01 (using entries in the PDB archive December 25th 2019)
Refmac	:	5.8.0158
CCP4	:	7.0.044 (Gargrove)
Ideal geometry (proteins)	:	Engh & Huber (2001)
Ideal geometry (DNA, RNA)	:	Parkinson et al. (1996)

1 Overall quality at a glance (i)

The following experimental techniques were used to determine the structure:
X-RAY DIFFRACTION

The reported resolution of this entry is 2.78 Å.

Percentile scores (ranging between 0-100) for global validation metrics of the entry are shown in the following graphic. The table shows the number of entries on which the scores are based.

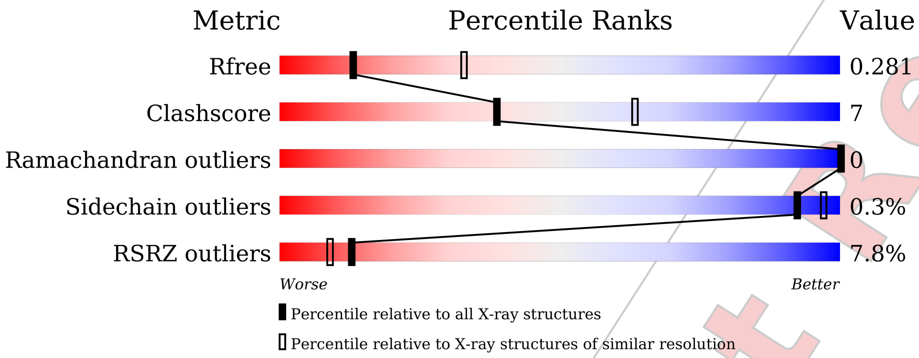

Metric	Whole archive (#Entries)	Similar resolution (#Entries, resolution range(Å))
R_{free}	130704	4107 (2.80-2.76)
Clashscore	141614	4575 (2.80-2.76)
Ramachandran outliers	138981	4487 (2.80-2.76)
Sidechain outliers	138945	4489 (2.80-2.76)
RSRZ outliers	127900	4027 (2.80-2.76)

The table below summarises the geometric issues observed across the polymeric chains and their fit to the electron density. The red, orange, yellow and green segments of the lower bar indicate the fraction of residues that contain outliers for >=3, 2, 1 and 0 types of geometric quality criteria respectively. A grey segment represents the fraction of residues that are not modelled. The numeric value for each fraction is indicated below the corresponding segment, with a dot representing fractions <=5% The upper red bar (where present) indicates the fraction of residues that have poor fit to the electron density. The numeric value is given above the bar.

Mol	Chain	Length	Quality of chain
1	A	302	2% / 78% 17% 5%
1	B	302	2% / 82% 14% •
2	C	215	14% / 81% 19%
2	D	215	17% / 84% 16%

Validation Pipeline (wwPDB-VP) : 2.37.1

For Manuscript Review

2 Entry composition i

There are 5 unique types of molecules in this entry. The entry contains 8009 atoms, of which 0 are hydrogens and 0 are deuteriums.

In the tables below, the ZeroOcc column contains the number of atoms modelled with zero occupancy, the AltConf column contains the number of residues with at least one atom in alternate conformation and the Trace column contains the number of residues modelled with at most 2 atoms.

- Molecule 1 is a protein called Serine/threonine-protein kinase 4 37kDa subunit.

Mol	Chain	Residues	Atoms	ZeroOcc	AltConf	Trace
1	B	290	Total C N O S<br>2317 1477 395 433 12	0	0	0
1	A	287	Total C N O S<br>2284 1458 388 426 12	0	0	0

There are 4 discrepancies between the modelled and reference sequences:

Chain	Residue	Modelled	Actual	Comment	Reference
B	10	PHE	-	expression tag	UNP Q13043
B	59	ARG	LYS	engineered mutation	UNP Q13043
A	10	PHE	-	expression tag	UNP Q13043
A	59	ARG	LYS	engineered mutation	UNP Q13043

- Molecule 2 is a protein called E3 ubiquitin-protein ligase RNF31.

Mol	Chain	Residues	Atoms	ZeroOcc	AltConf	Trace
2	C	215	Total C N O S<br>1691 1059 311 305 16	0	0	0
2	D	215	Total C N O S<br>1691 1059 311 305 16	0	0	0

- Molecule 3 is GLYCEROL (three-letter code: GOL) (formula: $C_3H_8O_3$) (labeled as "Ligand of Interest" by depositor).

For Manuscript Review

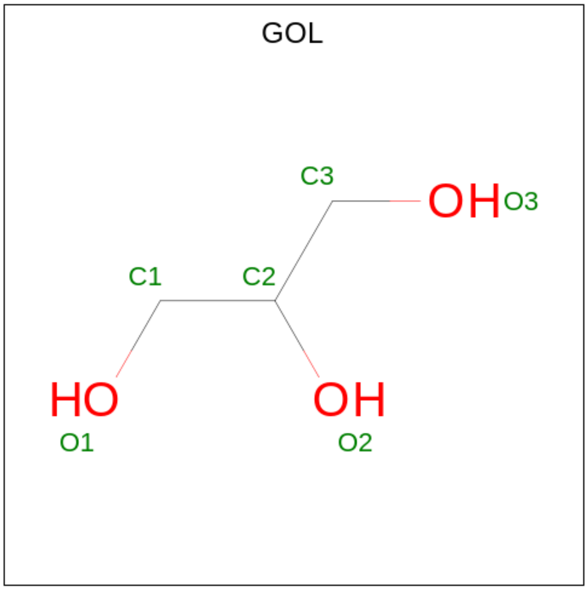

Mol	Chain	Residues	Atoms	ZeroOcc	AltConf
3	B	1	Total C O<br>6 3 3	0	0
3	A	1	Total C O<br>6 3 3	0	0

- Molecule 4 is ZINC ION (three-letter code: ZN) (formula: Zn) (labeled as "Ligand of Interest" by depositor).

Mol	Chain	Residues	Atoms	ZeroOcc	AltConf
4	C	4	Total Zn<br>4 4	0	0
4	D	4	Total Zn<br>4 4	0	0

- Molecule 5 is water.

Mol	Chain	Residues	Atoms	ZeroOcc	AltConf
5	B	3	Total O<br>3 3	0	0
5	C	1	Total O<br>1 1	0	0
5	A	2	Total O<br>2 2	0	0

For Manuscript Review

3 Residue-property plots (i)

These plots are drawn for all protein, RNA, DNA and oligosaccharide chains in the entry. The first graphic for a chain summarises the proportions of the various outlier classes displayed in the second graphic. The second graphic shows the sequence view annotated by issues in geometry and electron density. Residues are color-coded according to the number of geometric quality criteria for which they contain at least one outlier: green = 0, yellow = 1, orange = 2 and red = 3 or more. A red dot above a residue indicates a poor fit to the electron density (RSRZ > 2). Stretches of 2 or more consecutive residues without any outlier are shown as a green connector. Residues present in the sample, but not in the model, are shown in grey.

• Molecule 1: Serine/threonine-protein kinase 4 37kDa subunit

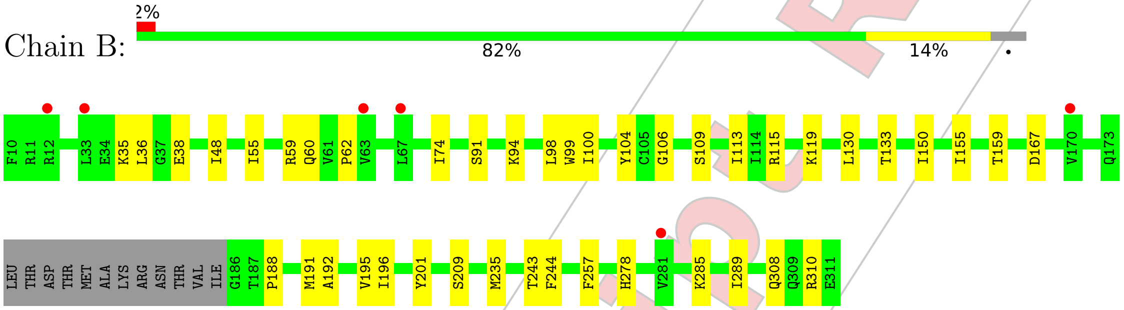

• Molecule 1: Serine/threonine-protein kinase 4 37kDa subunit

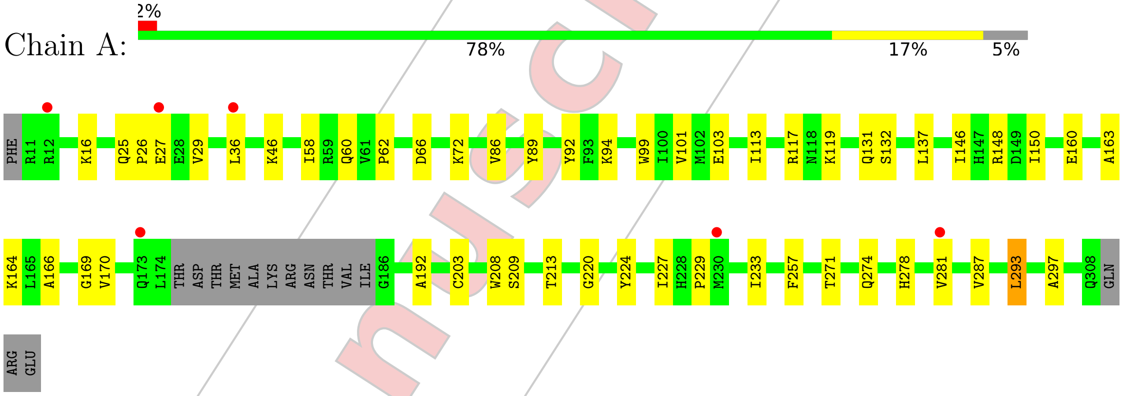

• Molecule 2: E3 ubiquitin-protein ligase RNF31

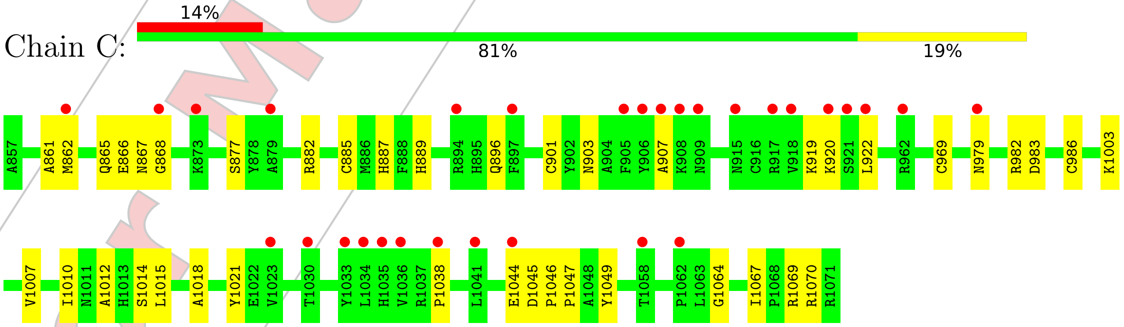

• Molecule 2: E3 ubiquitin-protein ligase RNF31

For Manuscript Review

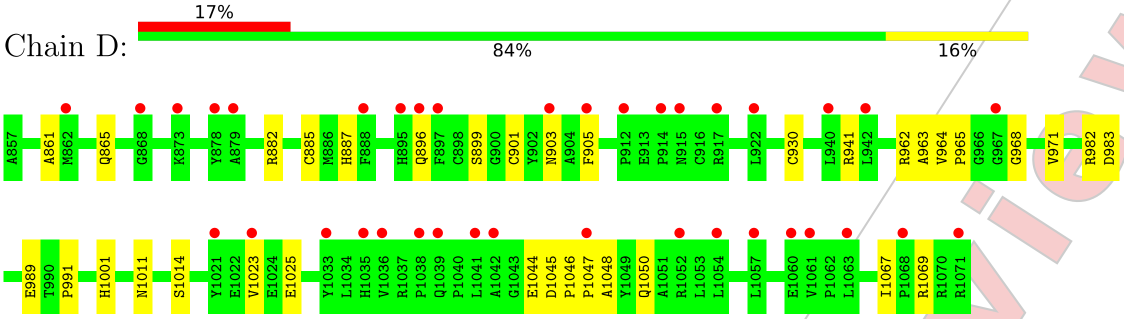

For Manuscript Review

4 Data and refinement statistics (i)

Property	Value	Source
Space group	C 1 2 1	Depositor
Cell constants<br>a, b, c, α, β, γ	138.99Å 96.36Å 115.77Å<br>90.00° 118.82° 90.00°	Depositor
Resolution (Å)	68.87 – 2.78<br>75.57 – 2.78	Depositor<br>EDS
% Data completeness<br>(in resolution range)	97.0 (68.87-2.78)<br>97.5 (75.57-2.78)	Depositor<br>EDS
R_{merge}	0.08	Depositor
R_{sym}	(Not available)	Depositor
$< I/\sigma(I) >$ [1]	2.88 (at 2.77Å)	Xtriage
Refinement program	PHENIX (1.20.1_4487: ???)	Depositor
R, R_{free}	0.237 , 0.280<br>0.240 , 0.281	Depositor<br>DCC
R_{free} test set	1654 reflections (5.01%)	wwPDB-VP
Wilson B-factor (Å^2)	71.1	Xtriage
Anisotropy	0.705	Xtriage
Bulk solvent k_{sol}(e/Å^3), B_{sol}(Å^2)	0.31 , 73.1	EDS
L-test for twinning[2]	$< \|L\| > = 0.49$, $< L^2 > = 0.32$	Xtriage
Estimated twinning fraction	No twinning to report.	Xtriage
F_o,F_c correlation	0.93	EDS
Total number of atoms	8009	wwPDB-VP
Average B, all atoms (Å^2)	113.0	wwPDB-VP

Xtriage's analysis on translational NCS is as follows: *The largest off-origin peak in the Patterson function is 7.72% of the height of the origin peak. No significant pseudotranslation is detected.*

[1] Intensities estimated from amplitudes.

[2] Theoretical values of $< |L| >$, $< L^2 >$ for acentric reflections are 0.5, 0.333 respectively for untwinned datasets, and 0.375, 0.2 for perfectly twinned datasets.

For Manuscript Review

5 Model quality (i)

5.1 Standard geometry (i)

Bond lengths and bond angles in the following residue types are not validated in this section: GOL, ZN

The Z score for a bond length (or angle) is the number of standard deviations the observed value is removed from the expected value. A bond length (or angle) with $|Z| > 5$ is considered an outlier worth inspection. RMSZ is the root-mean-square of all Z scores of the bond lengths (or angles).

Mol	Chain	Bond lengths		Bond angles	
		RMSZ	#$\|Z\|$ >5	RMSZ	#$\|Z\|$ >5
1	A	0.27	0/2331	0.51	1/3152 (0.0%)
1	B	0.25	0/2365	0.48	0/3195
2	C	0.26	0/1734	0.53	0/2348
2	D	0.26	0/1734	0.53	0/2348
All	All	0.26	0/8164	0.51	1/11043 (0.0%)

There are no bond length outliers.

All (1) bond angle outliers are listed below:

Mol	Chain	Res	Type	Atoms	Z	Observed(^{o})	Ideal(^{o})
1	A	293	LEU	CA-CB-CG	5.44	127.81	115.30

There are no chirality outliers.

There are no planarity outliers.

5.2 Too-close contacts (i)

In the following table, the Non-H and H(model) columns list the number of non-hydrogen atoms and hydrogen atoms in the chain respectively. The H(added) column lists the number of hydrogen atoms added and optimized by MolProbity. The Clashes column lists the number of clashes within the asymmetric unit, whereas Symm-Clashes lists symmetry-related clashes.

Mol	Chain	Non-H	H(model)	H(added)	Clashes	Symm-Clashes
1	A	2284	0	2301	42	0
1	B	2317	0	2326	25	0
2	C	1691	0	1637	25	0
2	D	1691	0	1637	29	0
3	A	6	0	8	0	0
3	B	6	0	8	0	0

Continued on next page...

For Manuscript Review

Continued from previous page...

Mol	Chain	Non-H	H(model)	H(added)	Clashes	Symm-Clashes
4	C	4	0	0	0	0
4	D	4	0	0	0	0
5	A	2	0	0	0	0
5	B	3	0	0	0	0
5	C	1	0	0	0	0
All	All	8009	0	7917	119	0

The all-atom clashscore is defined as the number of clashes found per 1000 atoms (including hydrogen atoms). The all-atom clashscore for this structure is 7.

All (119) close contacts within the same asymmetric unit are listed below, sorted by their clash magnitude.

Atom-1	Atom-2	Interatomic distance (Å)	Clash overlap (Å)
1:A:224:TYR:HD1	1:A:227:ILE:HD12	1.04	1.14
2:D:964:VAL:CG1	2:D:968:GLY:CA	2.25	1.13
2:D:964:VAL:HG13	2:D:968:GLY:CA	1.78	1.11
2:D:964:VAL:HG13	2:D:968:GLY:HA2	1.34	1.09
1:A:224:TYR:CD1	1:A:227:ILE:HD12	1.90	1.06
2:D:964:VAL:CG1	2:D:968:GLY:HA3	1.84	1.05
2:D:964:VAL:CG1	2:D:968:GLY:HA2	1.86	1.02
1:A:72:LYS:HG2	1:A:170:VAL:HG11	1.54	0.90
1:A:224:TYR:HD1	1:A:227:ILE:CD1	1.84	0.88
2:C:1038:PRO:O	2:C:1049:TYR:OH	1.96	0.83
2:D:964:VAL:HG11	2:D:968:GLY:HA3	1.65	0.77
1:A:72:LYS:HE2	1:A:170:VAL:CG1	2.15	0.76
1:A:224:TYR:CD1	1:A:227:ILE:CD1	2.65	0.73
1:A:72:LYS:HE2	1:A:170:VAL:HG13	1.71	0.71
2:D:964:VAL:HG12	2:D:968:GLY:CA	2.23	0.68
2:D:964:VAL:HG12	2:D:968:GLY:HA2	1.75	0.68
1:A:131:GLN:HG3	1:A:281:VAL:CG1	2.25	0.67
1:B:150:ILE:HB	1:B:209:SER:OG	1.95	0.66
1:A:72:LYS:CG	1:A:170:VAL:HG11	2.26	0.64
2:D:962:ARG:HG2	2:D:963:ALA:H	1.61	0.64
2:D:901:CYS:SG	2:D:903:ASN:ND2	2.73	0.62
1:A:131:GLN:HG3	1:A:281:VAL:HG12	1.83	0.60
1:B:308:GLN:O	1:B:310:ARG:NH1	2.36	0.58
2:C:1014:SER:HA	2:C:1069:ARG:HH21	1.68	0.58
1:A:72:LYS:CE	1:A:170:VAL:CG1	2.82	0.57
2:C:1012:ALA:HB2	2:C:1070:ARG:HH21	1.70	0.57
2:C:885:CYS:SG	2:C:887:HIS:NE2	2.79	0.56
1:A:271:THR:HG23	1:A:274:GLN:H	1.69	0.56

Continued on next page...

For Manuscript Review

Continued from previous page...

Atom-1	Atom-2	Interatomic distance (Å)	Clash overlap (Å)
1:B:195:VAL:HG23	1:B:201:TYR:CD1	2.41	0.56
1:A:209:SER:O	1:A:213:THR:HG23	2.05	0.55
2:C:867:ASN:OD1	2:C:1070:ARG:HD3	2.07	0.54
1:A:137:LEU:HD21	1:A:150:ILE:HD11	1.89	0.54
2:D:885:CYS:SG	2:D:887:HIS:NE2	2.82	0.53
1:A:72:LYS:CD	1:A:170:VAL:CG1	2.86	0.52
2:D:1014:SER:HA	2:D:1069:ARG:HH21	1.74	0.52
1:A:27:GLU:HG2	1:A:92:TYR:CD2	2.45	0.52
1:A:257:PHE:HB2	1:A:278:HIS:CD2	2.44	0.51
1:A:146:ILE:HG12	1:A:203:CYS:HA	1.91	0.51
1:A:89:TYR:HB2	1:A:101:VAL:HG23	1.91	0.51
1:A:113:ILE:HG21	1:A:293:LEU:HD13	1.91	0.51
2:C:982:ARG:NH1	2:C:983:ASP:O	2.44	0.51
1:B:133:THR:HG21	1:B:155:ILE:HD13	1.92	0.51
1:B:109:SER:O	1:B:113:ILE:HG12	2.11	0.51
1:A:132:SER:HB2	1:A:163:ALA:HB3	1.94	0.50
2:D:1046:PRO:HG2	2:D:1047:PRO:HD3	1.93	0.50
1:A:66:ASP:OD1	1:A:66:ASP:N	2.44	0.50
1:A:160:GLU:O	1:A:287:VAL:HG11	2.12	0.50
1:B:243:THR:OG1	1:B:244:PHE:N	2.45	0.49
2:C:901:CYS:SG	2:C:903:ASN:ND2	2.86	0.49
1:B:94:LYS:HB3	1:B:99:TRP:NE1	2.28	0.49
2:C:1045:ASP:HB3	2:C:1047:PRO:HD2	1.95	0.49
1:B:106:GLY:HA3	1:B:159:THR:HG23	1.94	0.48
2:D:861:ALA:O	2:D:865:GLN:HG2	2.14	0.48
2:D:1067:ILE:O	2:D:1069:ARG:HG3	2.13	0.48
1:B:192:ALA:O	1:B:195:VAL:HG12	2.14	0.48
2:C:907:ALA:HB1	2:C:922:LEU:HD23	1.95	0.48
2:D:1044:GLU:HG2	2:D:1045:ASP:H	1.78	0.47
2:D:962:ARG:HG2	2:D:963:ALA:N	2.28	0.47
2:C:1067:ILE:O	2:C:1069:ARG:HG3	2.15	0.47
1:B:59:ARG:NH2	1:B:167:ASP:OD1	2.45	0.47
2:C:862:MET:O	2:C:866:GLU:HG3	2.15	0.47
1:A:60:GLN:HB3	1:A:99:TRP:CE3	2.50	0.47
2:C:889:HIS:CD2	2:C:896:GLN:HG3	2.49	0.46
1:A:36:LEU:HD21	1:A:46:LYS:HB2	1.97	0.46
1:A:117:ARG:NH2	1:A:119:LYS:HD2	2.30	0.46
2:D:882:ARG:HG3	2:D:899:SER:HB2	1.97	0.46
1:A:60:GLN:HB3	1:A:99:TRP:CZ3	2.50	0.46
1:A:148:ARG:HB2	1:A:169:GLY:O	2.15	0.46
1:B:48:ILE:HG12	1:B:55:ILE:HG12	1.98	0.46

Continued on next page...

For Manuscript Review

Continued from previous page...

Atom-1	Atom-2	Interatomic distance (Å)	Clash overlap (Å)
1:B:91:SER:HB3	1:B:98:LEU:HD11	1.98	0.46
1:A:58:ILE:HG12	1:A:101:VAL:HG12	1.97	0.45
1:A:72:LYS:HD3	1:A:170:VAL:HG12	1.97	0.45
2:D:989:GLU:O	2:D:991:PRO:HD3	2.16	0.45
1:A:86:VAL:HG21	1:A:166:ALA:HB2	1.97	0.45
1:B:74:ILE:HD11	1:B:100:ILE:HD13	1.97	0.45
1:B:285:LYS:HB2	1:B:289:ILE:HD13	1.98	0.45
2:D:1011:ASN:O	2:D:1069:ARG:NE	2.49	0.44
1:B:188:PRO:HA	1:B:191:MET:HG3	1.99	0.44
1:B:192:ALA:O	1:B:196:ILE:HG22	2.18	0.44
2:C:969:CYS:HB3	2:C:986:CYS:SG	2.58	0.44
2:C:1064:GLY:O	2:C:1069:ARG:NH1	2.50	0.44
1:B:60:GLN:O	1:B:62:PRO:HD3	2.18	0.44
1:A:72:LYS:HG2	1:A:170:VAL:CG1	2.39	0.44
2:D:941:ARG:HH22	2:D:1025:GLU:CD	2.20	0.44
2:D:901:CYS:HB3	2:D:930:CYS:SG	2.58	0.44
2:D:982:ARG:HH11	2:D:983:ASP:H	1.66	0.44
1:B:115:ARG:HE	1:B:115:ARG:HB2	1.61	0.43
1:A:60:GLN:O	1:A:62:PRO:HD3	2.18	0.43
1:A:94:LYS:HB3	1:A:99:TRP:NE1	2.34	0.43
1:A:25:GLN:HA	1:A:26:PRO:HD3	1.79	0.43
1:A:103:GLU:OE2	1:A:164:LYS:NZ	2.41	0.43
2:C:868:GLY:O	2:C:882:ARG:NH1	2.52	0.43
1:B:257:PHE:HB2	1:B:278:HIS:CE1	2.54	0.43
1:A:16:LYS:HB3	1:A:16:LYS:HE2	1.81	0.43
1:A:229:PRO:O	1:A:233:ILE:HG13	2.18	0.43
1:B:36:LEU:HD11	1:B:104:TYR:HE1	1.83	0.42
1:B:235:MET:HG2	2:C:877:SER:O	2.18	0.42
2:C:901:CYS:HA	2:C:1067:ILE:HG12	2.01	0.42
1:B:35:LYS:HD3	1:B:38:GLU:HB2	2.01	0.42
1:B:106:GLY:HA3	1:B:159:THR:CG2	2.50	0.42
2:C:979:ASN:HD21	1:A:220:GLY:HA3	1.84	0.42
2:C:1046:PRO:HG2	2:C:1047:PRO:HD3	2.01	0.42
2:D:971:VAL:HG11	2:D:1001:HIS:CD2	2.54	0.42
2:C:1010:ILE:HG23	2:C:1015:LEU:HB2	1.99	0.42
2:D:1023:VAL:HG22	2:D:1050:GLN:HB2	2.01	0.42
2:D:1045:ASP:HB3	2:D:1048:ALA:HB3	2.00	0.42
1:B:119:LYS:HE3	1:B:119:LYS:HB2	1.79	0.42
1:A:113:ILE:HG23	1:A:297:ALA:HB2	2.01	0.42
2:C:1044:GLU:HG2	2:C:1045:ASP:H	1.85	0.42
1:B:130:LEU:HD23	1:B:130:LEU:HA	1.89	0.41

Continued on next page...

For Manuscript Review

Continued from previous page...

Atom-1	Atom-2	Interatomic distance (Å)	Clash overlap (Å)
1:A:192:ALA:HA	1:A:208:TRP:CD1	2.55	0.41
2:C:1003:LYS:O	2:C:1007:VAL:HG23	2.20	0.41
2:D:1046:PRO:CG	2:D:1047:PRO:HD3	2.50	0.41
2:D:896:GLN:HB3	2:D:905:PHE:HB3	2.02	0.41
2:C:861:ALA:O	2:C:865:GLN:HG3	2.21	0.41
1:A:72:LYS:CD	1:A:170:VAL:HG12	2.51	0.40
2:C:920:LYS:HE3	2:C:920:LYS:HB2	1.91	0.40
2:C:1018:ALA:HA	2:C:1021:TYR:CG	2.56	0.40
2:D:901:CYS:HA	2:D:1067:ILE:HG12	2.02	0.40

There are no symmetry-related clashes.

5.3 Torsion angles (i)

5.3.1 Protein backbone (i)

In the following table, the Percentiles column shows the percent Ramachandran outliers of the chain as a percentile score with respect to all X-ray entries followed by that with respect to entries of similar resolution.

The Analysed column shows the number of residues for which the backbone conformation was analysed, and the total number of residues.

Mol	Chain	Analysed	Favoured	Allowed	Outliers	Percentiles	
1	A	283/302 (94%)	270 (95%)	13 (5%)	0	100	100
1	B	286/302 (95%)	276 (96%)	10 (4%)	0	100	100
2	C	213/215 (99%)	205 (96%)	8 (4%)	0	100	100
2	D	213/215 (99%)	204 (96%)	9 (4%)	0	100	100
All	All	995/1034 (96%)	955 (96%)	40 (4%)	0	100	100

There are no Ramachandran outliers to report.

5.3.2 Protein sidechains (i)

In the following table, the Percentiles column shows the percent sidechain outliers of the chain as a percentile score with respect to all X-ray entries followed by that with respect to entries of similar resolution.

The Analysed column shows the number of residues for which the sidechain conformation was analysed, and the total number of residues.

For Manuscript Review

Mol	Chain	Analysed	Rotameric	Outliers	Percentiles	
1	A	251/266 (94%)	250 (100%)	1 (0%)	91	96
1	B	254/266 (96%)	254 (100%)	0	100	100
2	C	178/179 (99%)	177 (99%)	1 (1%)	86	95
2	D	178/179 (99%)	177 (99%)	1 (1%)	86	95
All	All	861/890 (97%)	858 (100%)	3 (0%)	92	97

All (3) residues with a non-rotameric sidechain are listed below:

Mol	Chain	Res	Type
2	C	919	LYS
1	A	29	VAL
2	D	965	PRO

Sometimes sidechains can be flipped to improve hydrogen bonding and reduce clashes. All (5) such sidechains are listed below:

Mol	Chain	Res	Type
1	B	141	HIS
2	C	915	ASN
1	A	278	HIS
2	D	915	ASN
2	D	974	GLN

5.3.3 RNA (i)

There are no RNA molecules in this entry.

5.4 Non-standard residues in protein, DNA, RNA chains (i)

There are no non-standard protein/DNA/RNA residues in this entry.

5.5 Carbohydrates (i)

There are no monosaccharides in this entry.

5.6 Ligand geometry (i)

Of 10 ligands modelled in this entry, 8 are monoatomic - leaving 2 for Mogul analysis.

For Manuscript Review

In the following table, the Counts columns list the number of bonds (or angles) for which Mogul statistics could be retrieved, the number of bonds (or angles) that are observed in the model and the number of bonds (or angles) that are defined in the Chemical Component Dictionary. The Link column lists molecule types, if any, to which the group is linked. The Z score for a bond length (or angle) is the number of standard deviations the observed value is removed from the expected value. A bond length (or angle) with $|Z| > 2$ is considered an outlier worth inspection. RMSZ is the root-mean-square of all Z scores of the bond lengths (or angles).

Mol	Type	Chain	Res	Link	Bond lengths			Bond angles		
					Counts	RMSZ	$\#\|Z\| > 2$	Counts	RMSZ	$\#\|Z\| > 2$
3	GOL	B	401	-	5,5,5	0.92	0	5,5,5	0.99	0
3	GOL	A	401	-	5,5,5	0.89	0	5,5,5	1.02	0

In the following table, the Chirals column lists the number of chiral outliers, the number of chiral centers analysed, the number of these observed in the model and the number defined in the Chemical Component Dictionary. Similar counts are reported in the Torsion and Rings columns. '-' means no outliers of that kind were identified.

Mol	Type	Chain	Res	Link	Chirals	Torsions	Rings
3	GOL	B	401	-	-	0/4/4/4	-
3	GOL	A	401	-	-	2/4/4/4	-

There are no bond length outliers.

There are no bond angle outliers.

There are no chirality outliers.

All (2) torsion outliers are listed below:

Mol	Chain	Res	Type	Atoms
3	A	401	GOL	C1-C2-C3-O3
3	A	401	GOL	O2-C2-C3-O3

There are no ring outliers.

No monomer is involved in short contacts.

The following is a two-dimensional graphical depiction of Mogul quality analysis of bond lengths, bond angles, torsion angles, and ring geometry for all instances of the Ligand of Interest. In addition, ligands with molecular weight > 250 and outliers as shown on the validation Tables will also be included. For torsion angles, if less then 5% of the Mogul distribution of torsion angles is within 10 degrees of the torsion angle in question, then that torsion angle is considered an outlier. Any bond that is central to one or more torsion angles identified as an outlier by Mogul will be highlighted in the graph. For rings, the root-mean-square deviation (RMSD) between the ring in question and similar rings identified by Mogul is calculated over all ring torsion angles. If the average RMSD is greater than 60 degrees and the minimal RMSD between the ring in question and

For Manuscript Review

any Mogul-identified rings is also greater than 60 degrees, then that ring is considered an outlier. The outliers are highlighted in purple. The color gray indicates Mogul did not find sufficient equivalents in the CSD to analyse the geometry.

Ligand GOL B 401

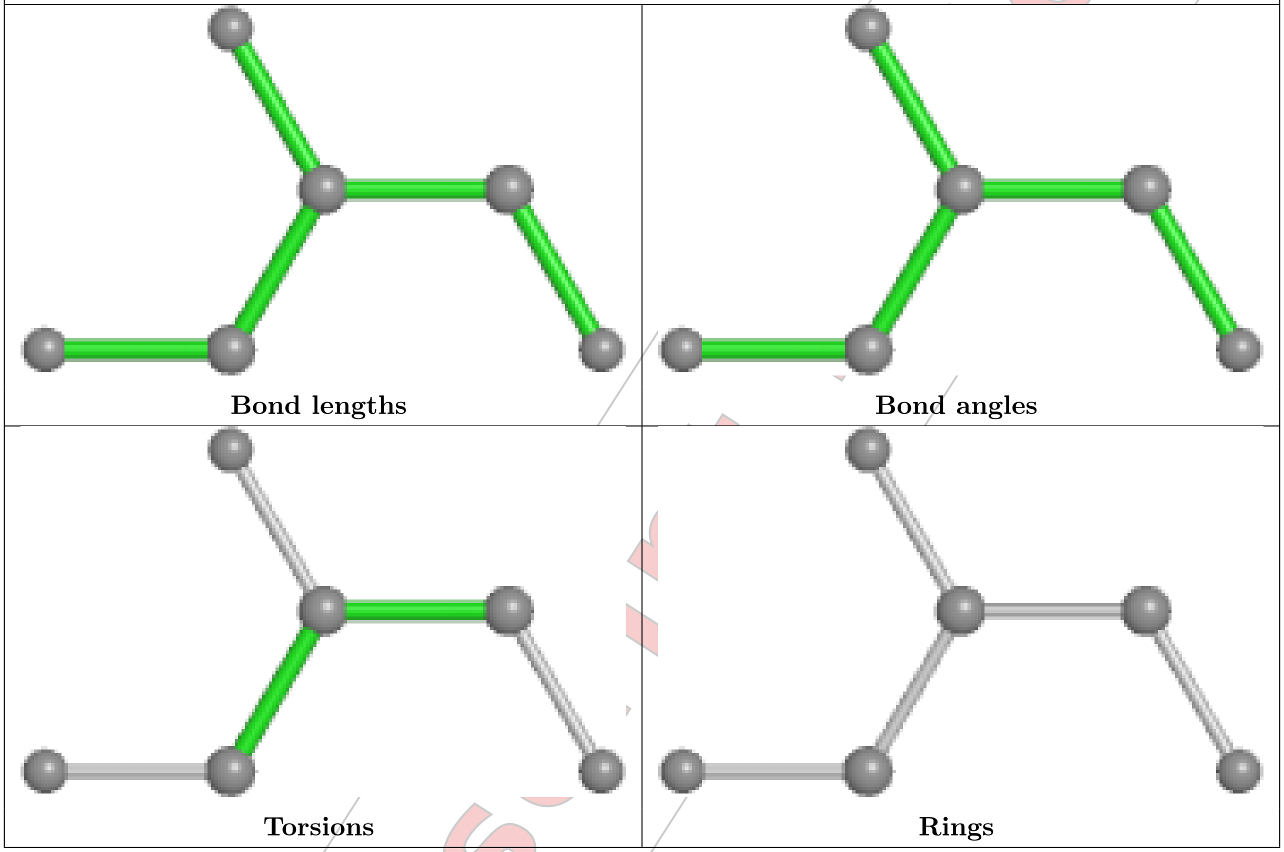

For Manuscript Review

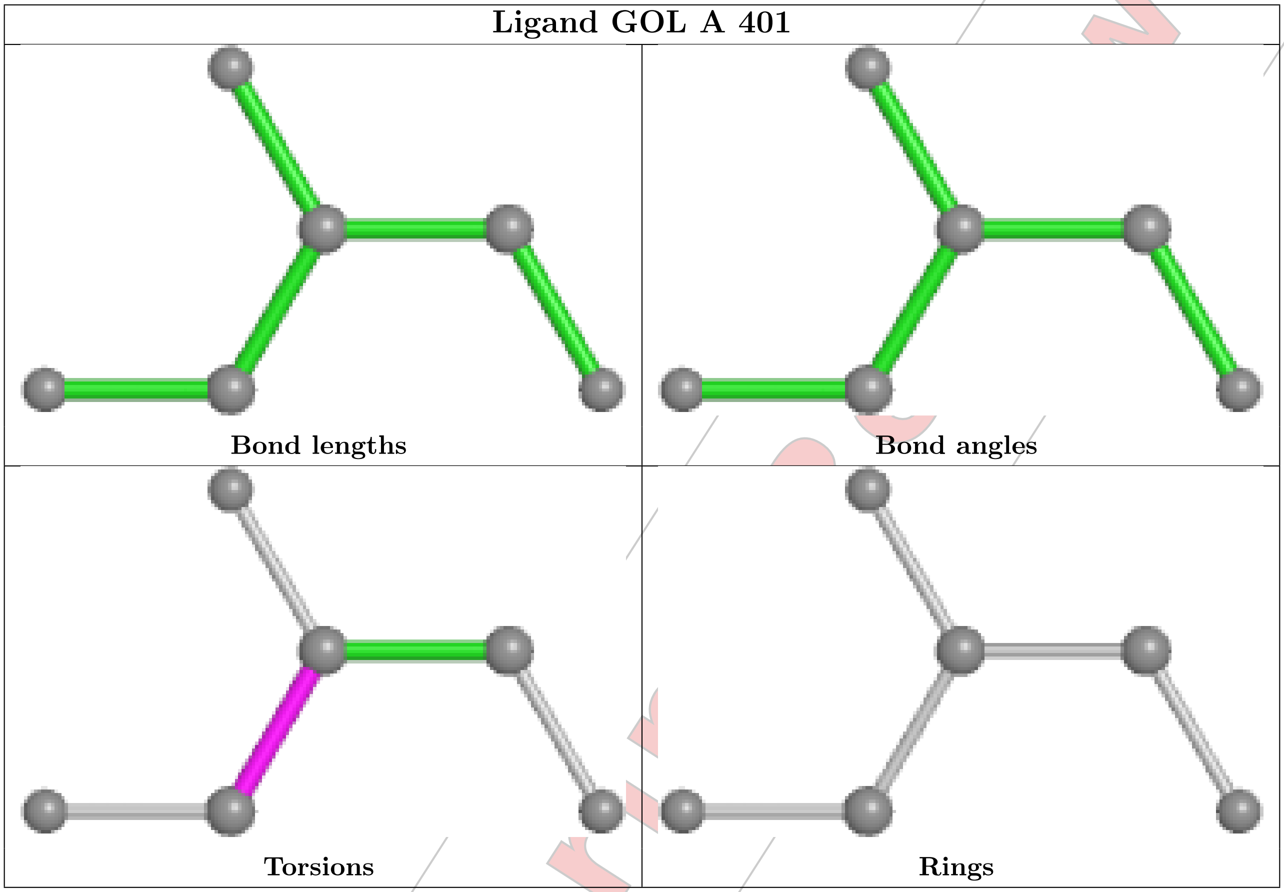

5.7 Other polymers (i)

There are no such residues in this entry.

5.8 Polymer linkage issues (i)

There are no chain breaks in this entry.

For Manuscript Review

6 Fit of model and data (i)

6.1 Protein, DNA and RNA chains (i)

In the following table, the column labelled '#RSRZ> 2' contains the number (and percentage) of RSRZ outliers, followed by percent RSRZ outliers for the chain as percentile scores relative to all X-ray entries and entries of similar resolution. The OWAB column contains the minimum, median, 95th percentile and maximum values of the occupancy-weighted average B-factor per residue. The column labelled 'Q< 0.9' lists the number of (and percentage) of residues with an average occupancy less than 0.9.

Mol	Chain	Analysed	<RSRZ>	#RSRZ>2			OWAB(Å^2)	Q<0.9
1	A	287/302 (95%)	0.41	6 (2%)	63	59	60, 98, 147, 182	0
1	B	290/302 (96%)	0.39	6 (2%)	63	59	55, 86, 149, 193	0
2	C	215/215 (100%)	0.86	30 (13%)	2	2	70, 120, 200, 219	0
2	D	215/215 (100%)	1.04	37 (17%)	1	1	86, 136, 206, 249	0
All	All	1007/1034 (97%)	0.64	79 (7%)	13	9	55, 103, 188, 249	0

All (79) RSRZ outliers are listed below:

Mol	Chain	Res	Type	RSRZ
2	D	1035	HIS	9.8
2	C	908	LYS	7.4
2	D	873	LYS	6.1
2	D	914	PRO	5.6
2	D	922	LEU	5.5
1	A	36	LEU	4.9
2	D	905	PHE	4.8
2	C	921	SER	4.8
2	D	896	GLN	4.6
2	C	979	ASN	4.0
2	C	906	TYR	4.0
2	D	1021	TYR	4.0
2	D	1052	ARG	3.9
2	C	1035	HIS	3.7
2	D	897	PHE	3.6
2	D	1041	LEU	3.6
2	C	915	ASN	3.6
2	D	1054	LEU	3.6
2	C	1041	LEU	3.5
2	D	1038	PRO	3.5
2	D	1063	LEU	3.5

Continued on next page...

For Manuscript Review

Continued from previous page...

Mol	Chain	Res	Type	RSRZ
2	D	888	PHE	3.4
2	C	1062	PRO	3.3
2	C	922	LEU	3.3
2	D	1060	GLU	3.2
2	C	917	ARG	3.2
2	C	1023	VAL	3.2
2	D	1057	LEU	3.1
1	A	281	VAL	3.0
2	D	1071	ARG	3.0
2	C	907	ALA	3.0
2	D	1036	VAL	2.9
2	D	1039	GLN	2.9
2	C	918	VAL	2.9
2	C	873	LYS	2.8
2	D	903	ASN	2.8
2	C	962	ARG	2.7
2	C	1038	PRO	2.7
2	D	915	ASN	2.7
2	D	942	LEU	2.7
2	C	1044	GLU	2.7
2	C	1030	THR	2.7
2	D	1023	VAL	2.6
2	D	1061	VAL	2.6
1	B	281	VAL	2.6
1	A	173	GLN	2.6
1	A	230	MET	2.6
2	D	862	MET	2.6
2	C	879	ALA	2.5
2	D	878	TYR	2.5
1	A	12	ARG	2.5
1	B	33	LEU	2.4
2	C	897	PHE	2.4
2	C	1033	TYR	2.4
2	D	917	ARG	2.4
2	C	1036	VAL	2.4
2	D	1033	TYR	2.4
2	D	912	PRO	2.3
2	C	862	MET	2.3
2	D	967	GLY	2.3
2	D	940	LEU	2.2
2	D	1047	PRO	2.2
2	D	1068	PRO	2.2

Continued on next page...

For Manuscript Review

Continued from previous page...

Mol	Chain	Res	Type	RSRZ
2	D	879	ALA	2.2
2	C	905	PHE	2.2
2	D	868	GLY	2.2
2	C	1058	THR	2.2
1	B	12	ARG	2.2
2	C	894	ARG	2.2
1	B	67	LEU	2.1
2	C	909	ASN	2.1
1	A	27	GLU	2.1
2	C	868	GLY	2.1
1	B	63	VAL	2.0
2	D	1042	ALA	2.0
2	C	920	LYS	2.0
2	C	1034	LEU	2.0
1	B	170	VAL	2.0
2	D	895	HIS	2.0

6.2 Non-standard residues in protein, DNA, RNA chains (i)

There are no non-standard protein/DNA/RNA residues in this entry.

6.3 Carbohydrates (i)

There are no monosaccharides in this entry.

6.4 Ligands (i)

In the following table, the Atoms column lists the number of modelled atoms in the group and the number defined in the chemical component dictionary. The B-factors column lists the minimum, median, 95th percentile and maximum values of B factors of atoms in the group. The column labelled 'Q< 0.9' lists the number of atoms with occupancy less than 0.9.

Mol	Type	Chain	Res	Atoms	RSCC	RSR	B-factors($Å^2$)	Q<0.9
4	ZN	D	1104	1/1	0.63	0.09	190,190,190,190	0
4	ZN	D	1102	1/1	0.74	0.20	131,131,131,131	0
4	ZN	C	1104	1/1	0.74	0.08	158,158,158,158	0
4	ZN	D	1103	1/1	0.82	0.18	99,99,99,99	0
3	GOL	A	401	6/6	0.83	0.24	97,103,111,112	0
3	GOL	B	401	6/6	0.84	0.22	76,78,83,85	0
4	ZN	C	1101	1/1	0.92	0.21	118,118,118,118	0

Continued on next page...

For Manuscript Review

Continued from previous page...

Mol	Type	Chain	Res	Atoms	RSCC	RSR	B-factors($Å^2$)	Q<0.9
4	ZN	D	1101	1/1	0.93	0.15	130,130,130,130	0
4	ZN	C	1103	1/1	0.98	0.21	94,94,94,94	0
4	ZN	C	1102	1/1	0.99	0.16	85,85,85,85	0

The following is a graphical depiction of the model fit to experimental electron density of all instances of the Ligand of Interest. In addition, ligands with molecular weight > 250 and outliers as shown on the geometry validation Tables will also be included. Each fit is shown from different orientation to approximate a three-dimensional view.

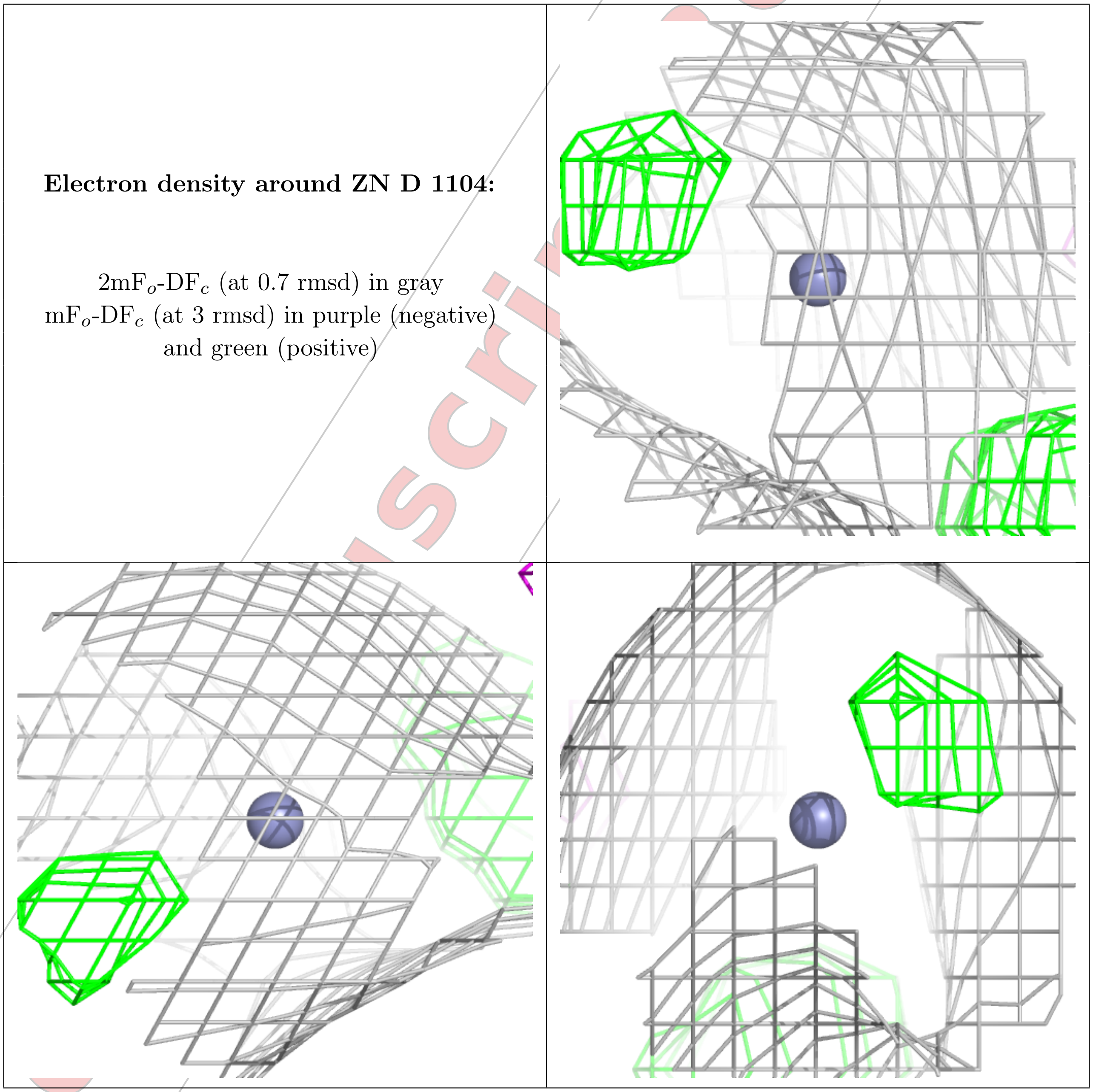

Electron density around ZN D 1104:

$2mF_o$-DF_c (at 0.7 rmsd) in gray
mF_o-DF_c (at 3 rmsd) in purple (negative)
and green (positive)

For Manuscript Review

Electron density around ZN D 1102:

2mF$_o$-DF$_c$ (at 0.7 rmsd) in gray
mF$_o$-DF$_c$ (at 3 rmsd) in purple (negative)
and green (positive)

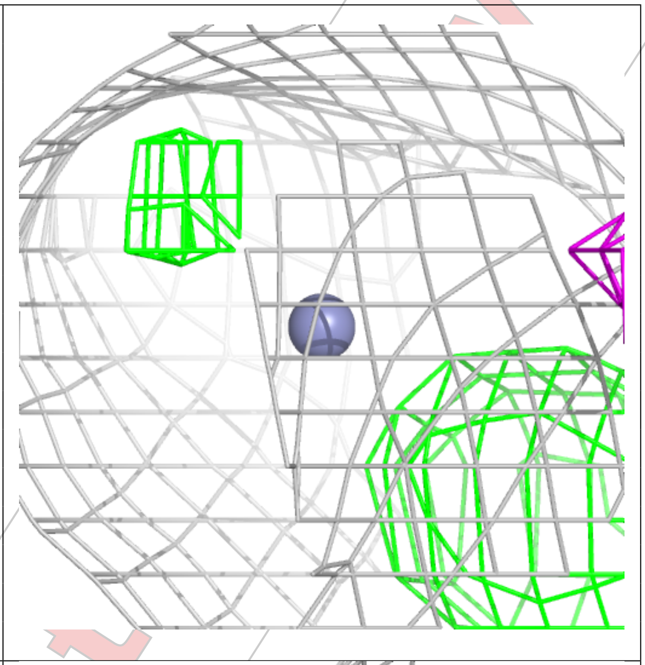

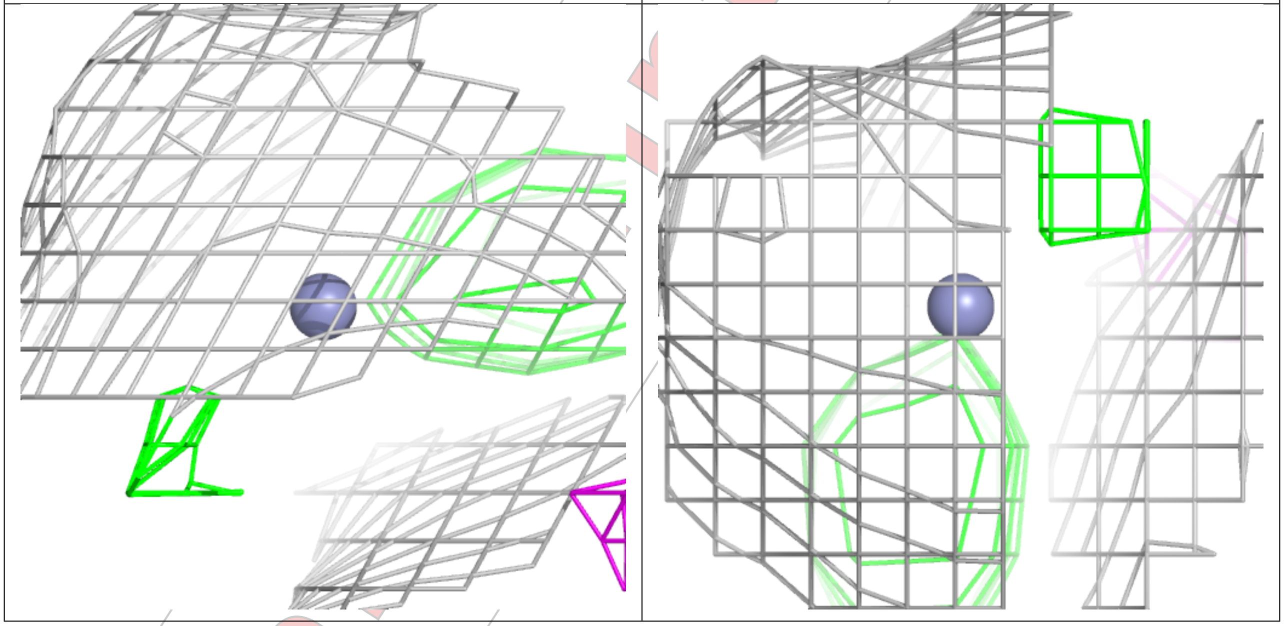

For Manuscript Review

Electron density around ZN C 1104:

2mF$_o$-DF$_c$ (at 0.7 rmsd) in gray
mF$_o$-DF$_c$ (at 3 rmsd) in purple (negative)
and green (positive)

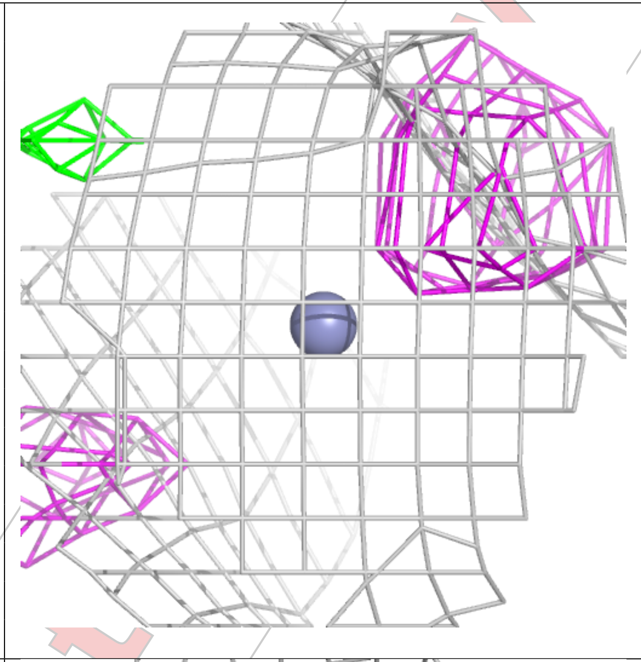

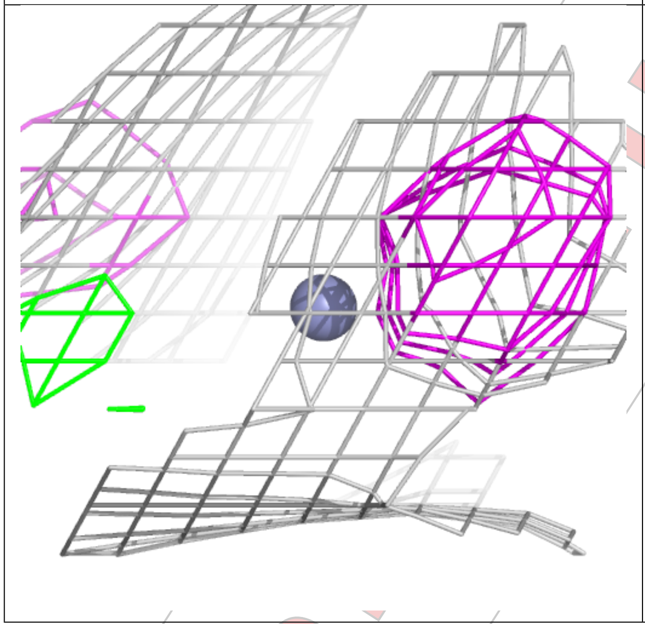

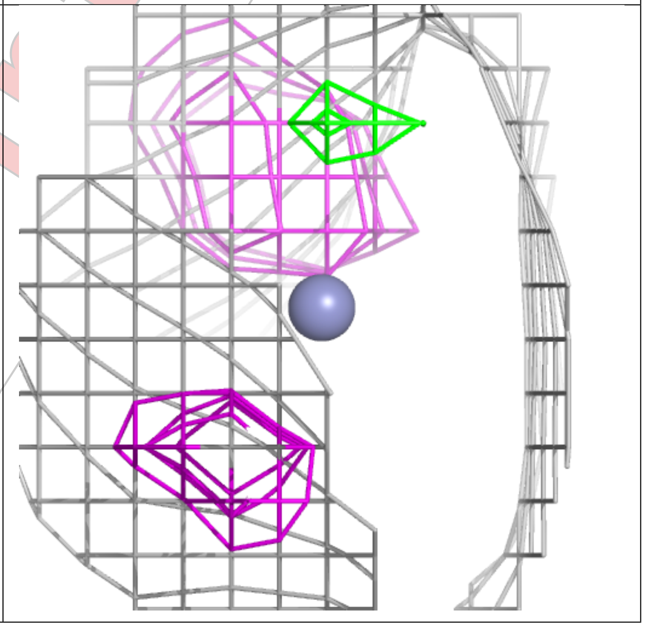

For Manuscript Review

Electron density around ZN D 1103:

2mF$_o$-DF$_c$ (at 0.7 rmsd) in gray
mF$_o$-DF$_c$ (at 3 rmsd) in purple (negative)
and green (positive)

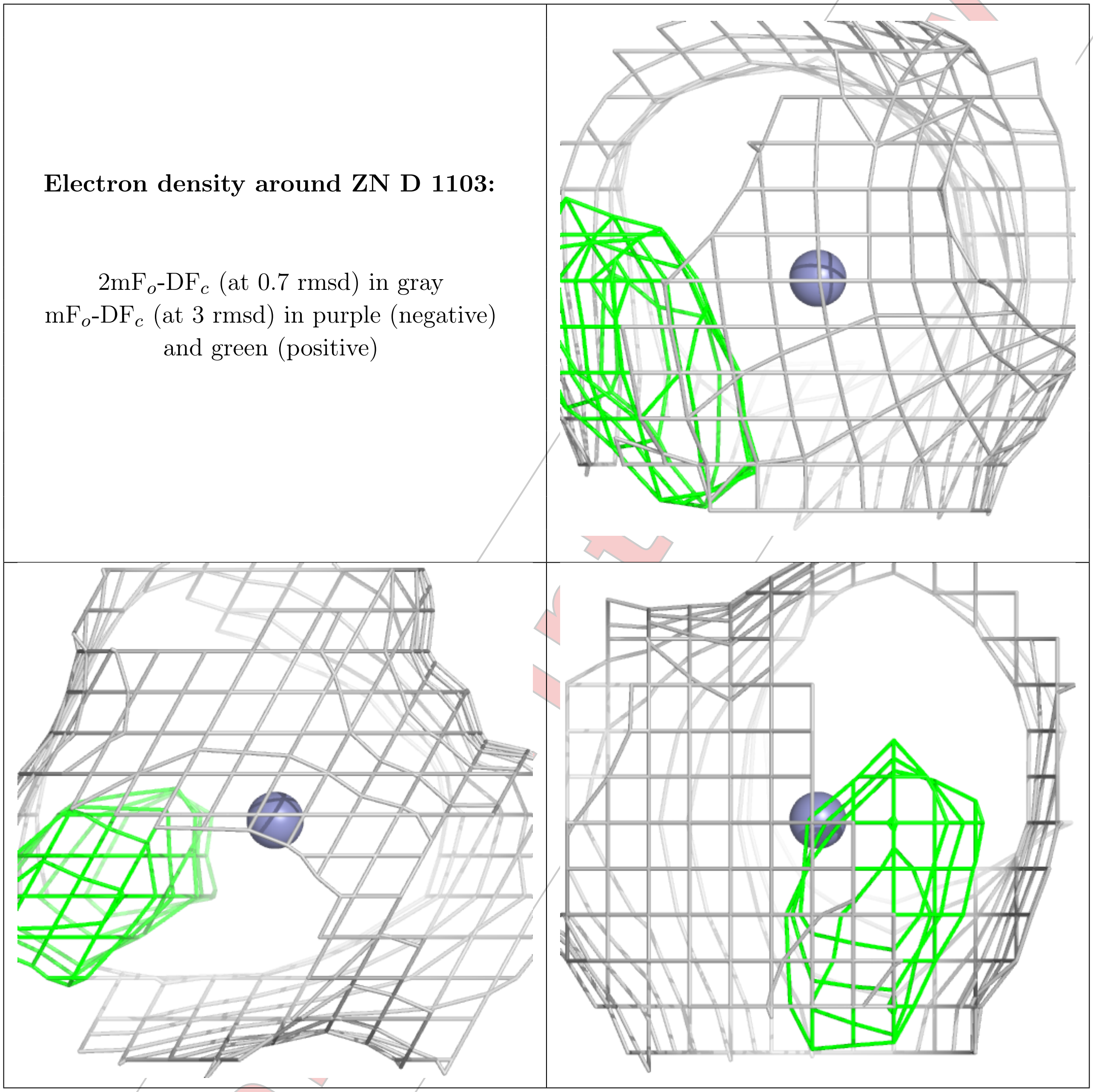

For Manuscript Review

Electron density around GOL A 401:

$2mF_o$-DF_c (at 0.7 rmsd) in gray
mF_o-DF_c (at 3 rmsd) in purple (negative)
and green (positive)

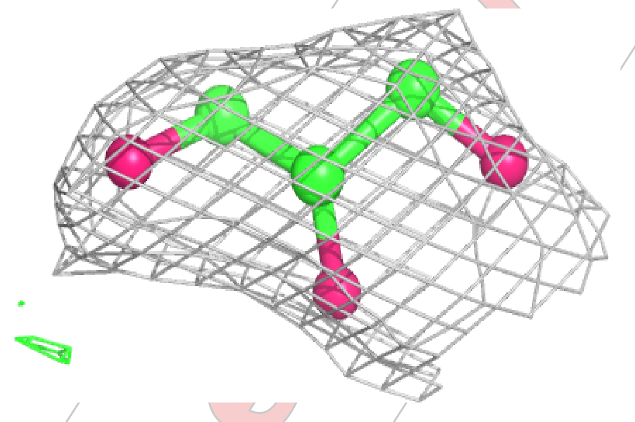

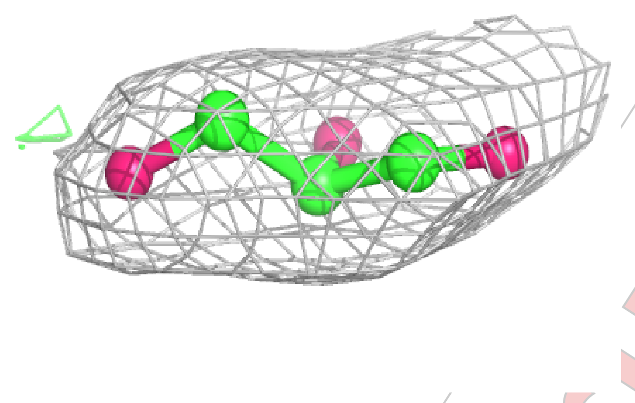

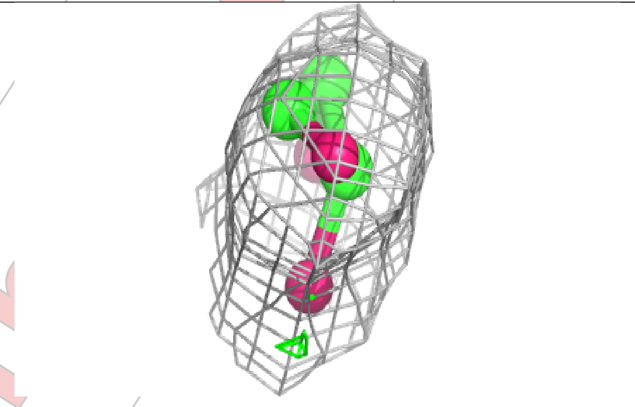

Electron density around GOL B 401:

$2mF_o$-DF_c (at 0.7 rmsd) in gray
mF_o-DF_c (at 3 rmsd) in purple (negative)
and green (positive)

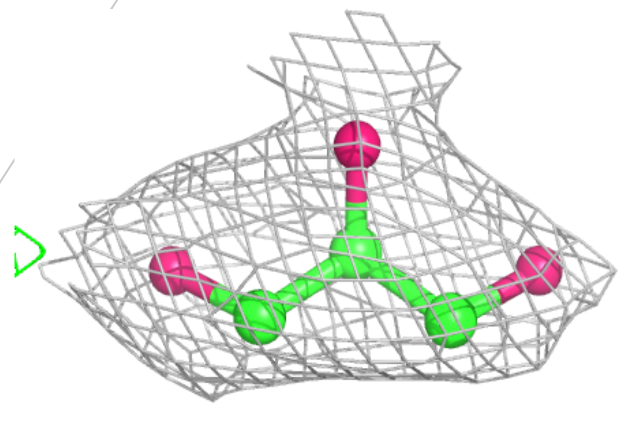

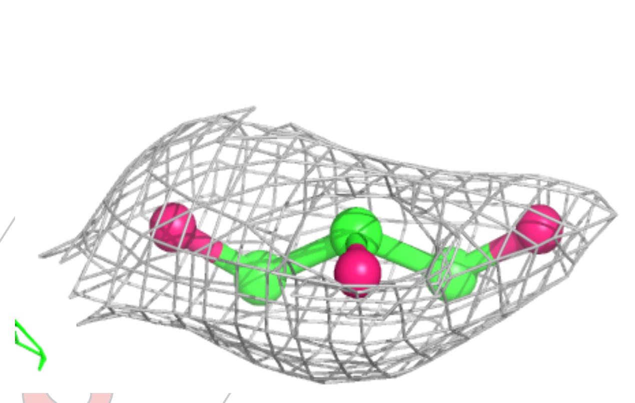

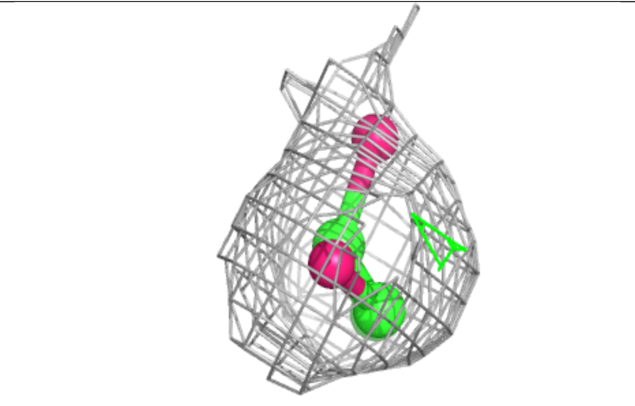

Electron density around ZN C 1101:

$2mF_o$-DF_c (at 0.7 rmsd) in gray
mF_o-DF_c (at 3 rmsd) in purple (negative)
and green (positive)

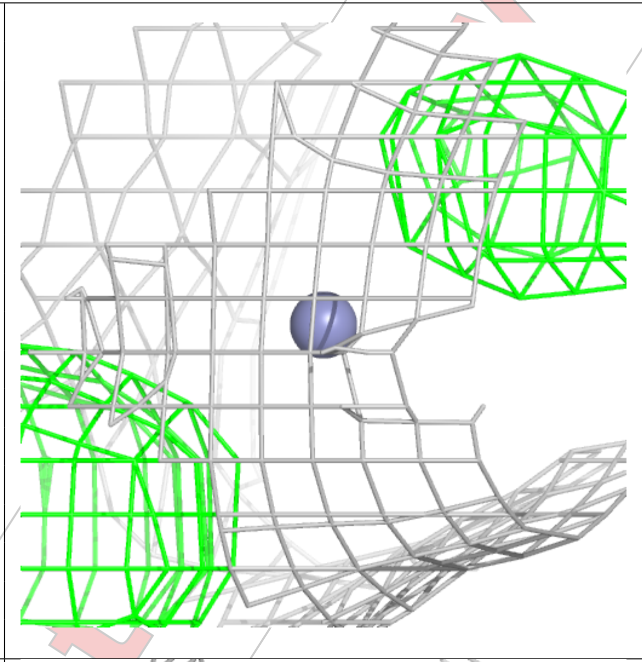

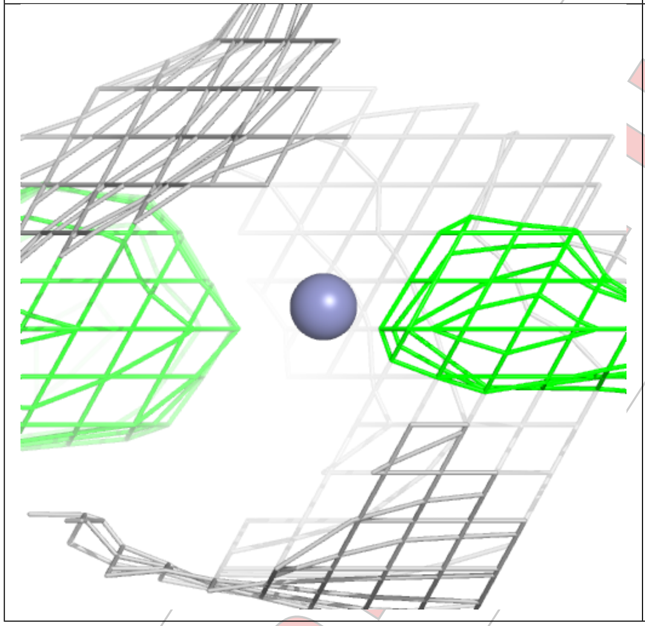

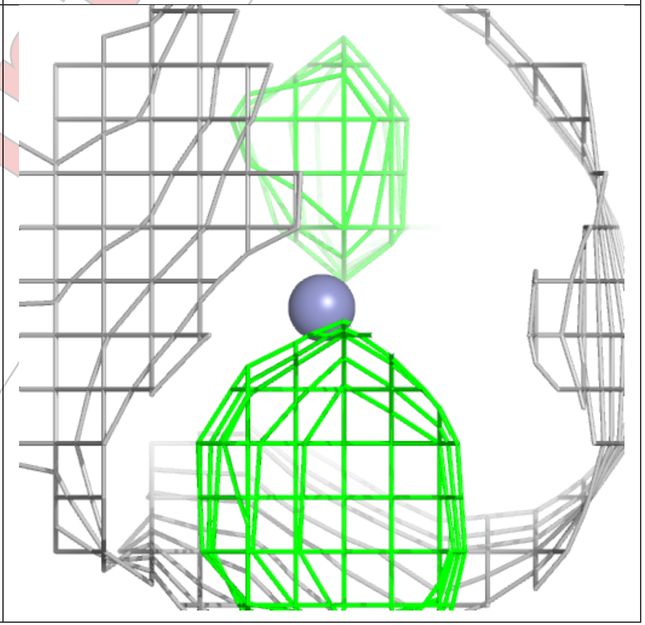

For Manuscript Review

Electron density around ZN D 1101:

2mF$_o$-DF$_c$ (at 0.7 rmsd) in gray
mF$_o$-DF$_c$ (at 3 rmsd) in purple (negative)
and green (positive)

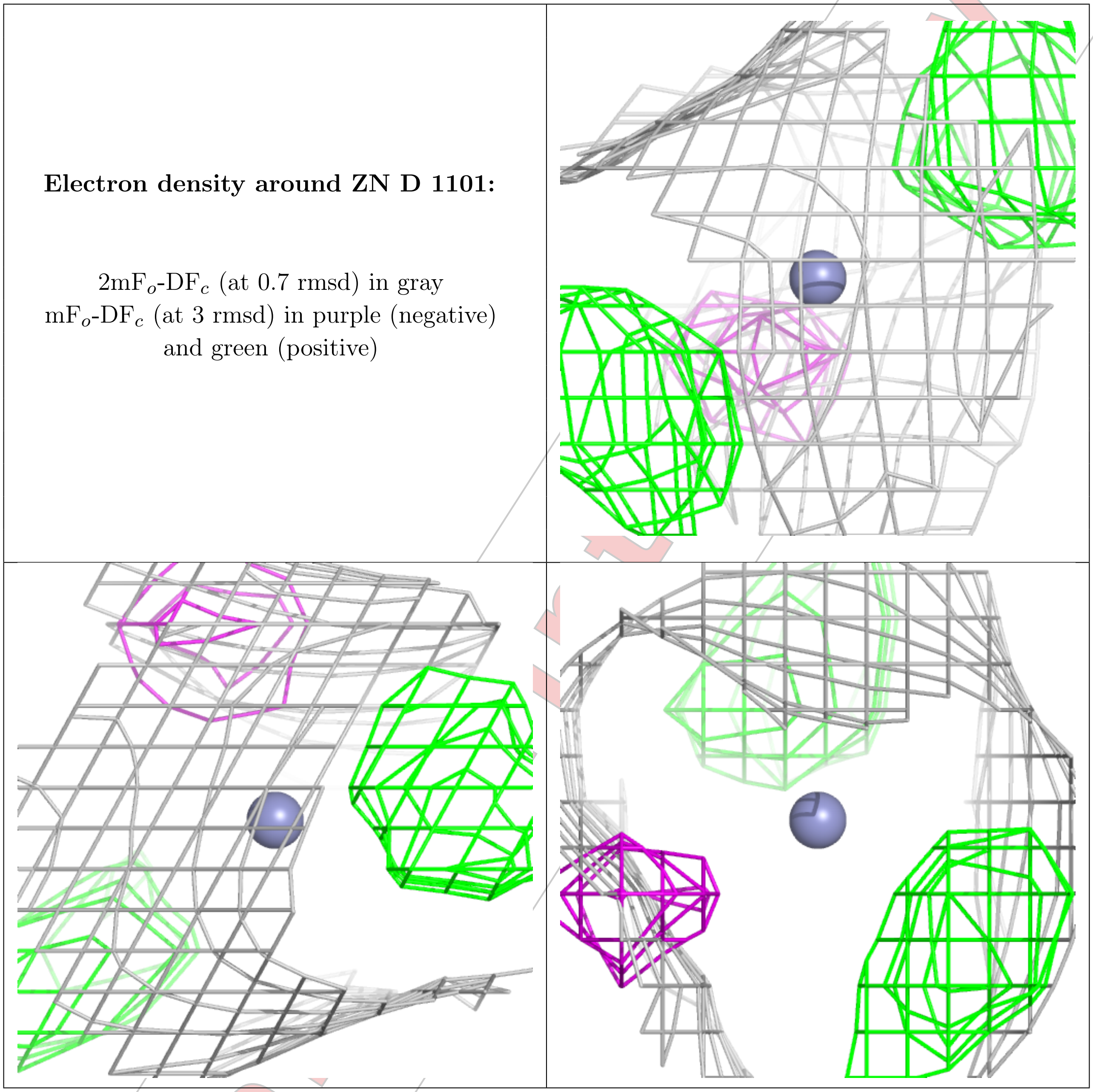

For Manuscript Review

Electron density around ZN C 1103:

$2mF_o$-DF_c (at 0.7 rmsd) in gray
mF_o-DF_c (at 3 rmsd) in purple (negative)
and green (positive)

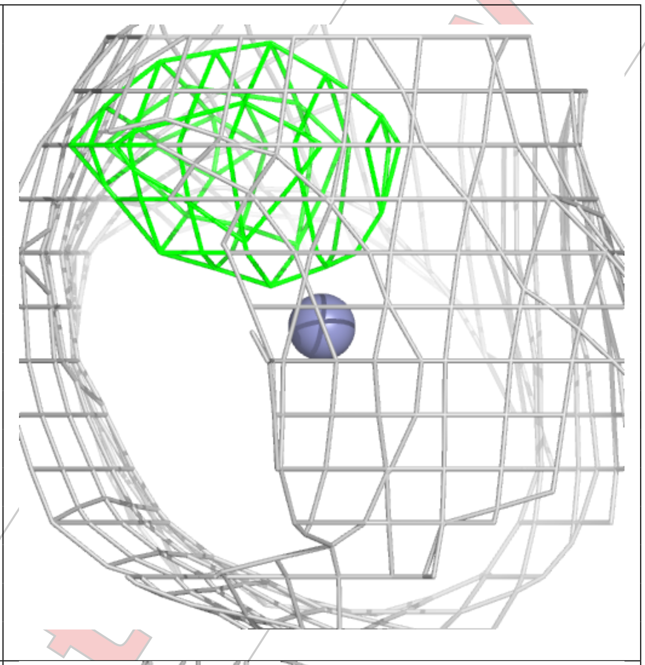

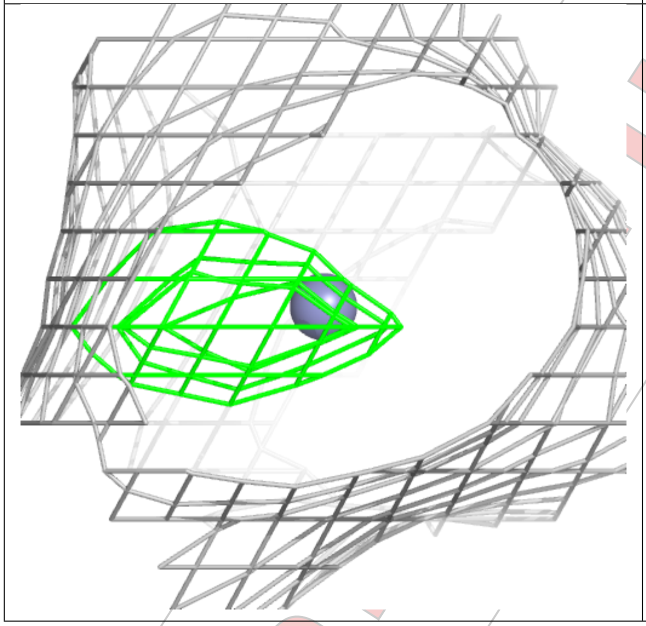

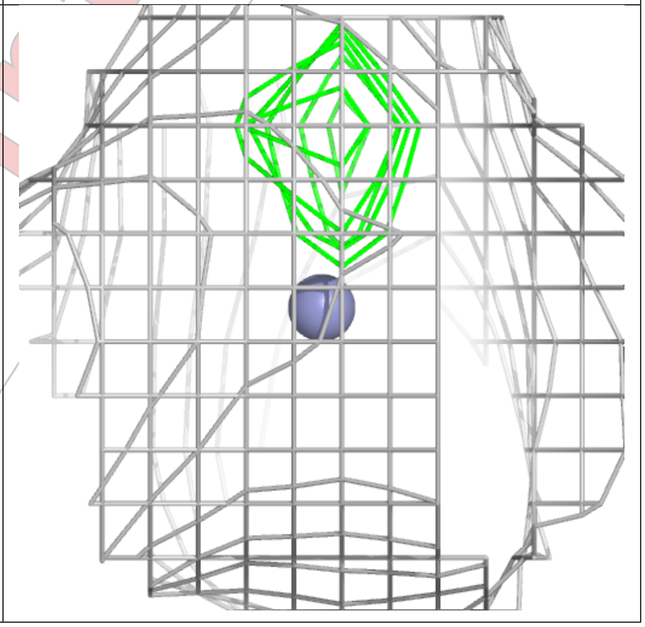

Electron density around ZN C 1102:

$2mF_o$-DF_c (at 0.7 rmsd) in gray
mF_o-DF_c (at 3 rmsd) in purple (negative)
and green (positive)

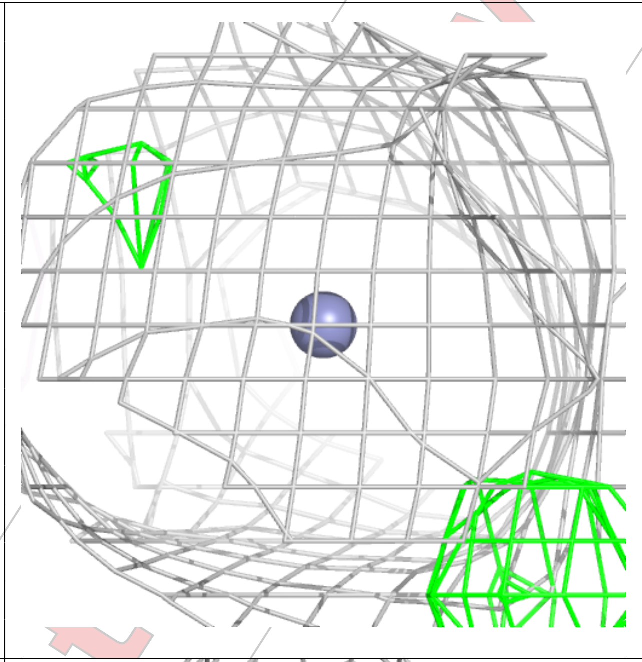

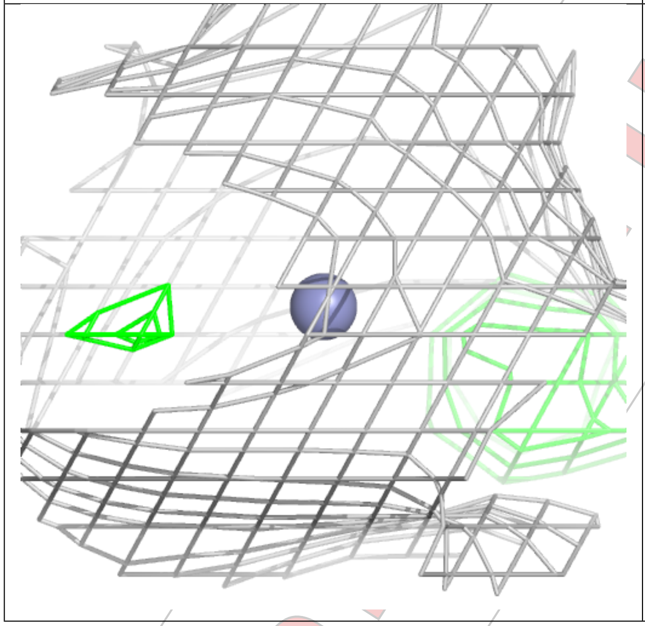

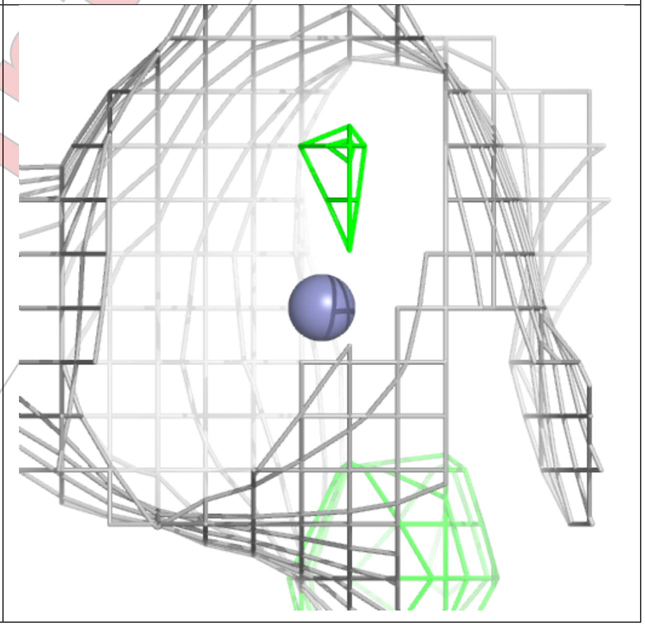

6.5 Other polymers ⓘ

There are no such residues in this entry.

For Manuscript Review
