# Supplementary material for: STK4 inhibits the E3 activity of HOIP by phosphorylating its allosteric ubiquitin-binding site
Source: Cell Discov. 2025 Sep 16;11:75. doi: 10.1038/s41421-025-00824-x (PMC12441119; doi:10.1038/s41421-025-00824-x)
Supplement: Supplementary file 2 — The Supplemental Figures and Table [file 41421_2025_824_MOESM2_ESM.pdf]

Supplementary Materials for

STK4 inhibits the E3 activity of HOIP by phosphorylating its allosteric ubiquitin-binding site

Yaru Wang, Xindi Zhou, Zhiqiao Lin, Yichao Huang, Yuchao Zhang, Haobo Liu, Yuqian Zhou, Jianping Liu*, and Lifeng Pan*

Supplementary Figures

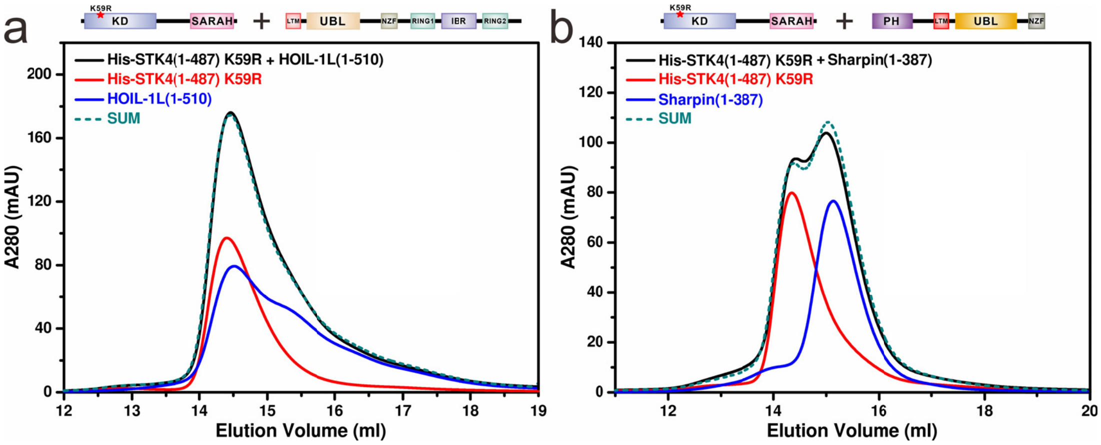

Supplementary Fig. S1. HOIL-1L and Sharpin are unable to interact with STK4. a SEC-based analysis of the interaction of 20 μM full-length STK4 K59R mutant with 15 μM full-length HOIL-1L. **b** SEC-based analysis of the interaction of 20 μM full-length STK4 K59R mutant with 30 μM full-length Sharpin. The SEC-based assays were performed using a Superdex 200 Increase 10/300 GL column (GE Healthcare). The "SUM" stands for the theoretical sum of the SEC profiles of the two indicated proteins. These experiments have been replicated three times.

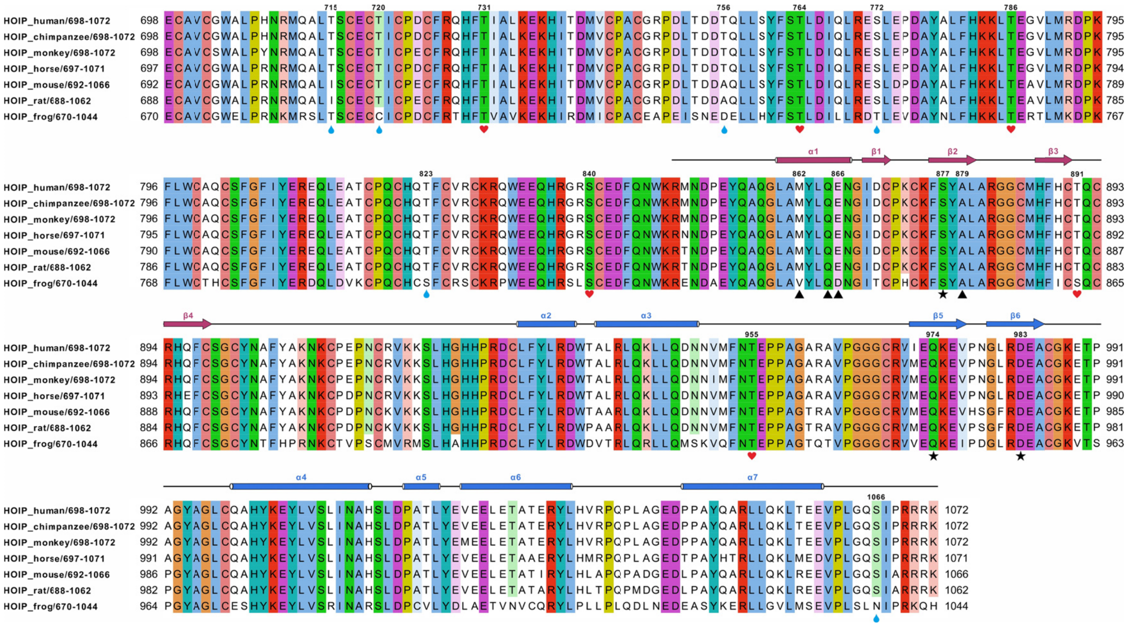

Supplementary Fig. S2. Structure-based sequence alignment analysis of HOIP from different species. In this alignment, the conserved residues are highlighted by colors using software Jalview 2.11.2.6 (http://www.jalview.org/). The binding interface residues in the HOIP RING2-LDD domain, which are important for the interaction of STK4 with HOIP, are highlighted with black stars (polar interactions) and black triangles (hydrophobic interactions). The phosphorylation sites detected by mass spectrometry are highlighted by heart-shape and water-shape, which represent the conserved and non-conserved serine or threonine, respectively.

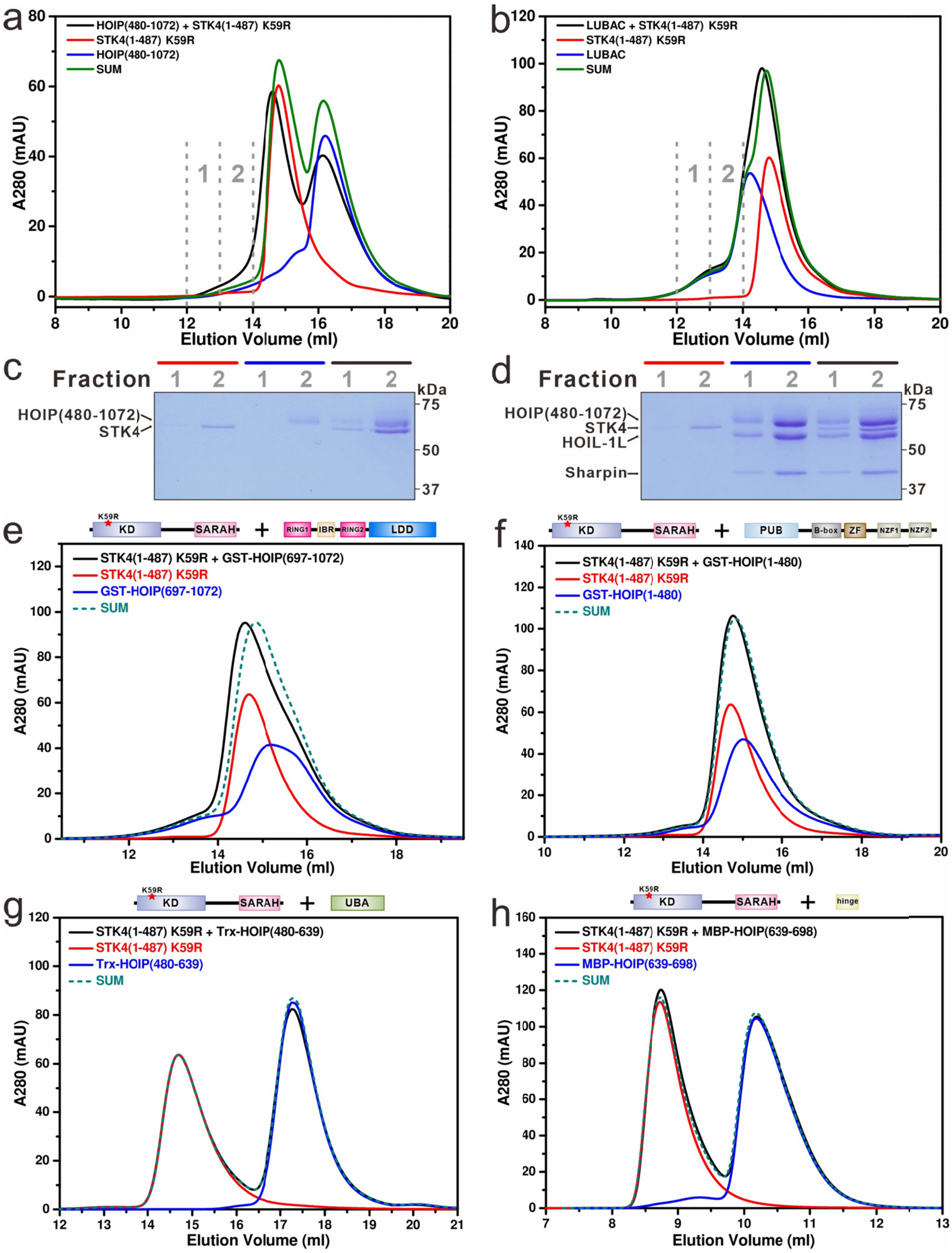

Supplementary Fig. S3. Biochemical mapping of the STK4-binding region of HOIP using SEC-based analyses. a, **b** SEC-based analyses of 20 μM STK4(1-487) K59R with 12 μM HOIP(480-1072) (**a**) and 5 μM HOIP(480-1072)-containing LUBAC complex (**b**). The LUABC complex used in this assay consists of HOIP(480-1072), the full-length

HOIL-1L C460A mutant and the full-length Sharpin purified from *E. coil* cells. This assay was performed using a Superdex 200 Increase 10/300 GL column (GE Healthcare). **c**, **d** SDS-PAGE combined with Coomassie blue staining analyses showing the protein components of the corresponding fraction 1 and 2 collected from the SEC-based experiments in panel **a** (**c**), or panel **b** (**d**). **e**-**h** SEC-based analyses of the interaction of 20 μM full-length STK4 K59R with 11 μM HOIP(697-1072) (**e**), 11 μM HOIP(1-480) (**f**), 26 μM HOIP(480-639) (**g**), or 13 μM HOIP(639-698) (**h**), revealing that the HOIP RING2-LDD region is responsible for the interaction of HOIP with STK4. The SEC-based assays were performed using a Superdex 200 Increase 10/300 GL column (GE Healthcare) or a Superdex 75 Increase 10/300 GL column (GE Healthcare). The "SUM" stands for the theoretical sum of the SEC profiles of the two indicated proteins. These experiments have been replicated three times.

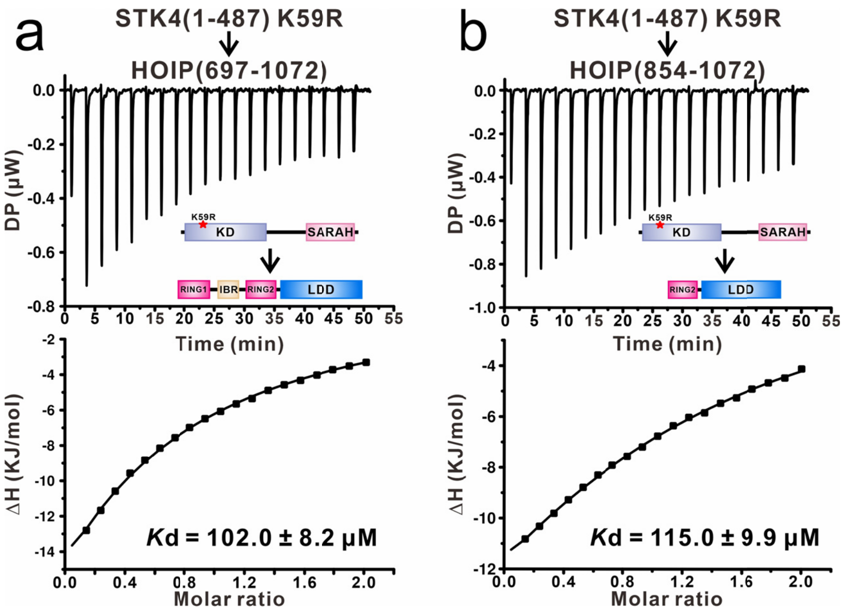

Supplementary Fig. S4. ITC-based measurements of the interactions of STK4(1-487) K59R with HOIP(697-1072) and HOIP(854-1072). a ITC-based measurement of

the binding affinity of STK4(1-487) K59R with HOIP(697-1072). **b** ITC-based measurement of the binding affinity of STK4(1-487) K59R with HOIP(854-1072). The *K*d errors are the fitted errors obtained from the data analysis software when using the one-site binding model to fit the ITC data; DP, differential power measured by the ITC machine; ΔH, heat change measured by the ITC machine. These experiments have been replicated three times.

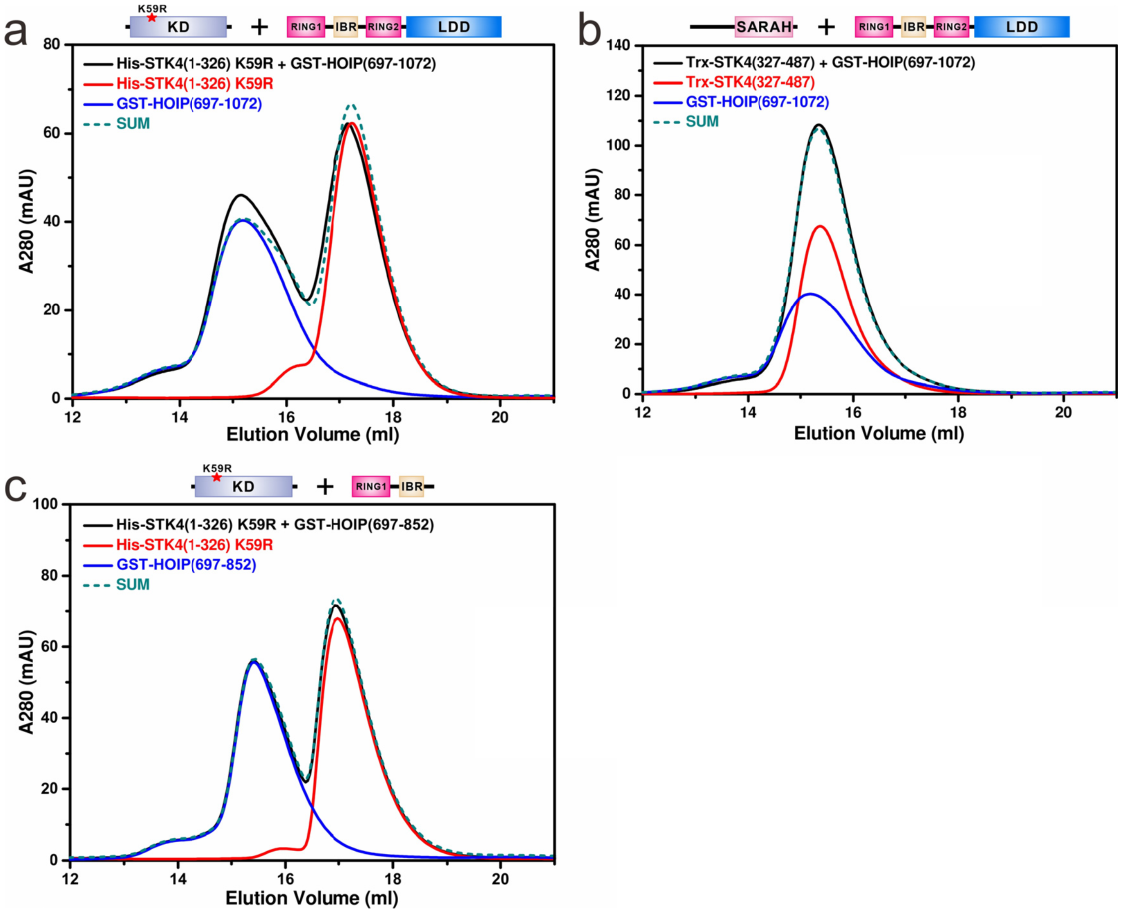

Supplementary Fig. S5. Mapping the binding regions of STK4 and HOIP using SEC-based assays. a, **b** SEC-based analyses of the interaction of 10 μM HOIP(697-1072) with 30 μM STK4(1-326) K59R (**a**), or 30 μM STK4(327-487) (**b**). **c** SEC-based analysis of the interaction of 14 μM HOIP(697-852) with 30 μM STK4(1-326) K59R. The SEC-

based assays were performed using a Superdex 200 Increase 10/300 GL column (GE Healthcare). These experiments have been replicated three times.

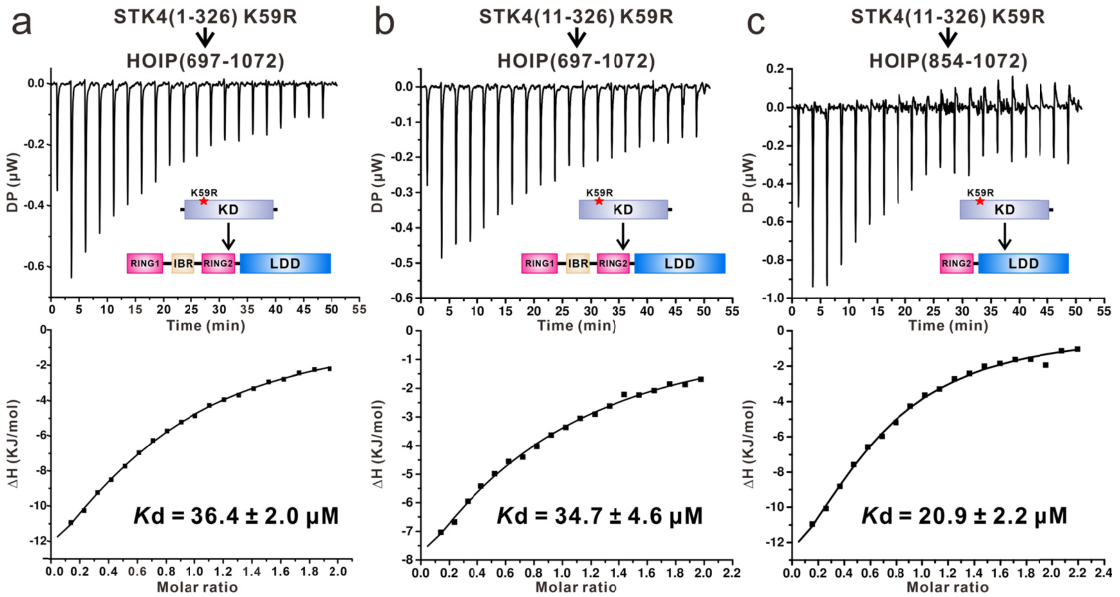

Supplementary Fig. S6. ITC-based measurements of the interactions between different HOIP and STK4 fragments. a, **b** ITC-based measurement of the binding affinity of HOIP(697-1072) with STK4(1-326) K59R (**a**), or STK4(11-326) K59R (**b**). **c** ITC-based measurement of the binding affinity of HOIP(854-1072) with STK4(11-326) K59R. The *K*d errors are the fitted errors obtained from the data analysis software when using the one-site binding model to fit the ITC data; DP, differential power measured by the ITC machine; ΔH, heat change measured by the ITC machine. These experiments have been replicated three times.

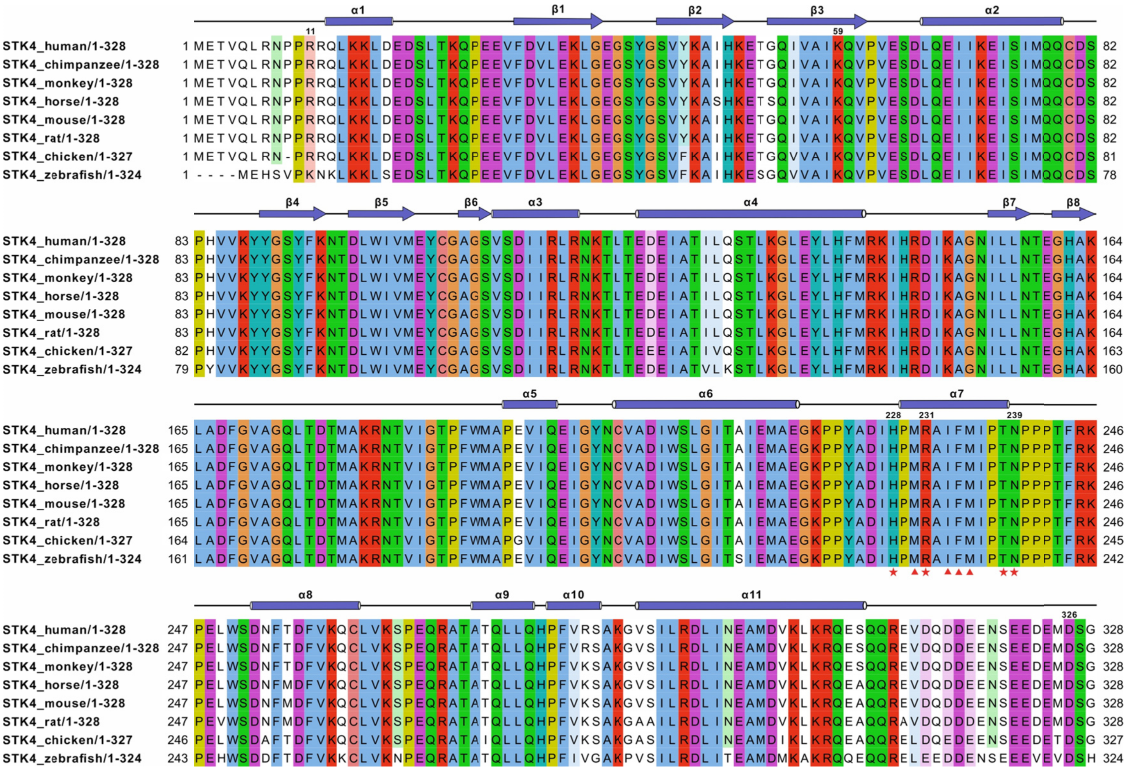

Supplementary Fig. S7. Structure-based sequence alignment analysis of STK4 from different species. In this alignment, the conserved residues are highlighted by colors using software Jalview 2.11.2.6 (http://www.jalview.org/). The binding interface residues in the STK4 kinase domain, which are important for the interaction of STK4 with HOIP, are highlighted with red stars (polar interactions) and red triangles (hydrophobic interactions).

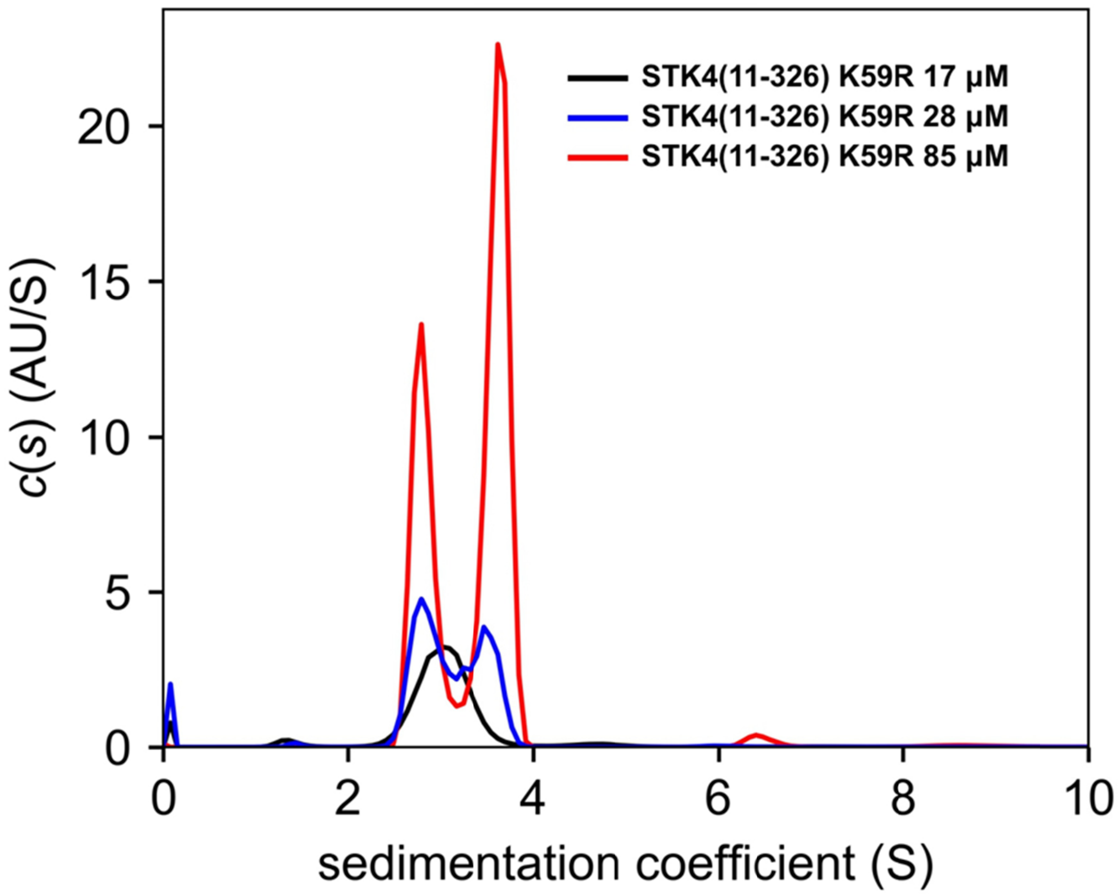

Supplementary Fig. S8. Overlay plot of the sedimentation velocity data of STK4(11-326) K59R under different concentrations. c(s) is the continuous sedimentation coefficient distribution extracted from experimental sedimentation profiles using method implemented in the SEDFIT software. These results demonstrated that STK4(11-326) K59R exists in a concentration-dependent monomer-dimer equilibrium state. These experiments have been replicated three times.

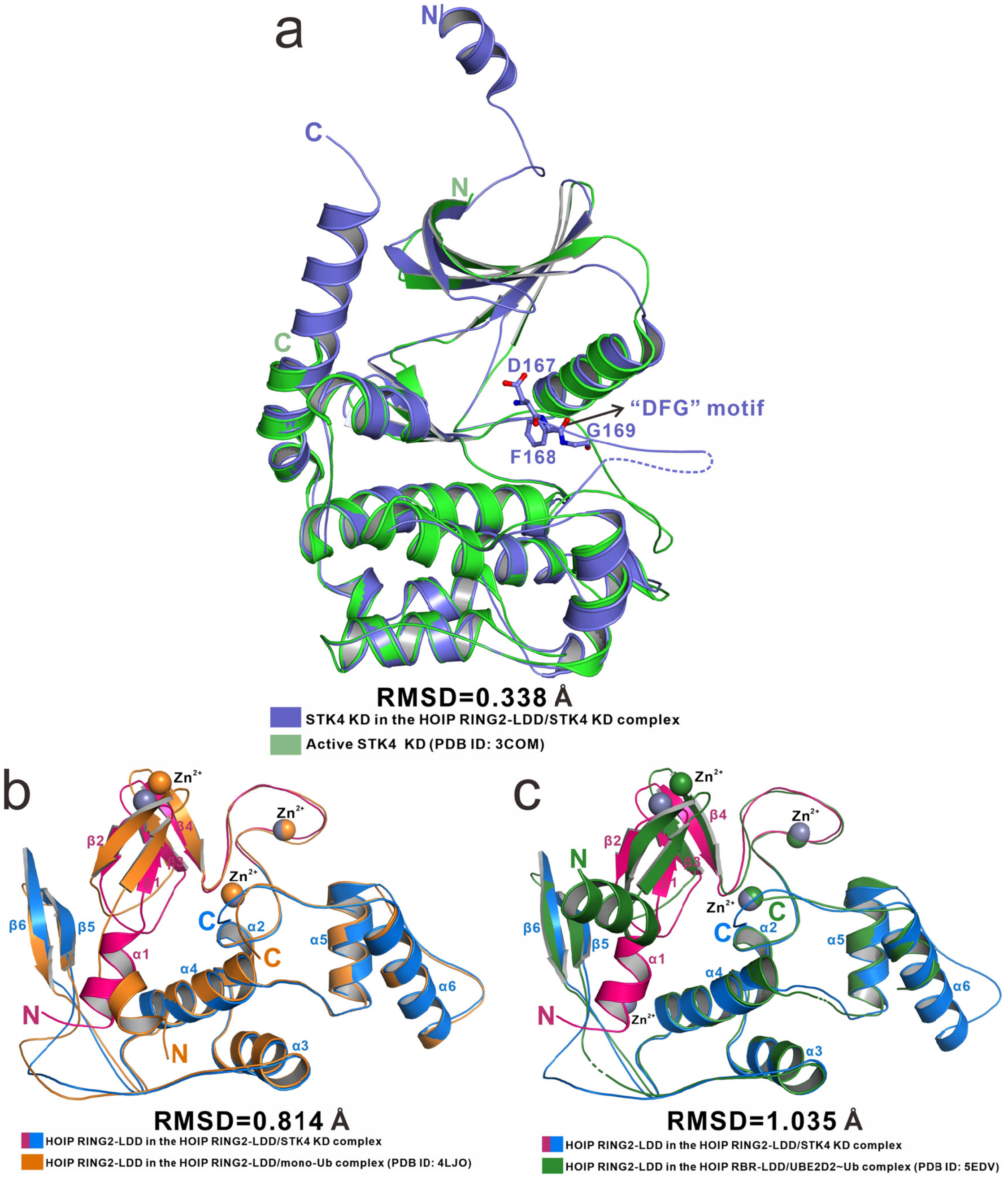

Supplementary Fig. S9. Structural analyses of STK4 KD and HOIP RING2-LDD in the STK4 KD/HOIP RING2-LDD complex. a Structural comparison analysis of the overall structures of STK4 KD in the HOIP RING2-LDD/STK4 KD complex and the active STK4 KD. In this diagram, the STK4 KD in the HOIP RING2-LDD/STK4 KD complex is colored in slate, and the active STK4 KD (PDB ID: 3COM) in green. **b**

Ribbon diagram showing the comparison of the overall structures of the HOIP RING2-LDD in the HOIP RING2-LDD/STK4 KD complex (warmpink/marine) and in the HOIP RING2-LDD/mono-Ub complex (orange) (PDB ID: 4LJO). **c** Ribbon diagram showing the structural comparison of HOIP RING2-LDD in the HOIP RING2-LDD/STK4 KD complex (pink/marine) and in the HOIP RBR-LDD/UBE2D2~Ub/Ub complex (forest) (PDB ID: 5EDV).

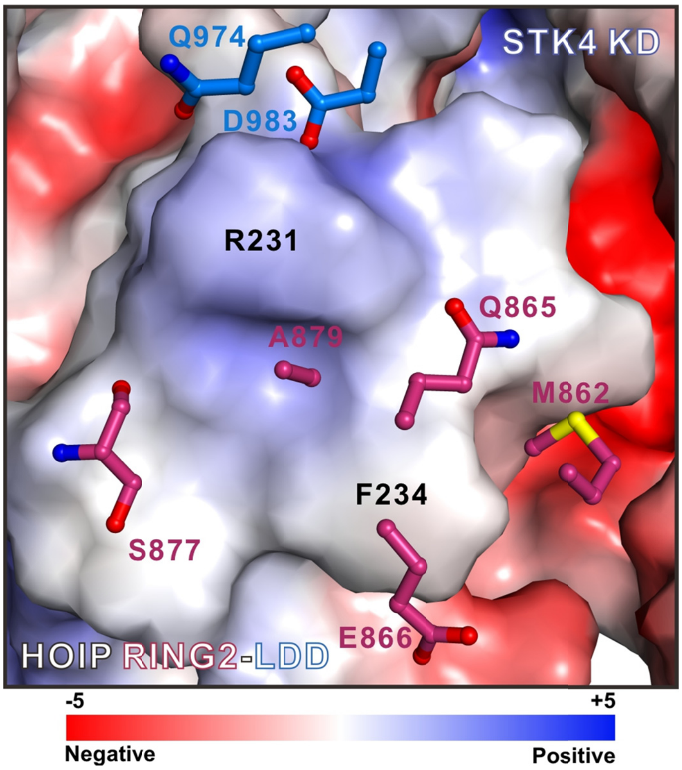

Supplementary Fig. S10. Structural analysis of the HOIP RING2-LDD/STK4 KD complex. The combined surface charge representation (contoured at ±5 kT/eV; blue/red) and the stick model showing the charge-charge interactions between HOIP RING2-LDD and STK4 KD. In this drawing, the side chains of the key interface residues of HOIP RING2-LDD are shown in the stick-ball mode, and the R231 and F234 residues of STK4 KD are also indicated.

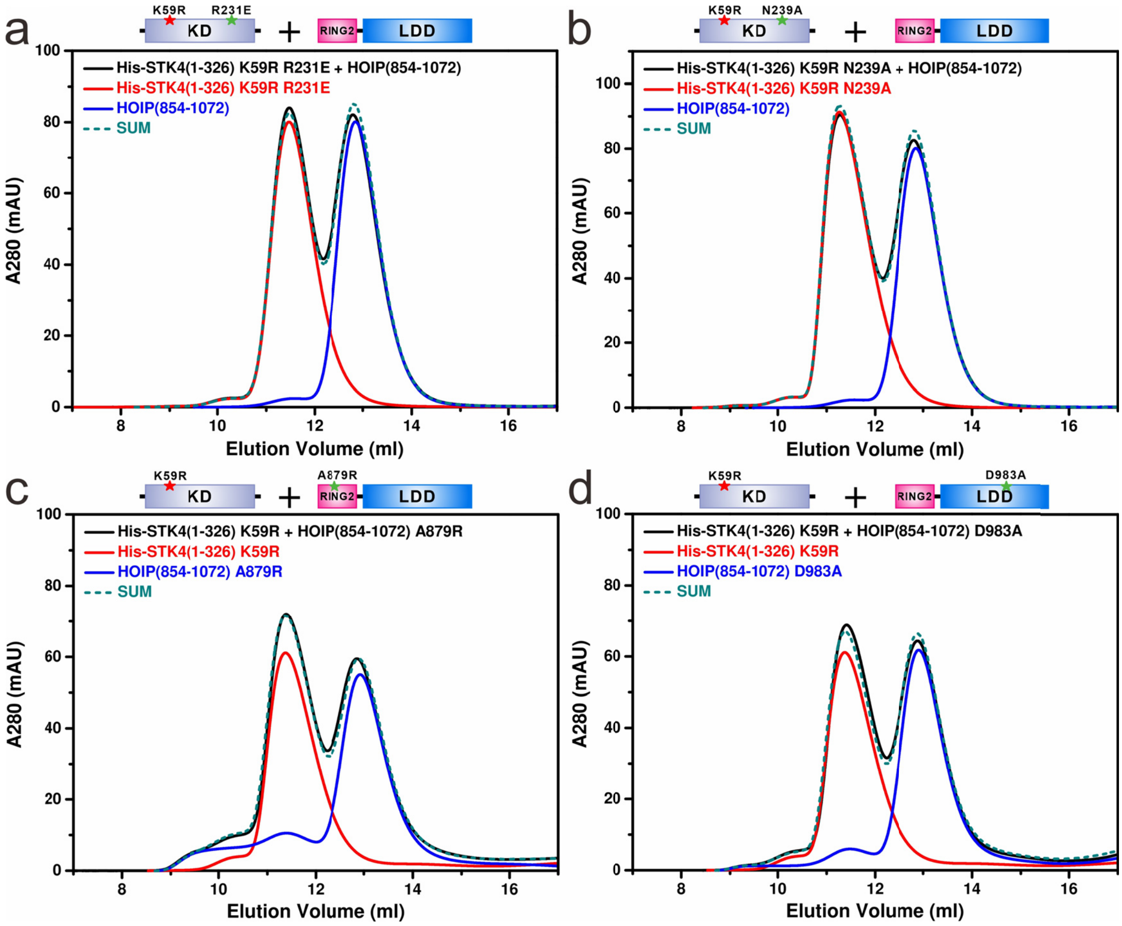

Supplementary Fig. S11. SEC-based validations of the HOIP RING2-LDD/STK4 KD complex structure by mutations of key interface residues in HOIP or STK4. a, **b** SEC-based analyses of the interactions of 43 μM HOIP(854-1072) with the 30 μM STK4(1-326) K59R/R231E mutant (**a**) and K59R/N239A mutant (**b**). **c**, **d** SEC-based analyses of the interactions of 30 μM STK4(1-326) K59R with 43 μM HOIP(854-1072) A879R mutant (**c**) and D983A mutant (**d**). The SEC-based assays were performed using a Superdex 75 Increase 10/300 GL column (GE Healthcare). The "SUM" stands for the theoretical sum of the SEC profiles of the two indicated proteins. These experiments have been replicated three times.

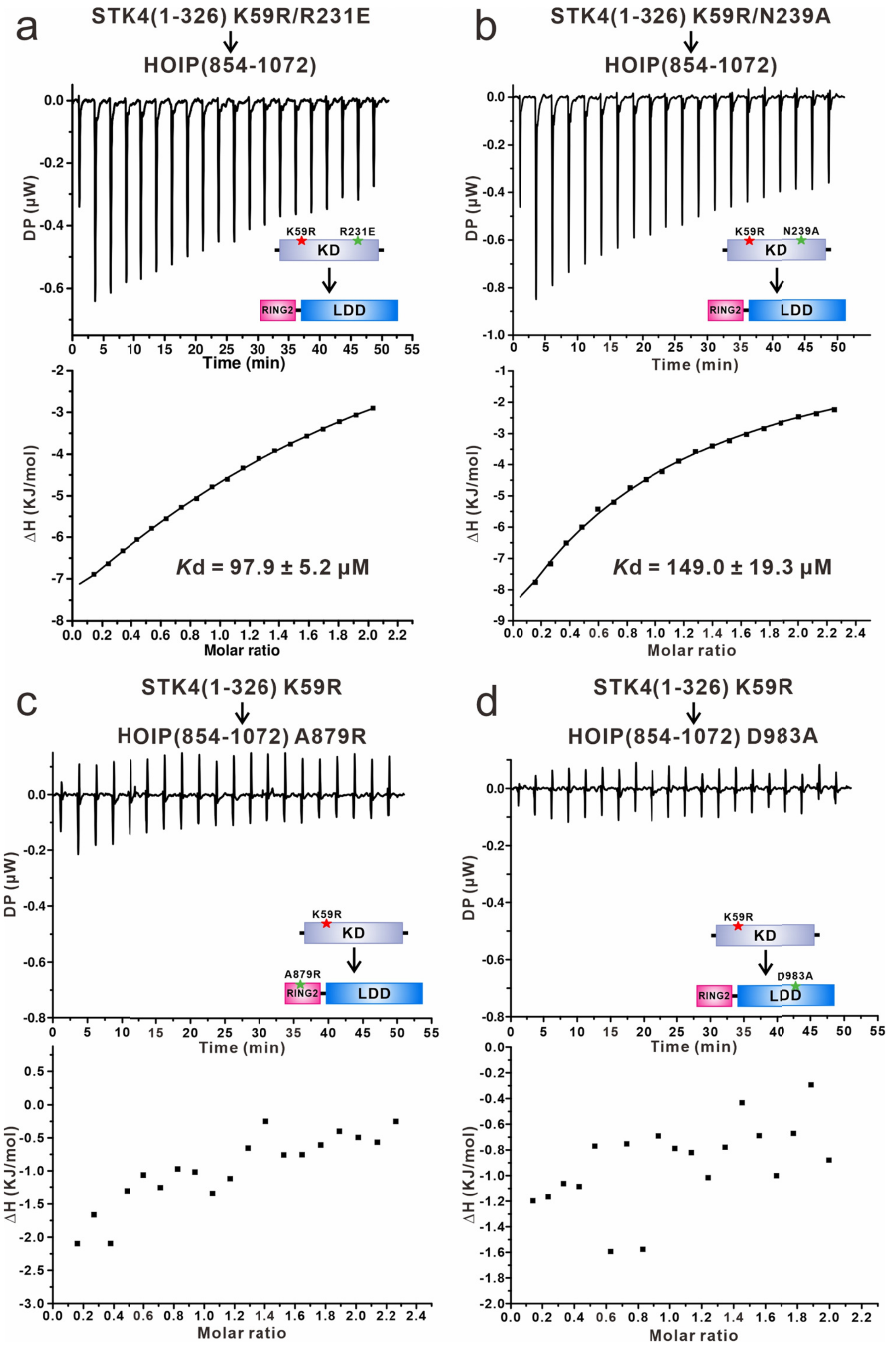

Supplementary Fig. S12. ITC-based validations of the HOIP RING2-LDD/STK4 KD complex structure by mutations of key interface residues in HOIP or STK4. a, b ITC-based measurements of the binding affinities of HOIP(854-1072) with the STK4(1-

326) K59R/R231E mutant (**a**) and K59R/N239A mutant (**b**). **c**, **d** ITC-based measurements of the binding affinities of STK4(1-326) K59R with the HOIP(854-1072) A879R mutant (**c**) and D983A mutant (**d**). These experiments have been replicated three times.

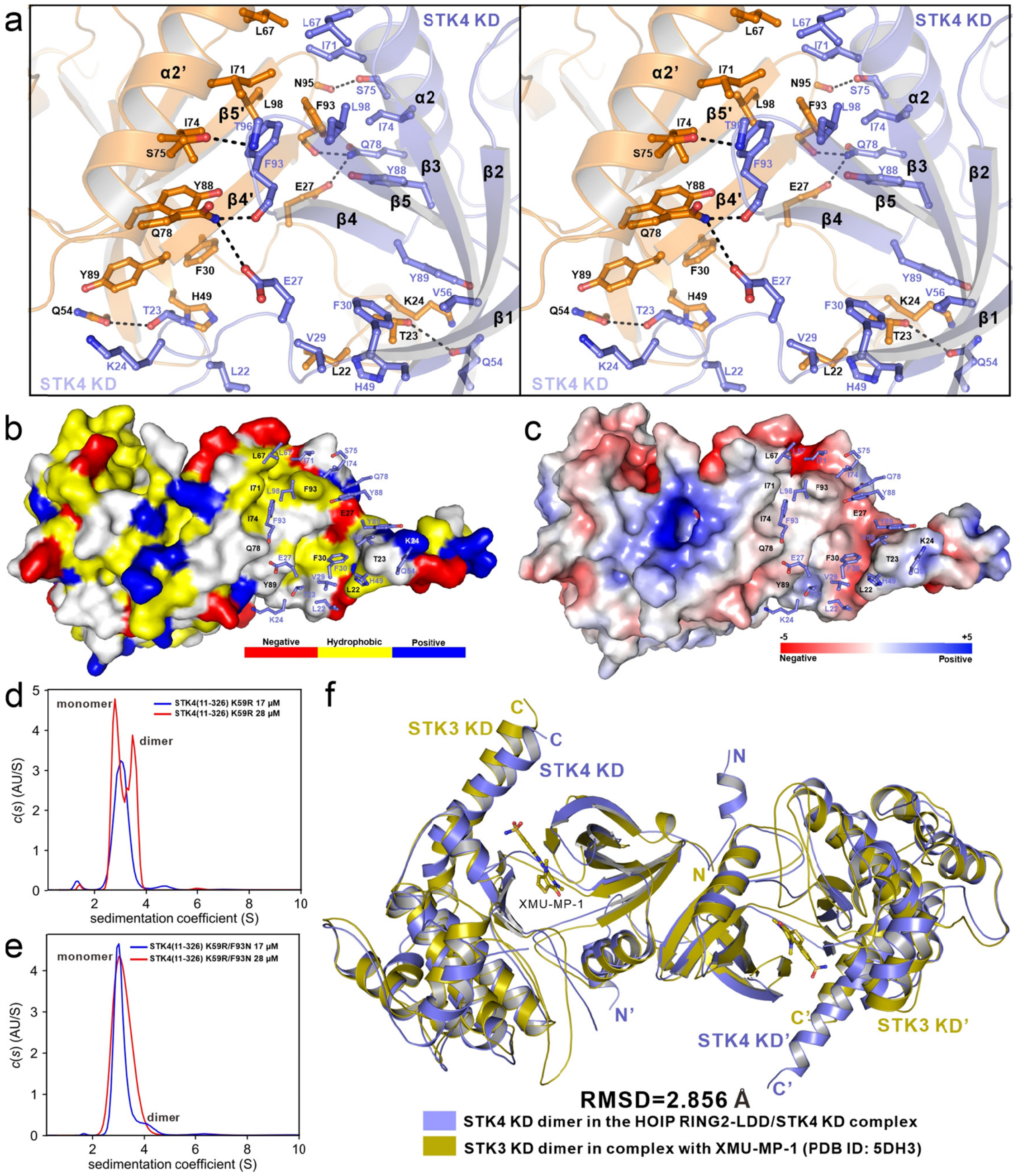

Supplementary Fig. S13. Structural and biochemical analyses of the dimerization property of STK4 KD. a Stereo view of the ribbon-stick model showing the dimerization interface of the STK4 KD dimer. In this drawing, the relevant side chains as well as backbone groups of the key binding interface residues are shown in the stick-ball mode, and the related hydrogen bonds involved in the binding are shown as dotted lines.

b The combined surface representation and the ribbon-stick model showing the hydrophobic binding surface of the STK4 KD dimer. In this drawing, one STK4 KD molecule is showed in the surface representation colored by amino acid types. Specifically, the hydrophobic amino acid residues in the surface model of STK4 are drawn in yellow, the positively charged residues in blue, the negatively charged residues in red, and the uncharged polar residues in gray. The other STK4 KD molecule is only shown the key residues located in the dimer interface. **c** The combined surface charge representation and the ribbon-stick model showing the charge-charge interactions of two STK4 molecules. **d**, **e** Overlay plots of the sedimentation velocity data of STK4(11-326) K59R (**d**) or STK4(11-326) K59R/F93N (**e**) under different concentrations, indicating that the F93N mutation can disrupt the dimer formation of STK4 KD, especially under a low concentration. These experiments have been replicated three times. **f** Ribbon diagram showing the comparison of the overall structures of the STK4 KD dimer in the HOIP RING2-LDD/STK4 KD complex and the STK3 KD dimer in the STK3 KD/XMU-MP-1 complex (PDB ID: 5DH3).

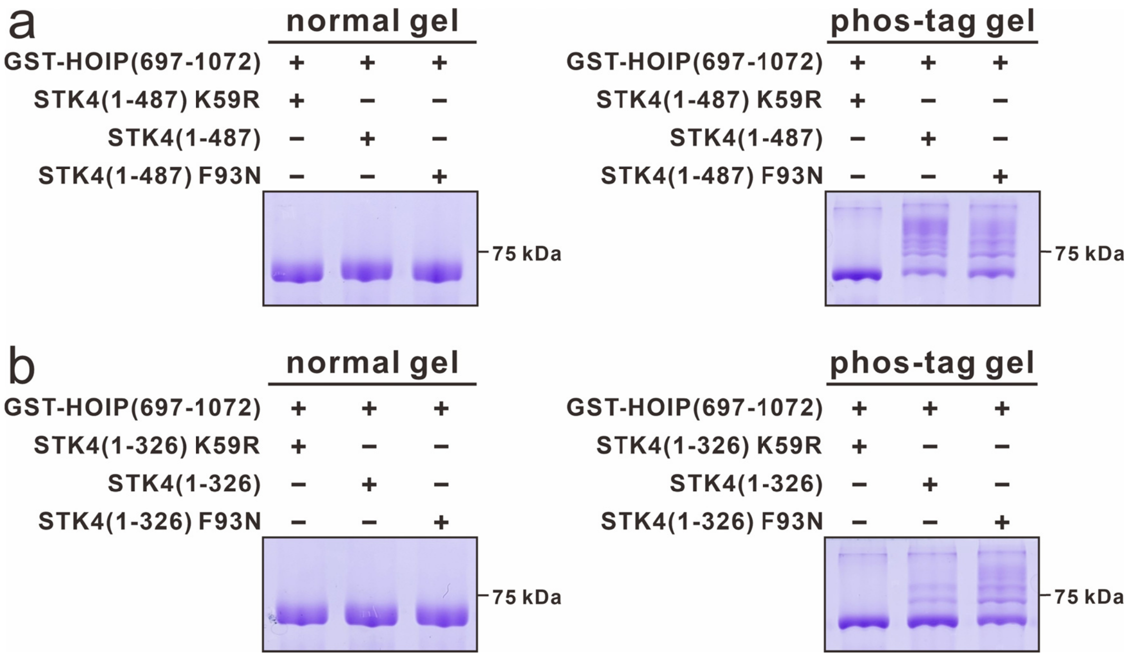

Supplementary Fig. S14. The dimerization of STK4 KD is not essential for the phosphorylation of HOIP mediated by STK4. a, **b** *In vitro* phosphorylation assays showing that HOIP(697-1072) can be well phosphorylated by the wild-type and the F93N mutant of full-length STK4 (**a**) or STK4 KD (**b**). The gel in the left panel is a normal SDS-PAGE gel, while the right panel is a phos-tag gel. These experiments have been replicated three times.

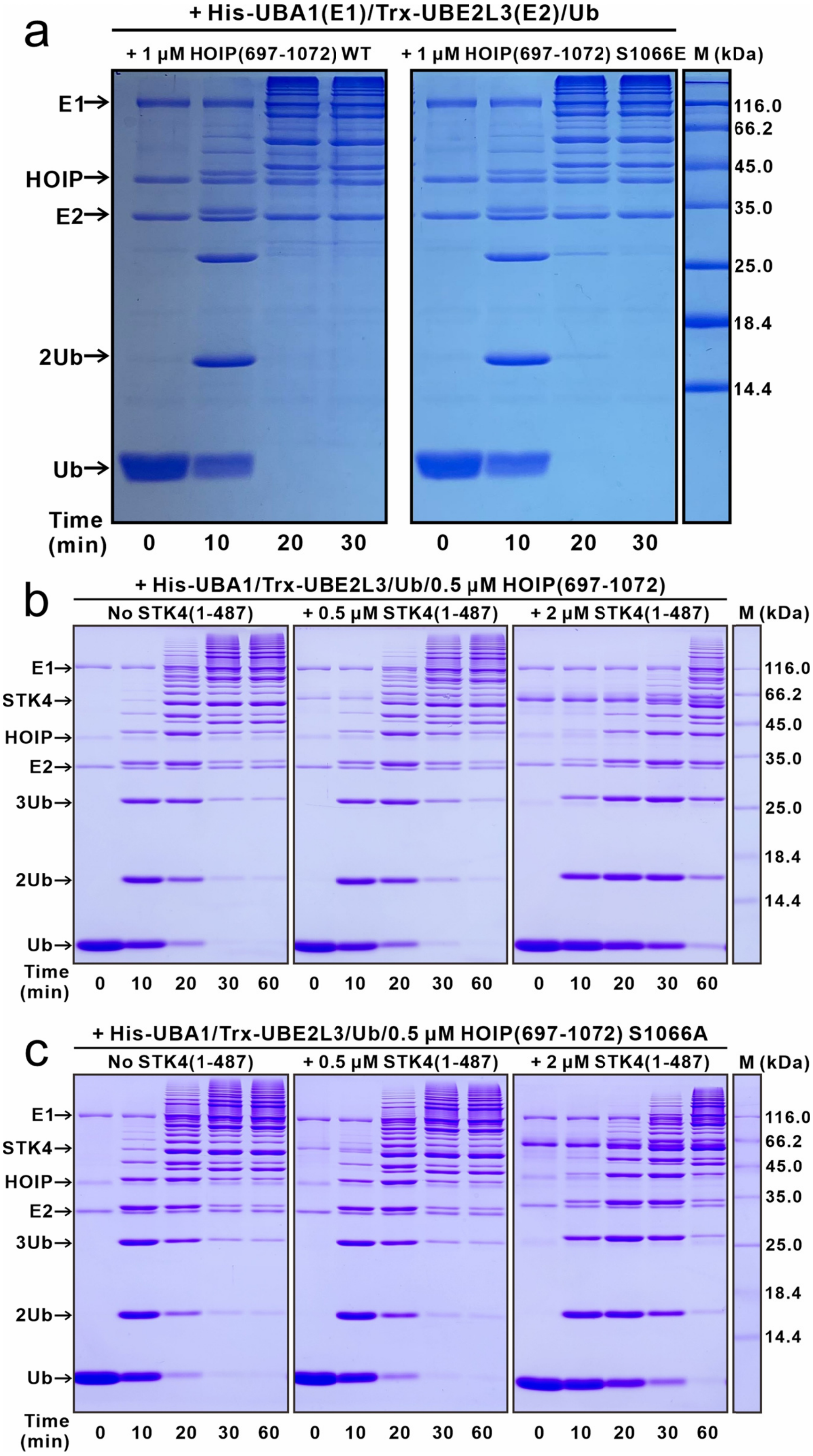

Supplementary Fig. S15. HOIP S1066 residue is not the key phosphorylation site mediated by STK4 for inhibiting the E3 activity of HOIP. a *In vitro* linear

ubiquitination assays showing the abilities of HOIP(697-1072) and the HOIP(697-1072) S1066E mutant to assemble linear ubiquitin chains. Notably, the phosphomimic S1066E mutant of HOIP(697-1072) has no obvious effect on the E3 activity of HOIP. **b**, **c** *In vitro* ubiquitination assays in the presence of different amounts of STK4 showing the E3 activity of the wild type HOIP(697-1072) (**b**) or the HOIP(697-1072) S1066A mutant (**c**) for assembling linear ubiquitin chains. These results indicated that the E3 activity of HOIP S1066A mutant still can be suppressed by STK4-mediated phosphorylation. The experiments depicted in this figure have been replicated three times.

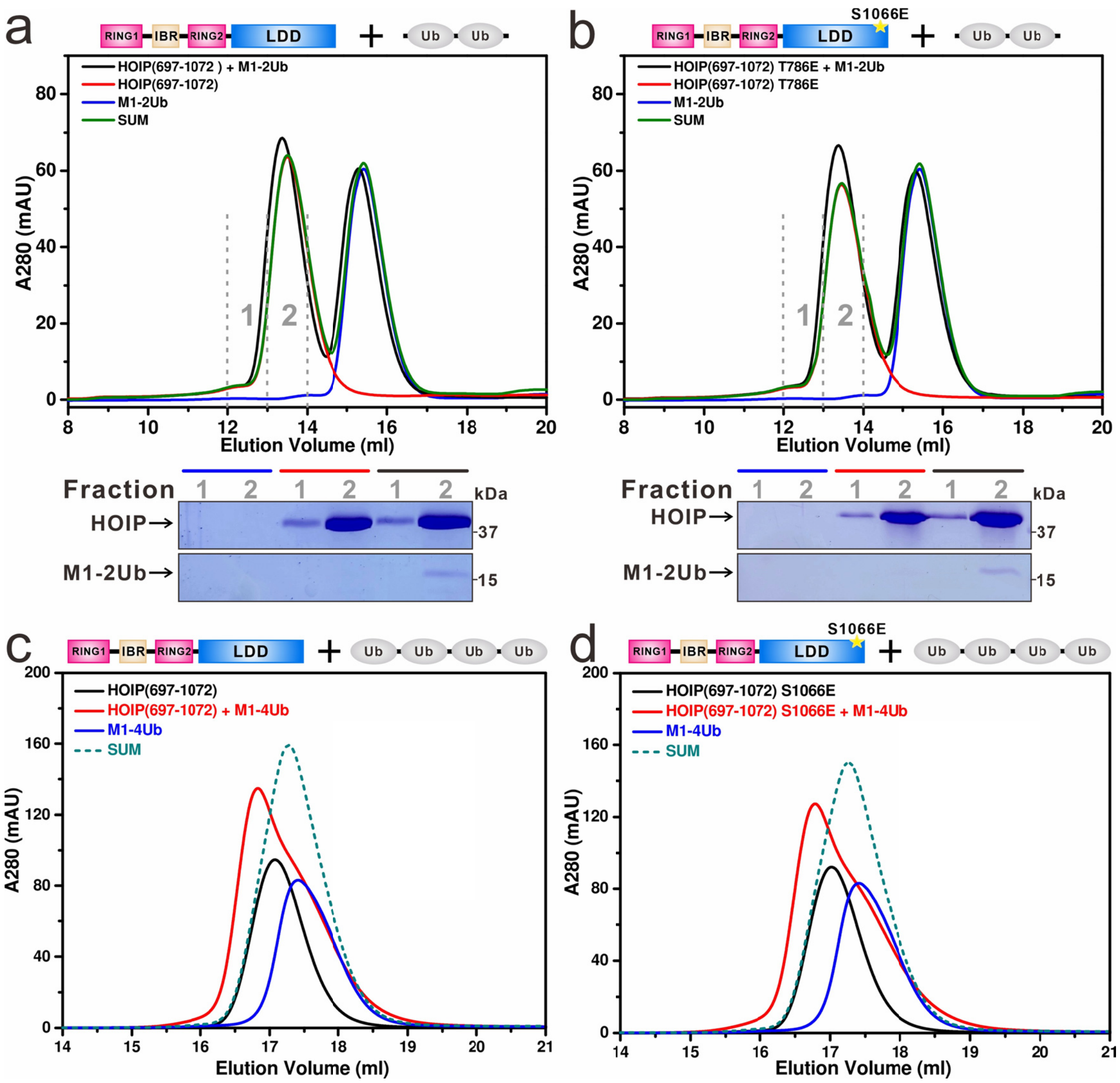

Supplementary Fig. S16. SEC-based analyses of the interactions between relevant linear ubiquitin chains and wild-type HOIP(697-1072) or its S1066E mutant. a, **b** SEC-based analyses of the interaction of 300 μM M1-2Ub with 20 μM wild-type HOIP(697-1072) (**a**), or 20 M HOIP(697-1072) S1066E mutant (**b**). The lower panels showing the SDS-PAGE combined with Coomassie blue staining analyses of the protein components of the corresponding fraction 1 and 2 collected from the SEC-based experiments. **c**, **d** SEC-based analyses of the interaction of 150 μM M1-4Ub with 20 μM wild-type HOIP(697-1072) (**c**), or 20 μM HOIP(697-1072) S1066E mutant (**d**). The SEC-based assays were performed using a Superdex 200 Increase 10/300 GL column

(GE Healthcare). The “SUM” stands for the theoretical sum of the SEC profiles of the two indicated proteins. These experiments have been replicated three times.

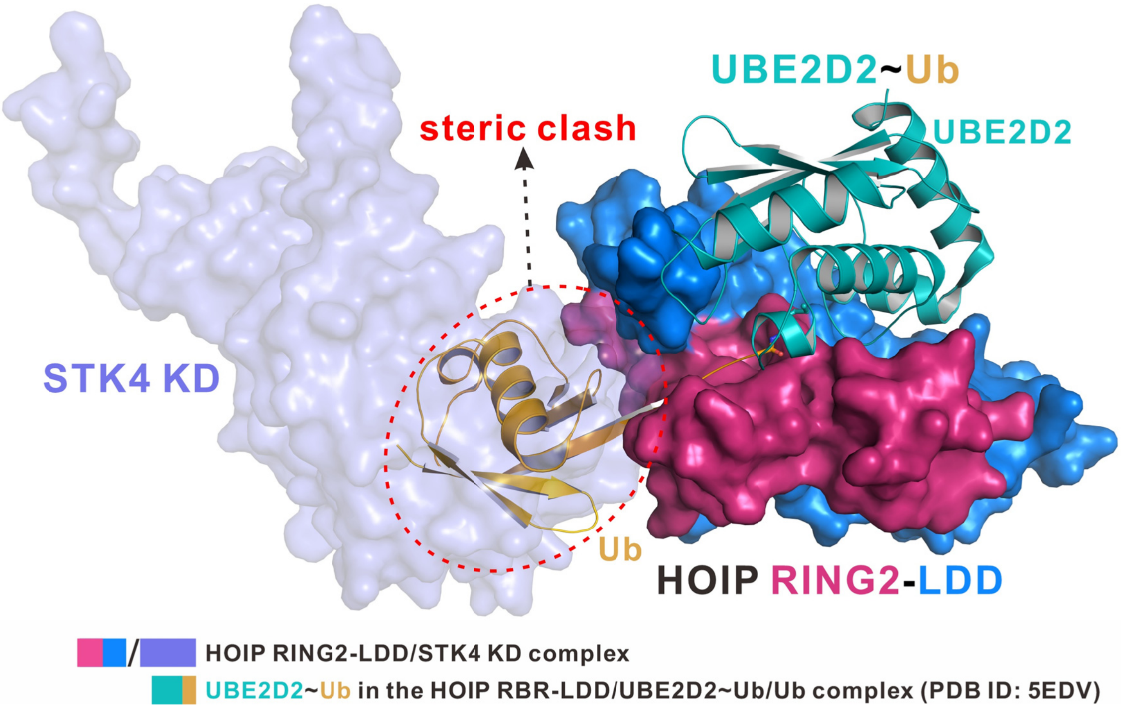

Supplementary Fig. S17. The binding of STK4 KD to the RING2-LDD of HOIP hinders the binding of the donor Ub from the E2~Ub conjugate to HOIP due to the potential steric exclusion. Combined surface representation and the ribbon-stick mode showing the structural comparison of the STK4 KD/HOIP RING2-LDD complex and the RING2-LDD/UBE2D2~Ub portion in the HOIP RBR-LDD/UBE2D2~Ub/Ub complex (PDB ID: 5EDV) by aligning the selected RING2-LDD modules in these two complex structures. In this drawing, the STK4 KD/HOIP RING2-LDD complex is shown in the surface representation, while the UBE2D2~Ub conjugate is shown in the ribbon-stick mode.

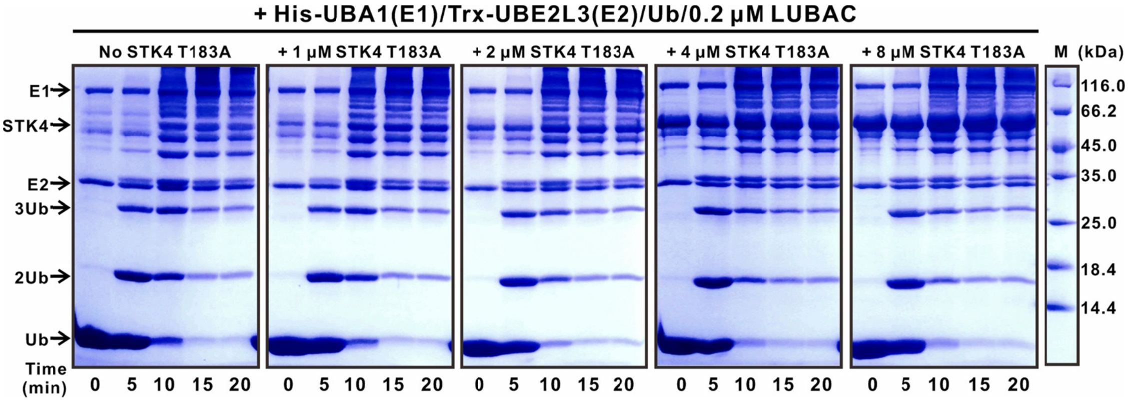

Supplementary Fig. S18. ***In vitro*** **linear ubiquitination assays showing the kinase-dead T183A mutant of STK4 is unable to suppress the catalytic activity of LUBAC.** The LUABC complex used in these assays is composed of HOIP(480-1072), the full-length HOIL-1L C460A mutant and the full-length Sharpin. The experiments depicted in this figure have been replicated three times.

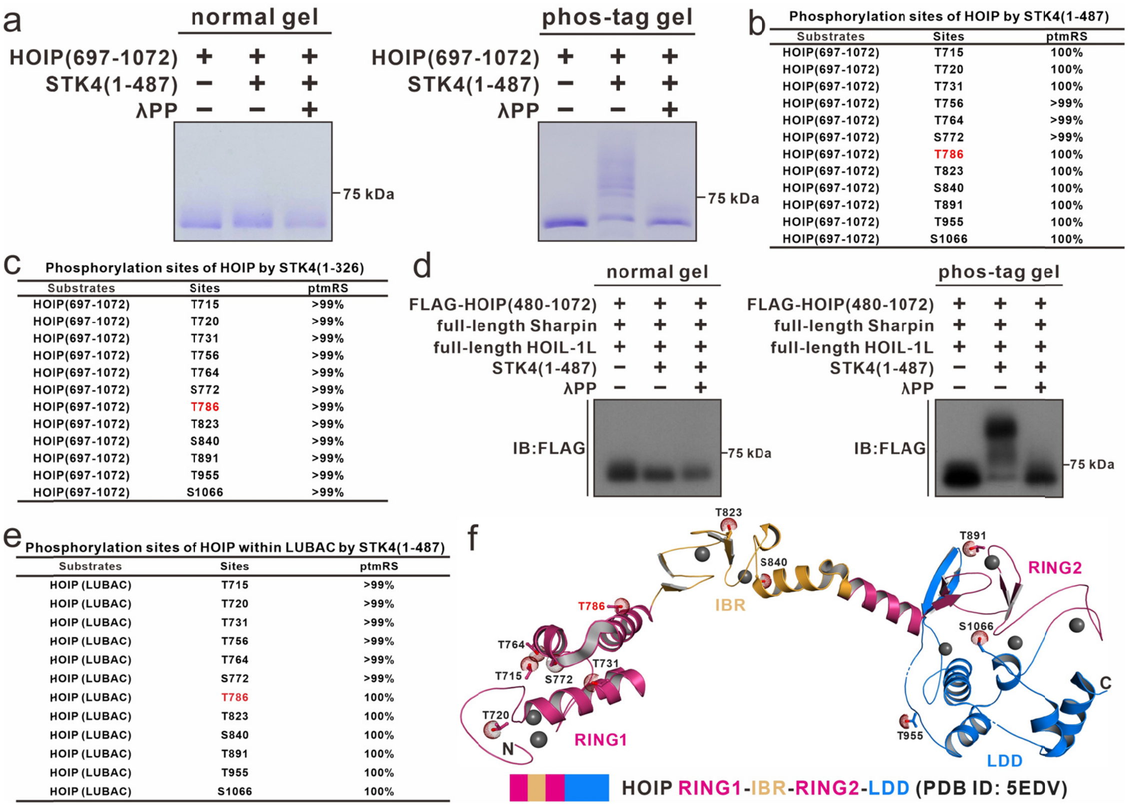

Supplementary Fig. S19. STK4-mediated phosphorylation sites of HOIP(697-1072) detected by mass spectrometry analysis. a *In vitro* phosphorylation assays showing that HOIP(697-1072) can be phosphorylated by the full-length STK4. The gel in the left panel is a normal SDS-PAGE gel, while the right panel is a phos-tag gel. These experiments have been replicated three times. **b** The summary of the potential phosphorylation sites of HOIP(697-1072) mediated by the full-length STK4 detected by mass spectrometry. "ptmRS" stands for the best site probabilities. **c** The summary of the potential phosphorylation sites of HOIP(697-1072) mediated by STK4(1-326) detected by mass spectrometry. "ptmRS" stands for the best site probabilities. **d** *In vitro* phosphorylation assays showing that the FLAG-HOIP(480-1072) subunit in the relevant LUBAC complex can be phosphorylated by the full-length STK4. **e** The summary of the STK4-mediated 12 phosphorylation sites located in the RBR-LDD region of HOIP(480-1072) within the

LUBAC complex in panel **d** detected by mass spectrometry. “ptmRS” stands for the best site probabilities. **f** Cartoon diagram combined with the stick-ball model of HOIP RING1-IBR-RING2-LDD (PDB ID: 5EDV) showing the potential phosphorylation sites detected by our mass spectrometry analysis. These potential phosphorylation residues are highlighted in the stick-ball model.

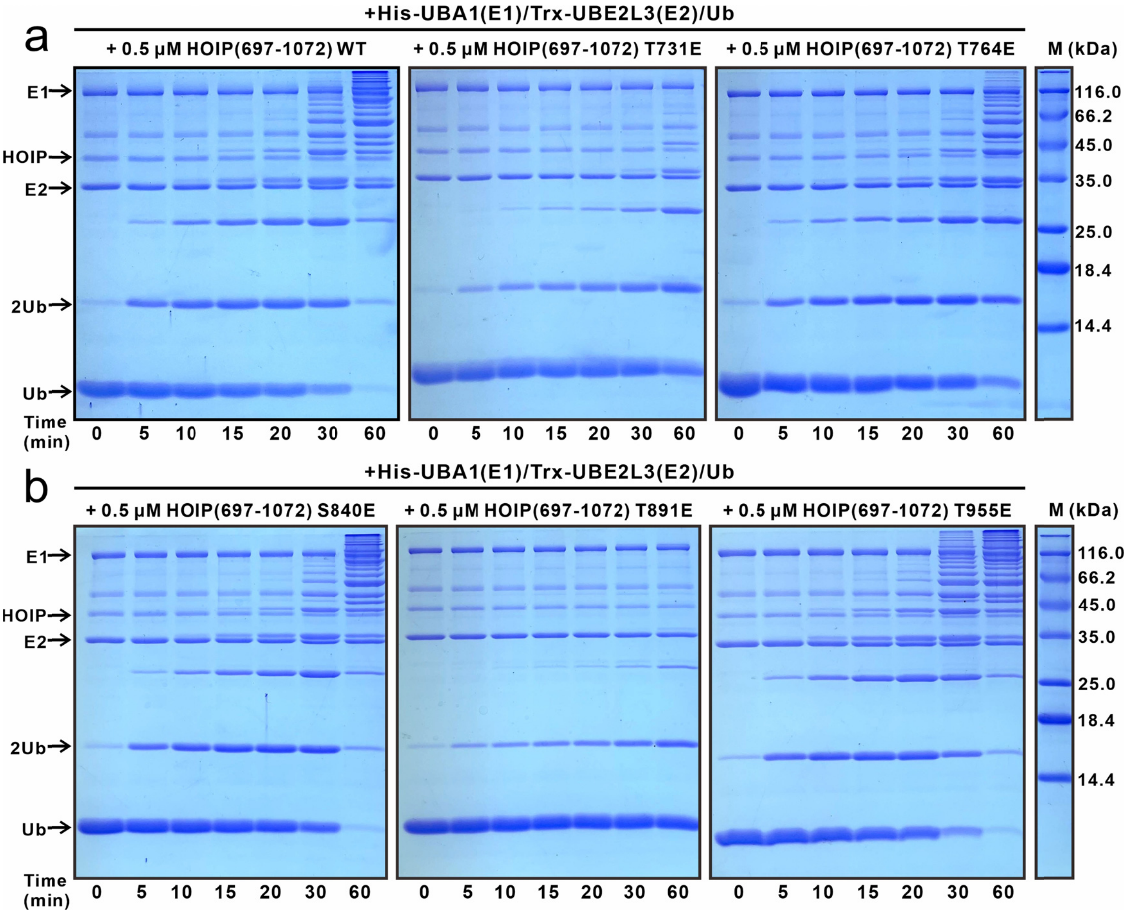

Supplementary Fig. S20. ***In vitro*** **ubiquitination assays showing the abilities of relevant phosphomimic HOIP variants to assemble linear ubiquitin chains. a** *In vitro* linear ubiquitination assays showing the abilities of the wild-type HOIP(697-1072), the phosphomimic HOIP(697-1072) T731E and T764E mutants to assemble linear ubiquitin chains. Notably, the left panel shows the same gel as that in the left panel of **Fig. 3*F*** only

at a different contrast level. **b** *In vitro* linear ubiquitination assays showing the abilities of the phosphomimic HOIP(697-1072) S840E, T891E, and T955E mutants to assemble linear ubiquitin chains. Notably, the phosphomimic T731E and T891E mutations of HOIP significantly reduce the E3 activity of HOIP, while the T764E mutation of HOIP weakens the E3 activity of HOIP. The experiments depicted in this figure have been replicated three times.

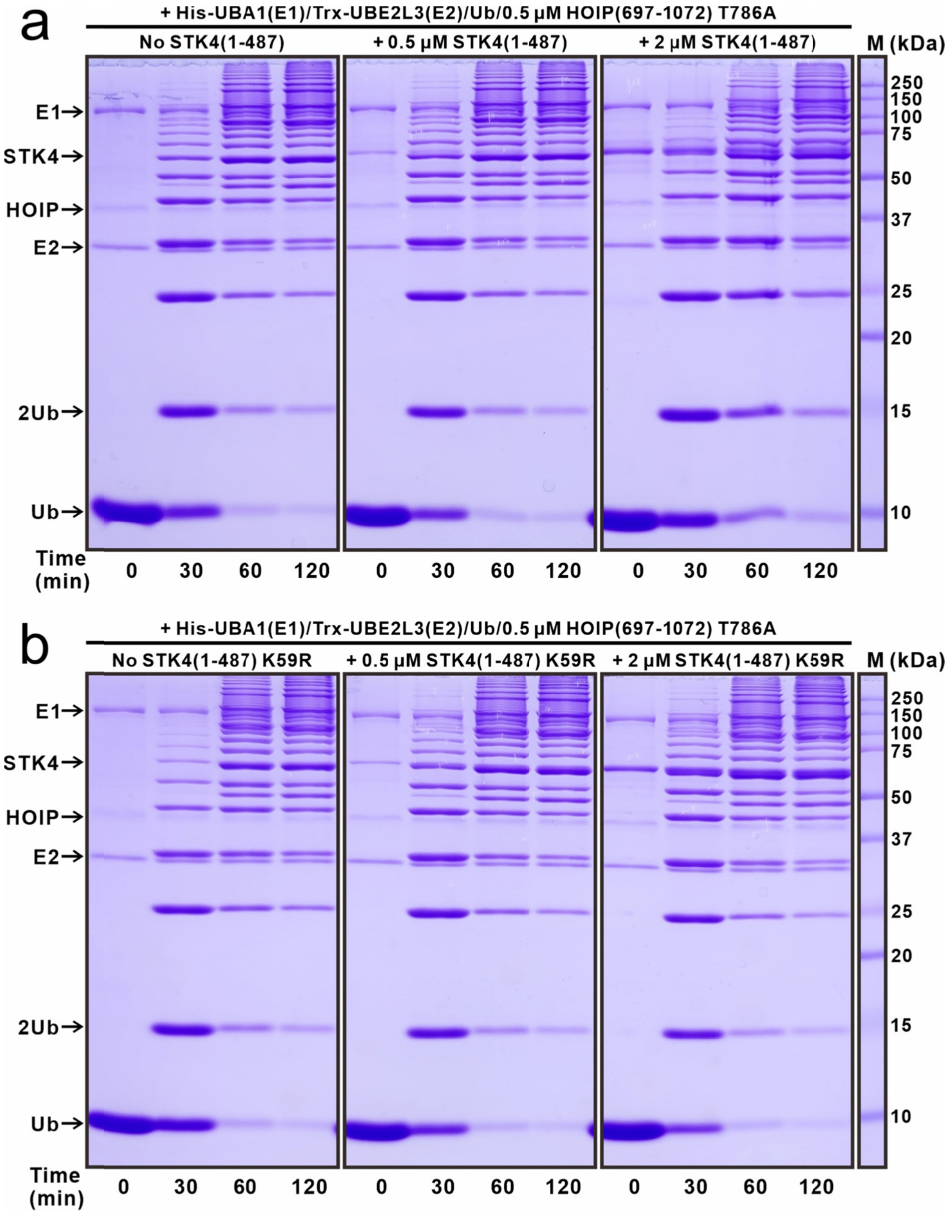

Supplementary Fig. S21. The E3 activity of the HOIP T786A mutant is unable to be effectively inhibited by STK4. a, **b** *In vitro* ubiquitination assays using the HOIP(697-1072) T786A mutant in the presence of the wild type STK4(1-487) (**a**), or the kinase-dead STK4(1-487) K59R mutant (**b**) showing that the E3 activity of HOIP(697-1072) T786A is unable to be effectively suppressed by STK4-mediated phosphorylation. The experiments depicted in this figure have been replicated three times.

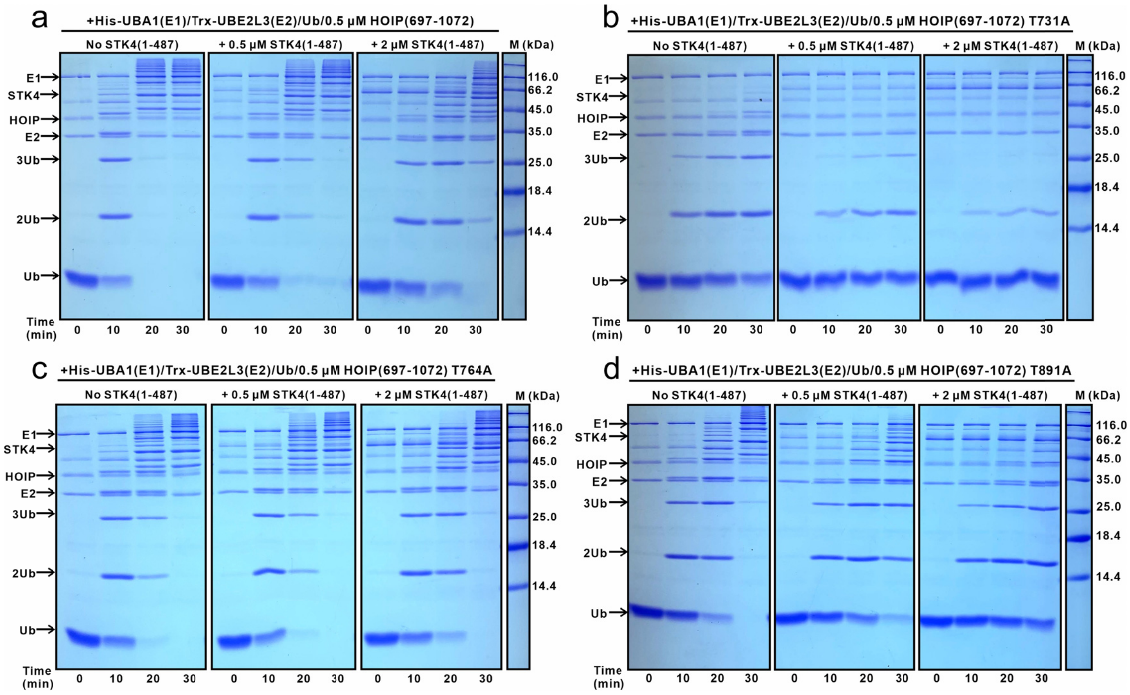

Supplementary Fig. S22. The E3 activities of HOIP T731A, T764A and T891A mutants can be suppressed by STK4-mediated phosphorylation. a-d *In vitro* ubiquitination assays in the presence of different amounts of STK4 showing that the E3 activities of the wild type HOIP(697-1072) (**a**), the HOIP(697-1072) T731A mutant (**b**), the HOIP(697-1072) T764A mutant (**c**), and the HOIP(697-1072) T891A mutant (**d**) can be well suppressed by STK4-mediated phosphorylation. The experiments depicted in this figure have been replicated three times.

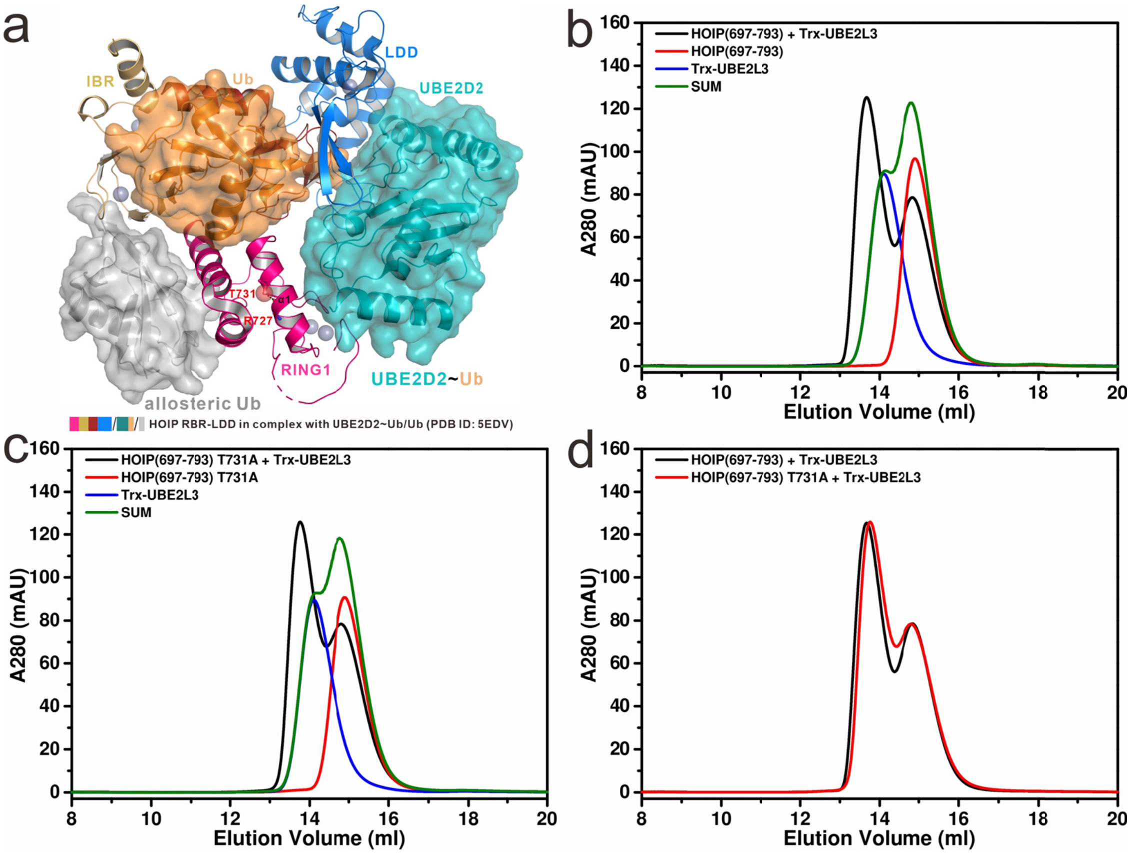

Supplementary Fig. S23. Structural and biochemical analyses of the HOIP T731A mutation. **a** Combined surface representation and the ribbon-stick-sphere mode showing the local structural arrangement of HOIP T731 residue in the HOIP RBR-LDD/UBE2D2~Ub/Ub complex structure (PDB ID: 5EDV). In this drawing, the side chain of T731 is highlighted with a red sphere, and the hydrogen bond involved in the interaction of T731 and R727 is shown as a dotted line. **b**, **c** SEC-based analyses of the interaction of 30 μM Trx-UBE2L3 with 120 μM wild-type HOIP(697-793) (**b**), or 120 μM HOIP(697-793) T731A mutant (**c**). **d** SEC-based analyses of the mixed sample from panel **b** and **c**. The SEC-based assays were performed using a Superdex 75 10/300 GL column (GE Healthcare). The "SUM" stands for the theoretical sum of the SEC profiles of Trx-UBE2L3 with wild-type HOIP(697-793) or HOIP(697-793) T731A mutant. These experiments have been replicated three times.

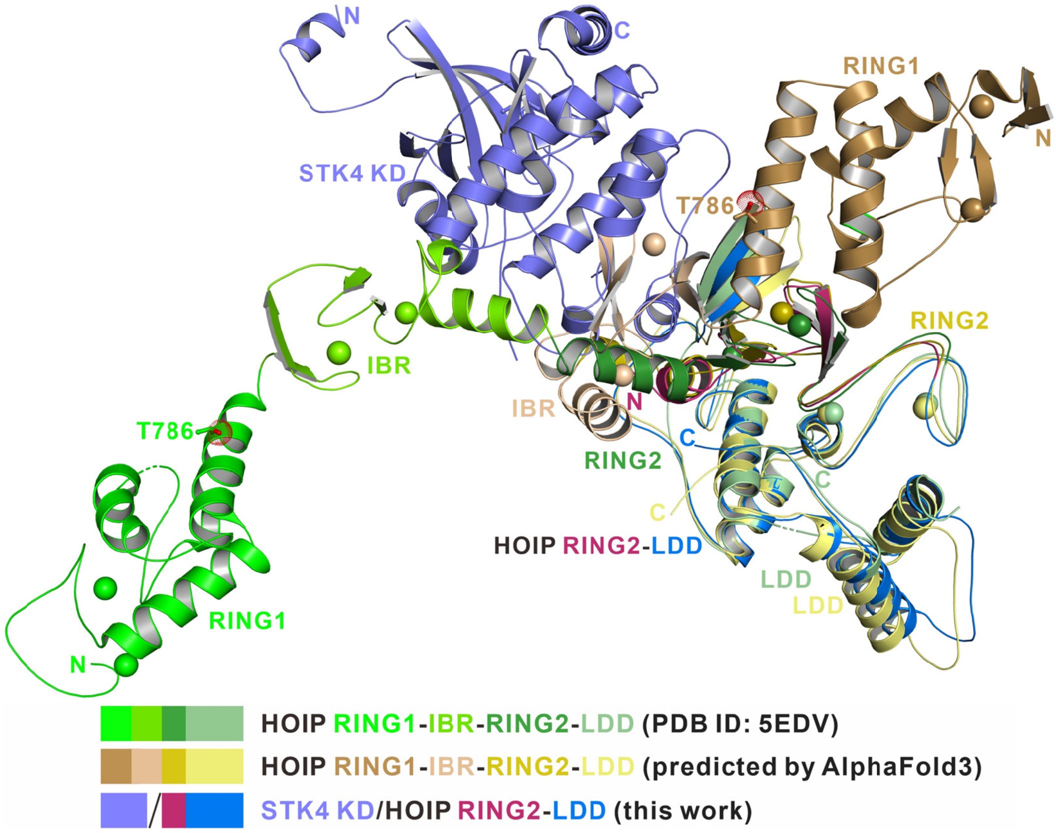

Supplementary Fig. S24. Structural modeling analysis of the accessibility of the HOIP T786 site for the STK4-mediated phosphorylation. The ribbon-stick model showing the structural comparison of HOIP RBR-LDD in the HOIP RBR-LDD/UBE2D2~Ub/Ub complex structure (PDB ID: 5EDV), the *apo*-form HOIP RBR-LDD structure predicted by AlphaFold3 and the STK4 KD/HOIP RING2-LDD complex structure solved in this study. In this analysis, the three structures are aligned together based on the overlapped RING2-LDD region, revealing that due to the flexibility of HOIP RBR-LDD region, the T786 residue of HOIP should be accessible for STK4-mediated phosphorylation.

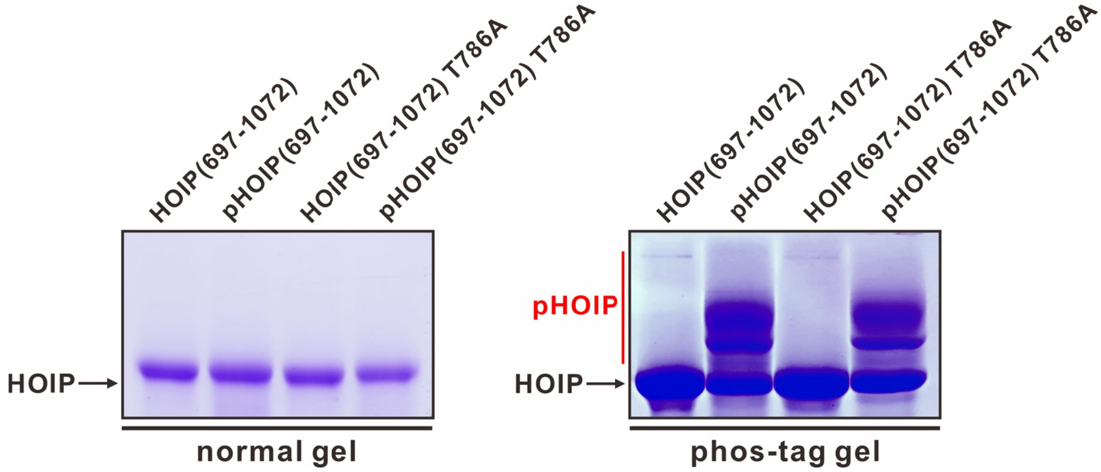

Supplementary Fig. S25. The Coomassie blue staining results showing the purified phosphorylated HOIP(697-1072) and HOIP(697-1072) T786A proteins. The gel in the left panel is normal SDS-PAGE gel, while the right one is a phos-tag gel. In this panel, pHOIP stands for phosphorylated HOIP proteins.

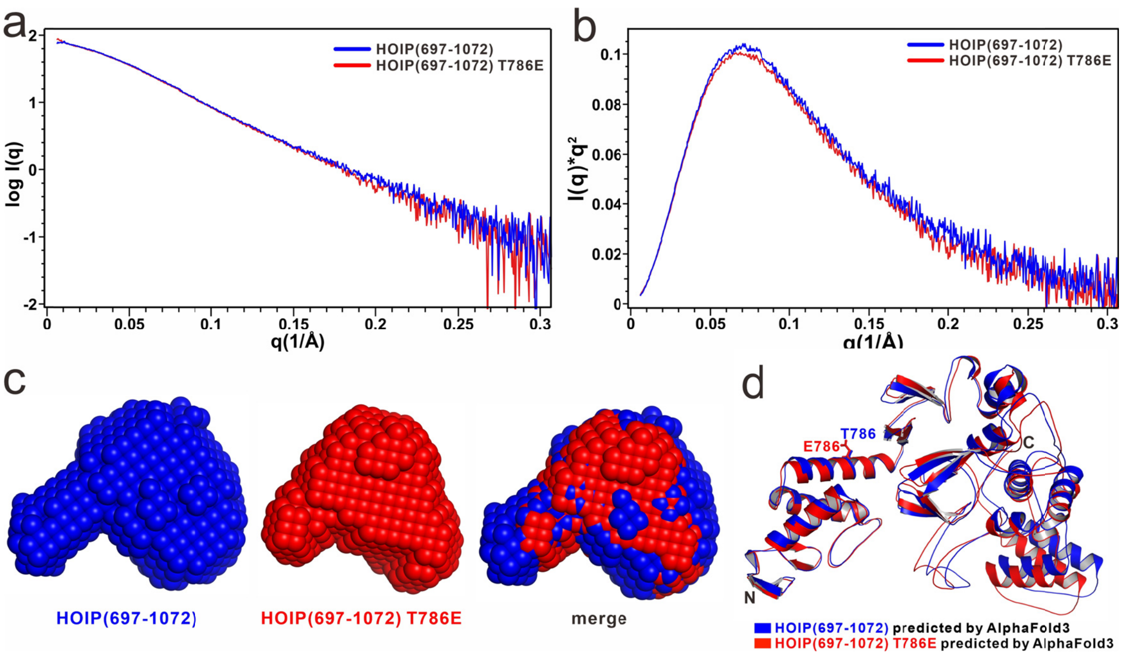

Supplementary Fig. S26. The SAXS and structural modeling analyses of the phosphomimic T786E mutation of HOIP RBR-LDD. a Standard SAXS scattering curves of the wild-type HOIP(697-1072) and the HOIP(697-1072) T786E mutant. The

displayed curves are averaged from twenty exposures and are further normalized. Data is representative of at least three observations collected at a range of concentrations. For the displayed curves, all proteins are at concentrations of 2 mg/ml. The 'I' stands for scattering intensity, q is proportional to the scattering angle ($q = 4 \sin(\theta)/\lambda$, where 2θ is the angle between the incident X-ray beam and the detector, and λ is the X-ray wavelength in Å). **b** The Kratky plot representation of the experimental solution scattering data, using the same color scheme as in panel **a**. **c** *Ab initio* models of HOIP(697-1072) and the HOIP(697-1072) T786E mutant. Each model represents the aligned, filtered average of ten independently calculated models. **d** The ribbon-stick model showing the structural comparison of the structural models of the wild-type HOIP(697-1072) and the HOIP(697-1072) T786E mutant predicted by AlphaFold3.

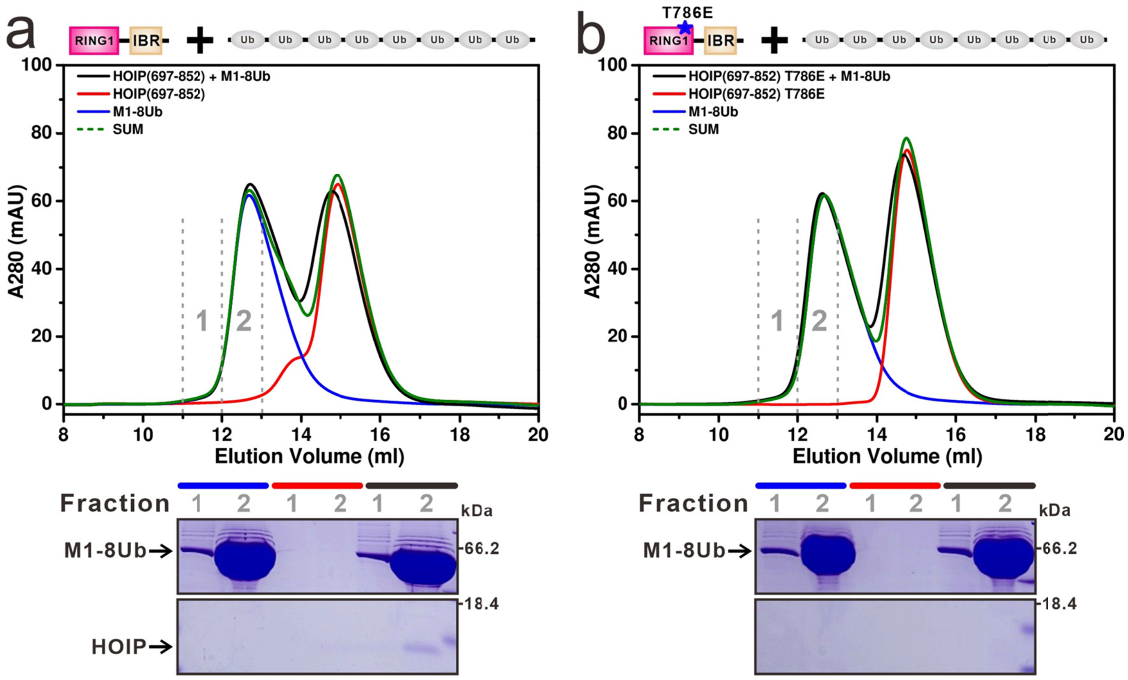

Supplementary Fig. S27. The phosphomimic T786E mutation of HOIP(697-852) can decrease the interaction between HOIP(697-852) and M1-8Ub. a, b SEC-based

analyses of the interaction of 75 μM M1-8Ub with 38 μM wild-type HOIP(697-852) (**a**), or 38 μM HOIP(697-852) T786E mutant (**b**). The lower panels showing the SDS-PAGE combined with Coomassie blue staining analyses of the protein components of the corresponding fraction 1 and 2 collected from the SEC-based experiments. The SEC-based assays were performed using a Superdex 200 Increase 10/300 GL column (GE Healthcare) and have been replicated three times. The "SUM" stands for the theoretical sum of the SEC profiles of M1-8Ub with wild-type HOIP(697-852) or HOIP(697-852) T786E mutant.

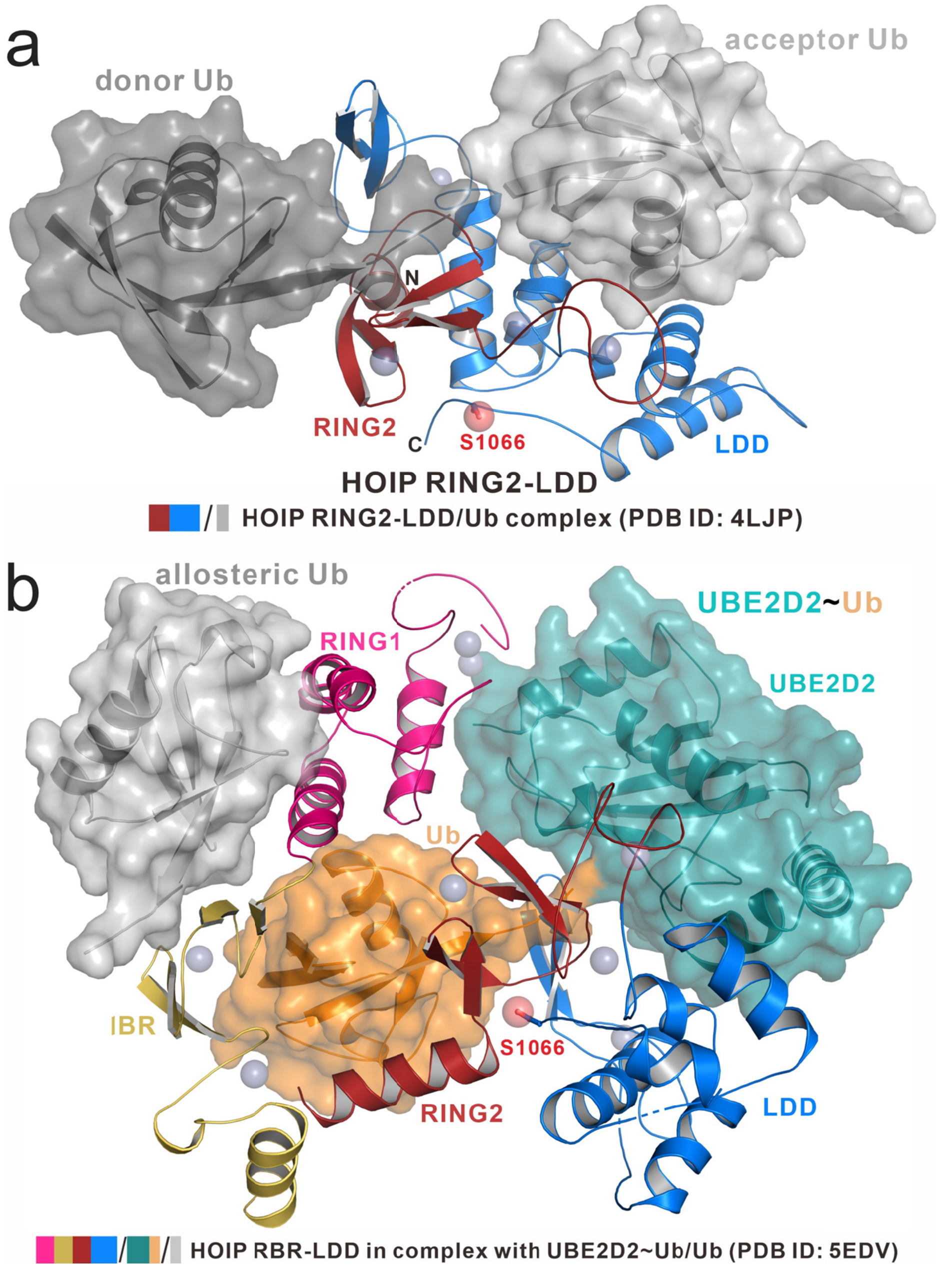

Supplementary Fig. S28. Structural analyses of the HOIP S1066 residue in the HOIP RING2-LDD/Ub complex and the HOIP RBR-LDD/UBE2D2~Ub/Ub complex. **a** Combined surface representation and the ribbon-stick mode showing the S1066 residue is located in the extreme C-terminal loop of HOIP LDD, and is far away from the donor Ub and acceptor Ub-binding sites of HOIP RING2-LDD in the HOIP RING2-LDD/Ub complex structure (PDB ID: 4LJP). In this drawing, the side chain of S1066 is

highlighted with a red sphere. **b** Combined surface representation and the ribbon-stick mode showing the HOIP S1066 residue is far away from the allosteric Ub and the E2~Ub conjugate-binding sites of HOIP RBR-LDD in the HOIP RBR-LDD/UBE2D2~Ub/Ub complex (PDB ID: 5EDV).

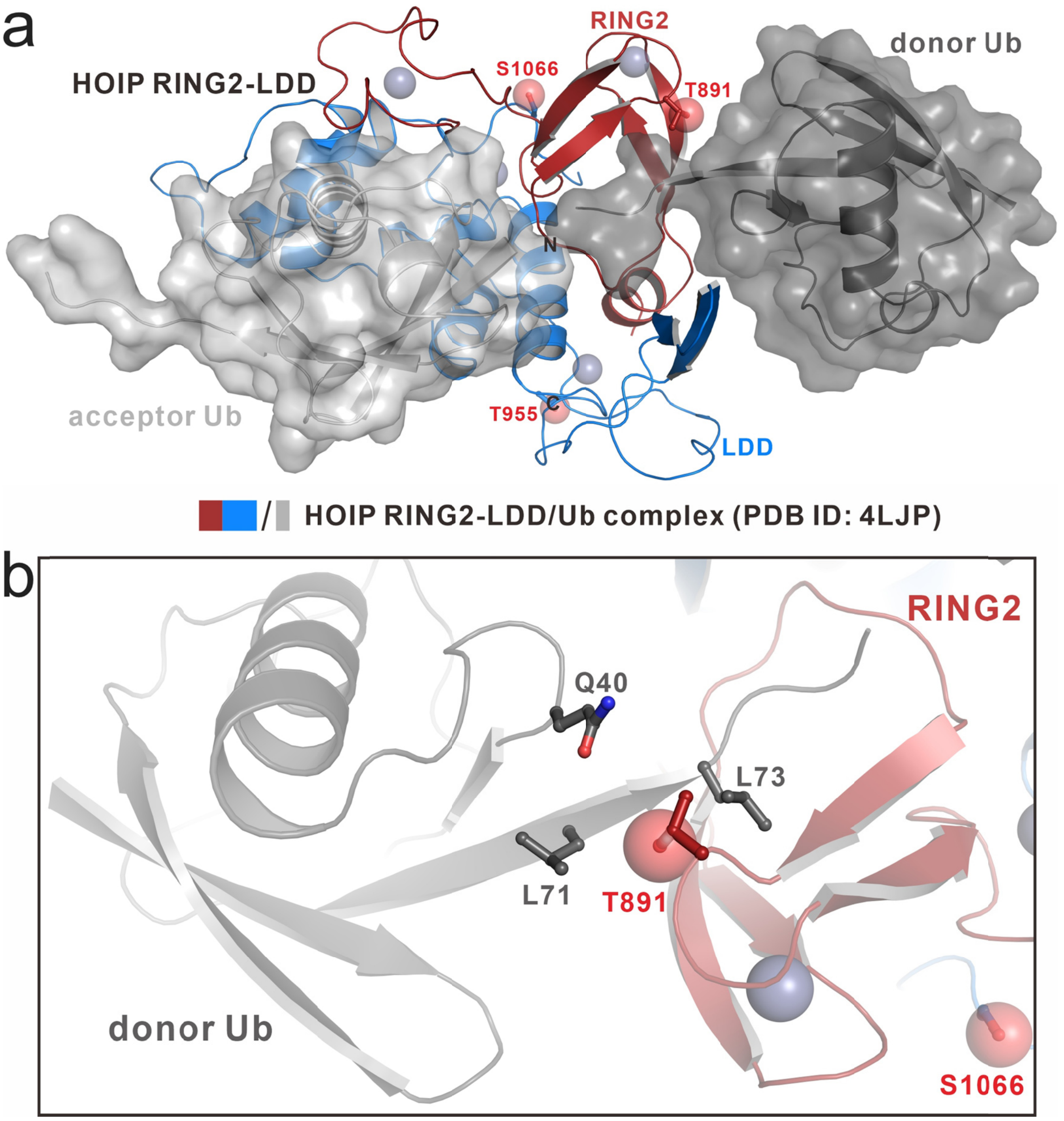

Supplementary Fig. S29. Structural analyses of the identified STK4-mediated phosphorylation sites of HOIP within the structure of the HOIP RING2-LDD/Ub

complex. a Combined surface representation and the ribbon-stick-sphere mode showing the locations of HOIP T891, T955 and S1066 residues, which can be phosphorylated by STK4, within the structure of the HOIP RING2-LDD/Ub complex (PDB ID: 4LJP). In this drawing, the side chains of HOIP T891, T955 and S1066 are highlighted with red spheres. **b** The ribbon-stick-sphere mode showing the local structural arrangement of HOIP T891 residue in the HOIP RING2-LDD/Ub complex (PDB ID: 4LJP). In this drawing, HOIP T731 and its neighboring Q40, L71, L73 residues from the donor ubiquitin are further indicated.

Supplementary Table

Table S1: Statistics of X-ray crystallographic data collection and model refinements

**Data set**	**STK4(11-326) K59R/HOIP(854-1072) complex**
**Data collection**	
Space group	*C2*
Unit cell parameters	
a, b, c (Å)	138.99, 115.77, 118.82
α, β, γ (°)	96.36, 90.00, 90.00
Wavelength (Å)	0.97915
Resolution range (Å)	75.57-2.78 (2.83-2.78)
Number of total reflections	216133 (10007)
Number of unique reflections	32995 (1682)
Redundancy	6.6 (5.9)
$I/\sigma I$	13.7 (2.2)
Completeness (%)	97.5 (98.4)
R_{merge} (%)[a]	8.0 (78.9)
**Structure refinement**	
Resolution (Å)	68.87-2.78 (2.86-2.78)
R_{work}[b]/R_{free}[c] (%)	23.70 (40.27) /28.00 (41.88)
Number of reflections	
working set	32802 (2597)
test set	1640 (126)
B factor (Å^2)	
average	113.64
protein	113.69
RMSD bonds (Å)	0.003
RMSD angles (°)	0.67
Number of non-hydrogen atoms	
protein	7984
ligand	20
water	6
Ramachandran plot (%)	
most favored	95.98
additionally allowed	4.02
outliers	0.00

[a] $R_{merge} = \sum|I_i - I_m|/\sum I_i$, where I_i is the intensity of the measured reflection and I_m is the mean intensity of all symmetry related reflections.

[b] $R_{work} = \Sigma||F_{obs}| - |F_{calc}||/\Sigma|F_{obs}|$, where F_{obs} and F_{calc} are observed and calculated structure factors.

[c] $R_{free} = \Sigma_T||F_{obs}| - |F_{calc}||/\Sigma_T|F_{obs}|$, where T is a test data set of about 5% of the total reflections randomly chosen and set aside prior to refinement.

Numbers in parentheses represent the value for the highest resolution shell.
